# Evaluating the impact of two decades of USAID interventions and projecting the effects of defunding on mortality up to 2030: a retrospective impact evaluation and forecasting analysis

**DOI:** 10.1016/S0140-6736(25)01186-9

**Published:** 2025-07-19

**Authors:** Daniella Medeiros Cavalcanti, Lucas de Oliveira Ferreira de Sales, Andrea Ferreira da Silva, Elisa Landin Basterra, Daiana Pena, Caterina Monti, Gonzalo Barreix, Natanael J Silva, Paula Vaz, Francisco Saute, Gonzalo Fanjul, Quique Bassat, Denise Naniche, James Macinko, Davide Rasella

**Affiliations:** a Institute of Collective Health, Federal University of Bahia, Bahia, Brazil; b ISGlobal, Barcelona, Spain; c Facultat de Medicina i Ciències de la Salut, Universitat de Barcelona, Barcelona, Spain; d Paediatrics Department, Hospital Sant Joan de Déu, Universitat de Barcelona, Barcelona, Spain; e Fundação Ariel Glaser, Maputo, Mozambique; f Centro de Investigação em Saúde de Manhiça, Maputo, Mozambique; g ICREA, Barcelona, Spain; h Institut Clínic de Medicina I Dermatologia, Hospital Clínic de Barcelona, Barcelona, Spain; i CIBER de Epidemiología y Salud Pública, Instituto de Salud Carlos III, Madrid, Spain; j University of California, Los Angeles, CA, USA

## Abstract

**Background:**

The US Agency for International Development (USAID) is the largest funding agency for humanitarian and development aid worldwide. The aim of this study is to comprehensively evaluate the effect of all USAID funding on adult and child mortality over the past two decades and forecast the future effect of its defunding.

**Methods:**

In this retrospective impact evaluation integrated with forecasting analysis, we used panel data from 133 countries and territories— including all low-income and middle-income countries (LMICs)—with USAID support ranging from none to very high. First, we used fixed-effects multivariable Poisson models with robust SEs adjusted for demographic, socioeconomic, and health-care factors to estimate the impact of USAID funding on all-age and all-cause mortality from 2001 to 2021. Second, we evaluated its effects by age-specific, sex-specific, and cause-specific groups. Third, we did several sensitivity and triangulation analyses. Lastly, we integrated the retrospective evaluation with validated dynamic microsimulation models to estimate effects up to 2030.

**Findings:**

Higher levels of USAID funding—primarily directed toward LMICs, particularly African countries—were associated with a 15% reduction in age-standardised all-cause mortality (risk ratio [RR] 0·85, 95% CI 0·78–0·93) and a 32% reduction in under-five mortality (RR 0·68, 0·57–0·80). This finding indicates that 91 839 663 (95% CI 85 690 135–98 291 626) all-age deaths, including 30 391 980 (26 023 132–35 482 636) in children younger than 5 years, were prevented by USAID funding over the 21-year study period. USAID funding was associated with a 65% reduction (RR 0·35, 0·29-0·42) in mortality from HIV/AIDS (representing 25·5 million deaths), 51% (RR 0·49, 0·39–0·61) from malaria (8·0 million deaths), and 50% (RR 0·50, 0·40–0·62) from neglected tropical diseases (8·9 million deaths). Significant decreases were also observed in mortality from tuberculosis, nutritional deficiencies, diarrhoeal diseases, lower respiratory infections, and maternal and perinatal conditions. Forecasting models predicted that the current steep funding cuts could result in more than 14 051 750 (uncertainty interval 8 475 990–19 662 191) additional all-age deaths, including 4 537 157 (3 124 796–5 910 791) in children younger than age 5 years, by 2030.

**Interpretation:**

USAID funding has significantly contributed to the reduction in adult and child mortality across low-income and middle-income countries over the past two decades. Our estimates show that, unless the abrupt funding cuts announced and implemented in the first half of 2025 are reversed, a staggering number of avoidable deaths could occur by 2030.

**Funding:**

The Spanish Ministry of Science and Innovation, UK Medical Research Council, and EU Horizon Europe.

## Introduction

For the past 20 years, the USA has been the leading government donor to humanitarian response plans, development aid, and multilateral development banks, mainly through the US Agency for International Development (USAID). USAID was established in 1961 as an independent agency in the executive branch under the direct authority and guidance of the Secretary of State.^1^ The agency's aim was twofold: the first was to provide humanitarian assistance, and the second to also assist and support economic growth and self-resilience of developing countries, especially those deemed strategic for the US economic and geopolitical impact.^2^ In each mission and country where USAID has been operational, the agency engaged with diverse partners, such as central governments, private entities, local organisations, and international and national non-governmental organisations operating mostly bilaterally. Over the years, and despite a relatively modest effort in relation to its national wealth, the USA's importance as a donor for development and humanitarian aid has overshadowed any other donor. Although the USA has indeed been the largest donor in absolute terms—providing more than US$55 billion in official development assistance (ODA) in 2023 and accounting for approximately 30% of total Development Assistance Committee (DAC) countries' ODA—it ranked only 25th out of 30 DAC members in terms of ODA relative to national income, allocating just 0·24% of its gross national income. By contrast, countries such as Norway (1·09%) and Luxembourg (1·00%) exceeded the 0·7% target of the UN, reflecting a substantially higher proportional commitment to international development.

**Research in context**

**Evidence before this study**

Despite the US Agency for International Development (USAID) being the world's leading donor for humanitarian and development aid, there is scarce evidence in the literature assessing its impact on global health. Few evaluations have attempted to estimate the effects of USAID funding on maternal and child mortality in selected low-income and middle-income countries (LMICs), and some reports have offered only approximate estimates for specific diseases. Since the onset of substantial funding cuts, few studies have evaluated the consequences of reductions in USAID-funded interventions. In a few cases, websites have provided rough estimates on the cumulative effect of these cuts on tuberculosis, child mortality, and other health diseases or conditions.

**Added value of this study**

To our knowledge, this study is the first comprehensive analysis to assess the impact of total USAID funding—including support for health care, nutrition, humanitarian aid, development, education, and related sectors—on mortality in LMICs over the past two decades. Our study also disaggregates effects by age group and cause of death. Importantly, it is the only study to integrate retrospective evaluations with forecasting models that project the effects of current and proposed funding cuts on child and all-age mortality to 2030. We estimate that over the past two decades, USAID-funded programmes have helped prevent more than 91 million deaths globally, including 30 million deaths among children. Projections suggest that ongoing deep funding cuts—combined with the potential dismantling of the agency—could result in more than 14 million additional deaths by 2030, including 4·5 million deaths among children younger than 5 years. These results provide essential evidence for policy makers, planners, and advocates navigating the future of US global health engagement.

**Implications of all the available evidence**

USAID funding has had a crucial role in improving global health, particularly by reducing mortality from poverty-related diseases and saving the lives of millions of adults and children. Current and proposed US aid cuts—along with the probable ripple effects on other international donors—threaten to abruptly halt and reverse one of the most important periods of progress in human development. For many LMICs, the resulting shock would be similar in scale to a global pandemic or a major armed conflict. Unlike those events, however, this crisis would stem from a conscious and avoidable policy choice—one whose burden would fall disproportionately on children and younger populations, and whose consequences could reverberate for decades.

In 2023, the USA accounted for 43% of all government funding donated by countries to the humanitarian system, up from about 39% a decade earlier. USAID has been estimated in 2024 to have managed more than $35 billion.^1^ Although the USA engages in a wide range of sectors, the biggest sectors being funded were humanitarian assistance ($9·9 billion) and health ($9·5 billion), and the largest benefiting region was sub-Saharan Africa ($12·3 billion). However, throughout the years, and according to USAID reports, the agency also funded education projects by investing in teacher training, curriculum development, and school infrastructure, supporting, only between 2011 and 2015, more than 52 million children in 45 countries.^1^

Between 2017 and 2020, the agency responded to more than 240 natural disasters and crises worldwide; in 2016 alone, the organisation provided food assistance to more than 53 million people across 47 countries.^3^ Moreover, USAID has been a supporter of the Global Alliance for Vaccines and Immunization (GAVI) and pledged $1·16 billion over 2020–23 to support the organisation. USAID has also been involved in combating malaria through the President's Malaria Initiative (PMI). USAID is also one of the seven agencies involved in the direct implementation of the President's Emergency Plan for AIDS Relief (PEPFAR), launched in 2003, and investing an accumulated amount of over $100 billion in the global HIV/AIDS response.^3^ In 2023, 60% of PEPFAR's bilateral HIV assistance was obligated and implemented by USAID. It is important to recognise that the USAID funding model— like that of other US federal agencies—is largely driven by political negotiations within Congress and the Senate. Consequently, ensuring the long-term sustainability of funding is inherently challenging, particularly in the absence of strong support from the executive branch.^1^, ^2^, ^4^ For these reasons, a few USAID sectors have been elaborating plans to promote the scalability and durability of their interventions.

On Jan 20, 2025, the Trump administration released the Executive Order 14169, Reevaluating and Realigning United States Foreign Aid, which suspended existing foreign aid programmes, except for emergency food assistance and military aid.^5^ On March 10, 2025, it was announced that 83% of the programmes run by USAID would be cancelled.^6^ These cuts are already being challenged in court, and the outcome of the process is uncertain, at least for the current fiscal year. Assuming the cancellations stand, this could include a potential 88% cut in support to maternal and child health aid, 87% to epidemics and emerging diseases surveillance, and 94% cuts to programming for family planning and reproductive health.^7^

Suspended US contracts for the PMI have halted hundreds of millions of dollars annually to countries such as Nigeria and Uganda, threatening an increase of nearly 15 million additional cases and 107 000 additional deaths globally in just 1 year of a disrupted malaria-control supply chain.^8^, ^9^ The UN World Food Programme has closed its southern Africa office, placing 27 million people at risk of hunger amidst the country's worst drought in decades.^10^ Moreover, a recent survey has estimated that 79 million people previously targeted for assistance are no longer being reached because of USAID programme cuts, and that the local capacity of national non-governmental organisations has been profoundly affected.^11^ If these substantial cuts continue, the vast majority of USAID-funded activities will be affected, with most likely to be terminated.

To date, only a few assessments, most of them not peer-reviewed and focused on health-related USAID programmes, have tried to estimate the impact of USAID and the effects of the current funding cuts on specific diseases.^12^, ^13^, ^14^ This study aims to provide a comprehensive evaluation of the impact of total USAID funding—including support for health care, nutrition, development, humanitarian relief, education, and other sectors—on mortality in low-income and middle-income countries (LMICs) over the past two decades. The analysis includes disaggregated estimates by age group and cause of death and employs forecasting models to project the potential effects of current funding cuts on all-age and child mortality to 2030.

## Methods

### Study design

Our study combined a retrospective impact evaluation (ex-post, covering the period 2001–21) with forecasting analyses (ex-ante, projecting from 2022 to 2030). Both components relied on a shared underlying data structure, study design, and analytical approach.

The impact evaluation had a longitudinal ecological design, whereby countries (unit of analysis) were observed and projected over time. This longitudinal dataset combined aggregated demographic, socioeconomic, health, and USAID data from several sources (all data used in this study are publicly available from the sources listed in the appendix p 10.) during 2001–23. From all countries and territories worldwide, we selected 133 low-income, lower-middle-income, and upper-middle-income countries with consolidated data available up until 2023. Models including all countries, and weighting by population size were also estimated (appendix p 21).

### Study variables

The main dependent variable in the analysis was age-standardised all-cause mortality (ASMR) per 1000 inhabitants. In addition, mortality was examined across different age groups, including child mortality (children younger than 5 years per 1000 live births). Given that USAID interventions are expected to have the greatest effect on children, we further disaggregated under-five mortality into specific age categories: infancy (0–1 year), preschool (2–4 years), child (younger than 5 years), and school age (5–9 years). To assess cause-specific associations, we also selected a set of mortality categories linked to USAID priorities and poverty-related conditions on the basis of existing literature.^12^, ^13^, ^14^, ^15^, ^16^, ^17^, ^18^, ^19^ These associations were defined using the International Classification of Diseases, 10th revision (ICD-10), and included tuberculosis (A15–A19 and B90), HIV/AIDS (B20–B24), maternal causes (O00–O99), lower respiratory infections (J09–J22, P23, and U04), malnutrition (E00–E02, E40–E46, E50, D50–D53, D64.9, and E51–E64), diarrhoeal diseases (A00, A01, A03, A04, and A06–A09), malaria (B50–B54, P37.3, and P37.4), and neglected tropical diseases (A66, A67, A69.1, A71, A77, A78, A79, B55–B56, B57, B65, B66, B73–B74, B76–B77, B79, B83, B88.0, B88.1, and B88). Lastly, specific injury-related mortality (V01–Y89, excluding X41–X42, X44–X45, and U12.9) was included as a negative control, as in previous studies.^20^

Our exposure variable was the financial assistance provided by USAID, in all sectors and areas of interventions, specifically measured as USAID funding per capita. The USAID funding per capita was calculated, similarly to previous studies,^12^, ^15^ by dividing the total disbursed amount (numerator, in monetary values) by the total population (denominator) for each of the 133 countries across each fiscal year from 2001 to 2023. As in previous studies,^20^, ^21^ we categorised the USAID funding per capita to estimate the non-linear dose–response relationship associated with increasing levels of intervention implementation. In the absence of established reference values in the literature—and in line with previous evaluations that classify interventions intensity into three levels (low, intermediate, and high),^20^, ^21^ and considering the countries where USAID mostly operates—USAID funding per capita was categorised using quartiles of its distribution in low-income countries: baseline, considered as not exposed ($0–1·96 per capita per year); low (25th percentile, $1·97–3·96); intermediate (50th percentile or median, $3·97–7·09); and high (75th percentile, $7·10 and higher). This categorical approach was preferred over a continuous specification because the functional form of the dose–response relationship was unknown, and the exposure variable included potential outliers that could disproportionately influence effect estimates in continuous models. Nevertheless, we also tested models with continuous variables and several alternative categorisations (and the results remained robust across all different specifications; more details on these sensitivity analyses and alternative categorisations are available in the appendix pp 18–20).

All relevant time-variant demographic, socioeconomic, and health-care adjusting variables, according to the literature,^12^, ^15^, ^22^, ^23^ were included in the models: gross domestic product (GDP) per capita at purchasing power parity (GDP pc PPP); public expenditures on education, health, and the military (each on as a percentage of GDP); literacy rate; Gini index; the percentage of households with inadequate sanitation and with access to piped water; the number of doctors per 1000 population; and the number of hospital beds per 1000 population. Additional covariates and specifications where also tested in sensitivity analyses (appendix pp 16–33). As in previous studies,^20^, ^21^ to harmonise the model with the categorised exposure variables and to mitigate the influence of potential outliers, we dichotomised these covariates according to their median value over the period. Moreover, we included time dummy variables (for 2008–09, 2013–14, 2015–16, and 2020–21) to adjust for major economic and health shocks that occurred globally in the past two decades.^24^

### Data sources

Data on age-standardised mortality, per cause and age groups, were collected from the Global Burden of Disease Collaborative Network.^24^ The data on USAID funding was collected from US Government agencies reporting foreign assistance.^2^ Demographic, socioeconomic, and healthcare-related variables were obtained from the World Bank data and the WHO Global Health Observatory data.^25^ The complete list of data sources and related detailed methods is presented in the appendix (p 10).

### Statistical analyses

In the retrospective impact evaluation, we estimated the effect of USAID per capita funding during the years 2001–21 on age-standardised mortality, overall and for age-specific and cause-specific groups, using Poisson multivariable regression models with robust SEs and fixed-effects specifications. Additional estimates included sex–age-specific mortality, further stratified by country groupings based on income level, GDP, Gini index, and Human Development Index (HDI). This is a consolidated methodological approach that, accompanied by extensive sensitivity and triangulation analyses, enables the estimation of the effects of interventions on mortality using panel data at the aggregate level.^20^, ^21^, ^22^, ^26^ The fixed-effects models include a term to control for unobserved characteristics of the unit of analysis that are approximately constant during the study period, such as some geographical, historical, infrastructural, or sociocultural aspects of each country, and could be associated both with the outcome and with the intervention implementation.^20^, ^21^, ^22^ To evaluate the robustness of the estimates, we did several sensitivity analyses (appendix pp 13–33). First, to evaluate the influence of the exposure categorisation, we fitted the models by using continuous variables and by changing the number of categories and variable thresholds. Second, to assess the external validity of our estimates—that is, to verify that the findings hold true in a broader context—we fitted the models using all 204 countries and territories in the world. We also tested the effect of population weighting in the regression and assess the effect of excluding highly populated countries, such as China and India. Third, to investigate the influence and relevance of the time trends, we tested different sets of time variables. Fourth, to evaluate the stability of the results with alternative models, we fitted negative binomial regression models and compared their estimates with Poisson models. Fifth, to verify the specificity of the USAID funding-per-capita effects, we fitted the same models with injury-related mortality, used as a negative control.^20^, ^27^ Finally, to reach a higher degree of confidence in the causal inference and the overall impact evaluation, we did triangulation analyses^28^ using difference-in-difference with propensity score matching,^21^, ^26^, ^29^ evaluating the countries with low USAID coverage versus medium and high coverage in the years 2001 and 2021. We used Stata (version 17·0) for database processing and analysis.

For the forecasting analysis, we employed validated country-level microsimulation models to project the health effects of current USAID defunding and its progressive phase-out until 2030. Microsimulation is widely regarded as one of the most accurate forecasting methods, because it enables the incorporation of country-specific characteristics and their associated outcome probabilities into the modelling process. This characteristic is especially true when models are developed using projections on the basis of retrospective real-world cohorts, preserving the original distribution of variables, their intercorrelations, and country-specific trends.^30^ Our modelling approach, based on previous studies,^21^, ^26^, ^31^ was done in two stages: first, we created a synthetic cohort of all countries for the years 2024–30, extrapolating and modelling each country-level independent variable from the retrospective dataset; second, we predicted mortality using these independent variables as inputs in the same multivariate regression models employed in the retrospective analysis, incorporating the effect estimates derived from the ex-post evaluation.

In the first stage, we simulated two USAID scenarios: first, a business-as-usual scenario, keeping USAID funding at the levels of 2023; and second, the currently prospected 83% funding cuts of 2025, and the potential termination of USAID funding from 2026 to 2030 (appendix pp 39–40). In the second stage, for each outcome and each scenario, we did 1000 Monte Carlo simulations, allowing parameter values to vary in each simulation cycle according to their underlying distribution. All-age mortality and under-five mortality across scenarios were compared using mortality rate ratios and absolute differences in the number of deaths, including estimates of the cumulative number of excess deaths over the entire 2025–30 period.

Further details of the modelling process—done in accordance with international model reporting guidelines (ISPOR-SMDM)^32^—are provided in the appendix (pp 39–44). These details also include model calibration, validation, parameter distributions for Monte Carlo simulations, and the model equations. All forecasting analyses were done using R (version 4.1.2).^33^

### Role of the funding source

The funder had no role in the study design, data collection, data analysis, data interpretation, or writing of the manuscript and the decision to submit for publication.

## Results

We calculated the mean values and trends of all the variables in the selected countries over the study period (2001–21; table 1). Overall ASMR started in 2001 at 11·65 (SD 3·46) per 1000 population and decreased by 13% until 2021, whereas the under-five mortality started at 73·71 (46·31) and reduced by 49%. On average, USAID funding increased by 97% (from $1·38 to $2·71 capita), whereas the average funding per country increased by 68% (from $151 million to $253 million). Overall, socioeconomic conditions improved, both in terms of GDP per capita, adequate sanitation, primary education, and other indicators. Health expenditure as a percentage of GDP and the number of physicians also increased. The Gini Index, hospital beds per 1000 population, and a few other indicators showed mild deterioration.

**Table 1:** Descriptive statistics of mortality, USAID funding, and demographic, socioeconomic, and health-care-related variables for selected countries (n=133) from 2001 to 2021

	**2001**	**2011**	**2021**	**Change from 2001 to 2021**
**Mortality rate**				
Overall ASMR per 1000 population	11·65 (3·46)	9·77 (2·90)	10·19 (3·42)	−13%
Children younger than 5 years per 1000 live births	73·71 (46·31)	53·43 (36·97)	37·61 (26·96)	−49%
USAID amount (constant US$)				
Average funding per country (in millions of US$)	151 (209)	279 (420)	253 (293)	+68%
USAID per capita	1·38 (3·85)	2·96 (11·20)	2·71 (7·51)	+97%
**Population size**				
All 133 countries	5 059 516 974	5 832 211 621	6 619 196 694	+31%
All countries without China and India*	2 742 373 742	3 235 429 413	3 781 955 734	+38%
**Other covariates**				
GDP per capita PPP (US$)	5984·6 (5004·5)	9745·2 (6867·4)	13 432·6 (8316·7)	+124%
Gini index	0·55 (0·07)	0·58 (0·06)	0·58 (0·05)	+5%
Health expenditure (% GDP)	4·33 (1·52)	4·32 (1·62)	5·01 (2·18)	+16%
Education expenditure (% in public institutions)	88·65 (1·21)	89·41 (1·10)	88·0 (1·90)	−1%
Military expenditure (% GDP)	2·24 (2·35)	1·93 (2·22)	1·89 (7·56)	−15%
Primary education (% population)†	67·25 (23·50)	69·64 (22·21)	75·61 (19·71)	+12%
Piped water (% population)	77·23 (17·52)	84·14 (15·18)	90·10 (13·10)	+17%
Adequate sanitation (% population)	46·37 (26·61)	62·22 (22·91)	77·47 (22·24)	+67%
Proportion of individuals older than 15 years who are literate (%)	63·6 (15·54)	66·49 (20·72)	69·38 (12·42)	+9%
Fertility rate	3·94 (1·47)	3·37 (1·40)	2·87 (1·25)	−27%
Hospital bed rate per 1000 population	1·70 (1·33)	1·65 (1·45)	1·58 (0·71)	−7%
Rate of physicians per 1000 population	0·99 (0·90)	1·09 (0·94)	1·45 (1·11)	+46%

Data are presented as mean (SD), percentage, or absolute number. Mortality (all ages) is expressed per 1000 population, and mortality for children younger than 5 years per 1000 live births. GDP values are reported in constant US dollars, adjusted for purchasing power parity. ASMR=age-standardized mortality rate. USAID=US Agency for International Development.

* China and India were initially included in the analysis as baseline cases (ie, with no or low per-capita USAID aid). We also did analyses excluding these two countries, with results remaining robust (appendix p 21).

† Primary education refers to the primary completion rate (percentage of the relevant age group, ie, 25 years or older).

We calculated the associations between different levels of USAID funding per capita and decreases in mortality rates by age groups (table 2). High levels of funding were associated with lower mortality, in particular a 15% reduction for overall ASMR, 44% for toddler mortality, and 32% for under-five mortality. An estimated 91 839 663 (95% CI 85 690 135–98 291 626) all-age and all-cause deaths, including 30 391 980 (26 023 132–35 482 636) all-cause deaths in children younger than 5 years were averted by USAID funding over the study period.

**Table 2:** Adjusted rate ratios from multivariable fixed-effects Poisson models for the association between age-standardized mortality and annual USAID funding per capita

		**Overall (ASMR)**	**Childhood school age**			
			Infancy (0–1 year)	Preschool (2–4 years)	Child (<5 years)	School age (5–9 years)
USAID per capita						
	Baseline (mean $ 0·45, 0–1·96)	1 (ref)	1 (ref)	1 (ref)	1 (ref)	1 (ref)
	Low (mean $2·88, 1·97–3·96)	0·94 (0·89–0·99; p=0·029)	0·90 (0·83–0·98; p=0·0058)	0·79 (0·69–0·90; p=0·00028)	0·86 (0·78–0·96; p=0·0019)	0·91 (0·83–0·98; p=0·012)
	Intermediate (mean $5·36, 3·97–7·09)	0·91 (0·85–0·97; p=0·0063)	0·84 (0·73–0·97; p=0·021)	0·72 (0·60–0·87; p=0·00063)	0·80 (0·68–0·93; p=0·0043)	0·88 (0·79–0·99; p=0·034)
	High (mean $20·45, 7·10 or more)	0·85 (0·78–0·93; p=0·0014)	0·74 (0·626–0·88; p=0·0028)	0·56 (0·449–0·69; p<0·0001)	0·68 (0·566–0·81; p<0·0001)	0·80 (0·692–0·93; p=0·014)
Control variables						
	Gini Index	1·06 (1·01–1·10; p=0·0072)	1·09 (0·99–1·19; p=0·064)	1·03 (0·94–1·13; p=0·49)	1·08 (0·99–1·18; p=0·074)	0·95 (0·88–1·02; p=0·19)
	Primary education	0·98 (0·94–1·02; p=0·33)	0·95 (0·88–1·03; p=0·24)	0·95 (0·85–1·06; p=0·37)	0·95 (0·88–1·04; p=0·27)	0·98 (0·90–1·07; p=0·68)
	Education expenditure	0·93 (0·87–0·97; p=0·049)	0·92 (0·87–0·97; p=0·0011)	0·85 (0·78–0·92; p=0·0017)	0·89 (0·84–0·95; p=0·00029)	0·87 (0·81–0·93; p=0·00082)
	Piped water	0·96 (0·94–0·98; p=0·0016)	0·83 (0·79–0·87; p<0·0001)	0·67 (0·61–0·73; p<0·0001)	0·80 (0·77–0·83; p<0·0001)	0·78 (0·71–0·87; p<0·0001)
	Adequate sanitation	0·92 (0·86–0·98; p=0·0052)	0·84 (0·71–0·98; p=0·026)	0·80 (0·67–0·949; p=0·011)	0·83 (0·71–0·98; p=0·027)	0·88 (0·79–0·97; p=0·017)
	Nurses	0·88 (0·85–0·91; p<0·0001)	0·84 (0·77–0·92; p<0·0001)	0·74 (0·68–0·81; p=0·0017)	0·81 (0·75–0·89; p<0·0001)	0·77 (0·73–0·82; p<0·0001)
	Hospital beds	0·99 (0·97–1·02; p=0·78)	1·00 (0·95–1·06; p=0·87)	0·98 (0·91–1·05; p=0·53)	1·00 (0·94–1·06; p=0·97)	1·0 (1·02–1·16; p=0·013)
	Health expenditure	0·94 (0·88–1·01; p=0·014)	0·97 (0·91–1·03; p=0·033)	0·93 (0·86–1·01; p=0·012)	0·96 (0·90–1·02; p=0·071)	0·94 (0·89–1·01; p=0·022)
	Military expenditure	1·08 (1·01–1·15; p=0·022)	1·14 (1·05–1·24; p=0·0029)	1·18 (1·01–1·39; p=0·036)	1·15 (1·04–1·27; p=0·0057)	1·15 (1·03–1·28; p=0·0080)
	Time trend control	Yes	Yes	Yes	Yes	Yes
Total number of deaths prevented by USAID (2001–21)*		91 839 663 (85 690 135–98 291 626)	13 286 197 (11 536 769–15 287 795)	8 665 606 (7 203 360–10 415 507)	30 391 980 (26 023 132–35 482 636)	1 047 777 (936 488–1 173 273)
Percentage of deaths averted relative to total deaths (2001–21)*		7·0%	11·5%	27·4%	17·6%	9·0%
Number of observations		2793	2793	2793	2793	2793
Number of countries		133	133	133	133	133

Data are rate ratio coefficients (95% CI). In the row headers, data are mean (lowest value to highest value). Time shocks are controls for specific years of economic or health crisis (2007–08, 2015, 2020, and 2021). ASMR=age-standardised mortality rate. USAID=US Agency for International Development.

* To estimate the number of deaths averted by USAID between 2001 and 2021, we simulated a counterfactual scenario in which USAID funding was set to zero, while keeping all other variables constant. We predicted coefficient *E(Y_it_ | X*), where *X* represents the set of covariates including the interventions, and *Y*_it_ are the mortality rate at country i, in year t. Thus, 100 000 Monte Carlo simulations were used to get a more accurate CI (appendix pp 31–32).

**Figure 1:**
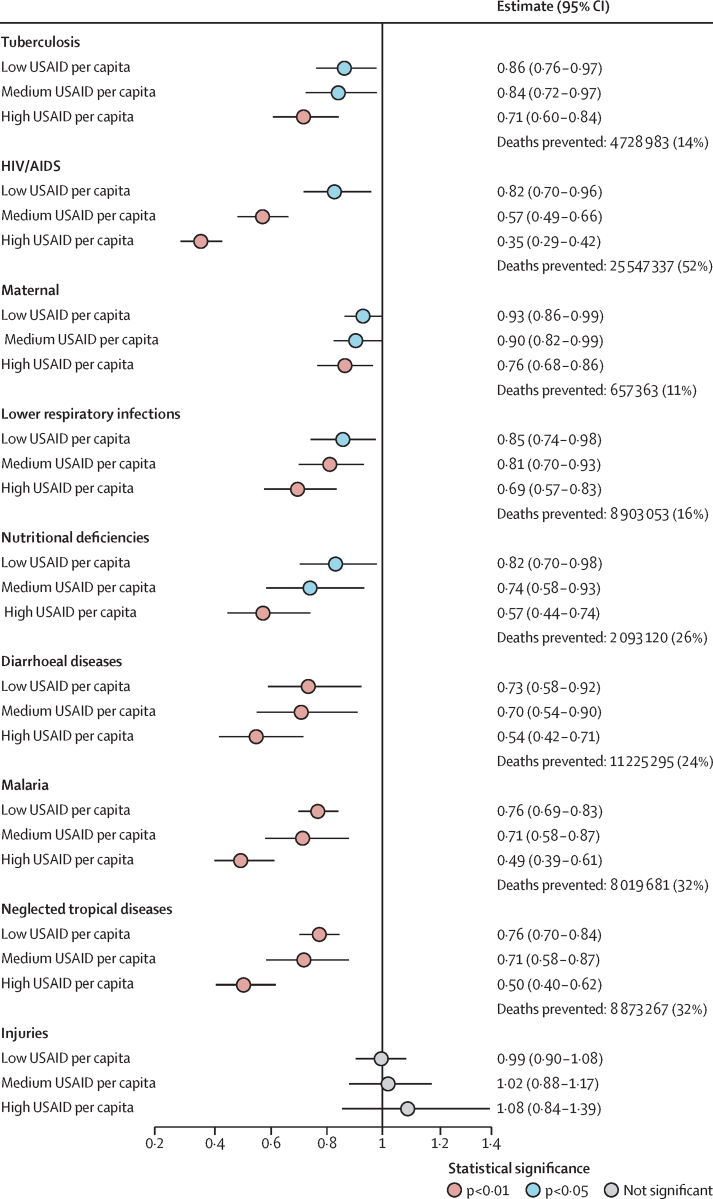
Rate ratios from the fixed-effect Poisson models for the association between specific causes of death related to USAID focus areas and USAID funding per capita per year All models were adjusted for covariates (table 2; appendix p 27). To estimate the number of deaths averted by USAID between 2001 and 2021, we simulated a counterfactual scenario in which USAID funding was set to zero, while keeping all other variables constant. We predicted the coefficient *E*(*Y*_it_ | *X*), where *X* represents the set of covariates including the interventions and *Y*_it_ the mortality at country i, in year t. Thus, 100 000 Monte Carlo simulations were used to get a more accurate CI (appendix pp 37–38). USAID=US Agency for International Development.

We calculated the associations between different levels of USAID funding per capita and decreases in mortality by group of causes (figure 1). The strongest association was found for HIV/AIDS with a 65% reduction, followed by malaria with 51%, and neglected tropical diseases with 50%. Strong associations were also found for diarrhoeal diseases, nutritional deficiencies, lower respiratory infections, maternal mortality, and tuberculosis. No statistically significant association was observed with injuries, used as a negative control.

**Figure 2:**
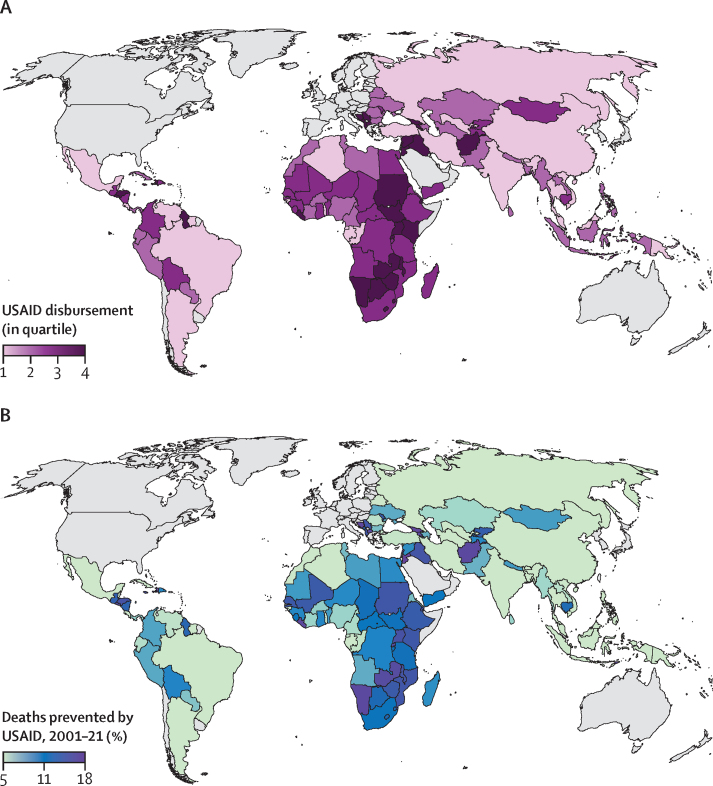
Average per capita USAID disbursement from 2001 to 2021, by quartile, across 133 low-income, lower-income and middle-income, and upper-middle-income countries and territories (A), and deaths (all ages) prevented by USAID implementation as a percentage of the total over the study period 2001–21 USAID disbursement (in quartiles; A) and deaths prevented by USAID, 2001–21 as a percentage (B). USAID=US Agency for International Development.

We calculated the average per-capita USAID disbursement from 2001 to 2021 across the 133 LMICs under study, along with the estimated percentage of total all-age and child deaths prevented over the same period by USAID funding, assuming homogeneous effectiveness across countries (figure 2).

Moreover, we developed stratification analyses evaluating the effects of USAID funding according to countries' characteristics; the effects were stronger in countries with lower HDI, low GDP per capita, and higher Gini Index. Further analyses according to age and sex showed that USAID funding had more effect on women, particularly during their reproductive age (appendix pp 30–31).

The findings withstood all the sensitivity analyses, showing that our results are robust, and all the triangulation analyses, increasing the confidence in the causal interpretation of the statistical associations (appendix pp 13–38).^29^, ^34^ Moreover, we evaluated all sensitivity, triangulation, and complementary results according to the consolidated Bradford Hill causal inference criteria for epidemiological studies (appendix pp 13–16). Finally, we estimated the number of deaths averted by USAID over the past two decades (2001–21), comparing predicted outcomes to a counterfactual scenario without USAID (USAID funding per capita of 0). We also validated these estimates with a prevented fraction for the population analysis (appendix pp 37–38),^34^ which calculates the proportion of deaths averted by comparing observed outcomes under actual USAID disbursements to those predicted in the absence of the intervention.

The microsimulation models (table 3) indicated that, in 2025, reductions in USAID funding per capita would result in approximately 1 776 539 (95% uncertainty interval 967 604–2 496 308) all-age deaths and 689 900 (436 368–911 004) deaths in children younger than 5 years. Over the remainder of the period, the complete defunding of USAID would cause an estimated 2 450 000 all-age deaths annually, leading to a total of 14 051 750 (8 475 990–19 662 191) excess all-age deaths and 4 537 157 (3 124 796–5 910 791) excess under-five deaths by 2030.

**Table 3:** Mortality rate ratios and numbers of avoidable deaths from the comparison of the forecast scenario of USAID defunding versus baseline from 2025 to 2030

	**Number of deaths at all ages**	**Number of deaths in children younger than 5 years**
2025	1 776 539 (967 604–2 496 308)	689 900 (436 368–911 004)
2026	2 499 525 (1 521 410–3 490 070)	828 970 (575 089–1 078 316)
2027	2 477 031 (1 507 936–3 458 985)	798 188 (553 276–1 039 475)
2028	2 454 816 (1 494 286–3 428 201)	768 294 (531 228–1 003 073)
2029	2 432 809 (1 480 850–3 396 272)	739 719 (513 316–962 867)
2030	2 411 030 (1 467 499–3 366 678)	712 098 (494 694–926 596)
2025–30	14 051 750 (8 475 990–19 662 191)	4 537 157 (3 124 796–5 910 791)

Data are number of deaths (95% uncertainty intervals). USAID=US Agency for International Development.

## Discussion

To the best of our knowledge, this study is the first to evaluate the impact of USAID funding—including all areas of its humanitarian and development assistance—on mortality, both overall and disaggregated by age and cause, over the past two decades and with projections to 2030. Our findings show that USAID-supported efforts have helped to prevent more than 91 million deaths across all age groups, including 30 million deaths among children. High levels of USAID funding were associated with a 15% reduction in all- age and all-cause mortality, a 65% reduction in mortality from HIV/AIDS, a 51% reduction from malaria, and a 50% reduction from neglected tropical diseases. Substantial decreases were also observed in mortality from tuberculosis, nutritional deficiencies, diarrhoeal diseases, lower respiratory infections, and maternal and perinatal conditions. By age group, the most pronounced reductions were seen in children younger than 5 years (32%). According to the forecasting models, the current steep funding cuts— coupled with the potential dissolution of the agency—could lead to more than 14 million additional deaths by 2030, averaging more than 2·4 million deaths per year. These deaths include 4·5 million among children younger than 5 years, or more than 700 000 deaths annually.

The strategic objective of USAID has been to administer assistance to nations identified as being of importance to the USA, as well as countries experiencing conflicts.^1^ The agency leads US initiatives aimed at alleviating poverty, combating diseases, and addressing humanitarian needs globally. Additionally, USAID supported US commercial interests by promoting economic growth in developing countries and enhancing their capacity to engage in international trade. Since the 1990s, the health sector has received the most substantial funding from USAID. However, in 2022, humanitarian assistance emerged as the highest-funded sector because of the escalation of global humanitarian crises, in which USAID was responsible for 57·2% of nutrition interventions, 50·0% of food distribution, 54·4% of agricultural interventions, and 41·9% of clean water, sanitation, and hygiene projects. In 2023, governance became the most funded sector, but humanitarian assistance returned to the top position in 2024, followed by health and governance. In the same years, education, agriculture, infrastructure, and economic growth also received relevant USAID investments.^1^

Therefore, the impact of USAID on health and mortality reduction extends beyond its direct funding of health programmes and interventions. A substantial part of its influence stems from improvements in the social determinants of health, particularly among the poorest populations. In particular, USAID's support for poverty alleviation, education, and water and sanitation interventions—among many others—might have had a substantial effect on health outcomes, also considering the broader spillover effects these interventions can have on entire communities. Indeed, poverty alleviation interventions alone have demonstrated important effects on reducing both adult and child mortality. For example, cash transfer programmes have been shown to reduce adult female mortality by 20% and child mortality by 8% in LMICs,^23^ with even greater reductions observed in specific contexts—such as an 18% reduction in under-five mortality in Brazil^20^ and a 24% reduction across Latin America.^26^ Education is another crucial determinant of health. A recent global meta-analysis found that each additional year of education reduces adult mortality risk by 1·9%.^35^ Moreover, mothers who complete secondary education can reduce the mortality risk of their children younger than 5 years by as much as 31%.^36^ Nutritional interventions, especially those targeting malnourished children, have also demonstrated profound effects, significantly lowering child mortality risk.^37^ In addition, interventions aimed at improving access to safe drinking water, sanitation, and hygiene have been shown to reduce child mortality by 17%.^38^ In addition to the effect on mortality through interventions addressing the social determinants of health, USAID also contributes substantially through its support for targeted health promotion, prevention, and treatment initiatives. These initiatives include disease-specific programmes such as PEPFAR for HIV/AIDS and PMI for malaria, both of which rely heavily on USAID for implementation, and partnerships such as Gavi for vaccine-preventable diseases, among many others. Data on funding according to some diseases and conditions, together with a comparison with the deaths averted, are provided in the appendix (pp 32–33).

Previous studies evaluating the effect of USAID interventions in the past decades showed a substantial effect on the reduction of mortality among women of reproductive age,^15^ estimated at 1·3 million over the past two decades, and child mortality, with a reduction of 29 deaths per 1000 live births from 2000 to 2016.^12^ By 2024, PEPFAR was estimated to have saved more than 25 million lives by supporting access to antiretroviral treatments and enabling 7·8 million babies to be born HIV free.^39^ It has been estimated that, together with its partners, PMI has helped to save 11·7 million lives and prevent 1·1 billion malaria cases since 2000.^16^ Studies have attempted to estimate the potential effect of the 2025 USAID funding cuts, focusing on specific health-related interventions, using a variety of methodological approaches, ranging from rapid health impact assessments to advanced infectious-disease modelling. Some estimates suggest that USAID can prevent approximately 3·3 million all-cause, all-age deaths annually, with an uncertainty range of 2·3–5·6 million,^13^ being the majority for HIV/AIDS (1·6 million deaths), tuberculosis (305 997 deaths), malaria (292 998 deaths), and many being prevented by humanitarian relief (548 951 deaths) and vaccinations (501 037 deaths). An impact counter estimated that USAID funding discontinuation caused 62 557 adult deaths and 130 535 child deaths just until mid-April 2025, with an average of 103 deaths per hour, and moving forward, could account for almost 1 million deaths per year.^17^ A modelling study for HIV/AIDS estimated that HIV-related international aid reductions plus discontinued PEPFAR support could cause 4·43–10·75 million new HIV infections and 0·77–2·93 million HIV-related deaths between 2025 and 2030.^14^ Regarding nutritional interventions, some estimates indicate that US-based disbursement for nutrition prevented approximately 600 000 child deaths in 2023 in LMICs,^40^ while others suggest that its reduction for severe acute malnutrition will cut off treatment for millions of children, potentially causing 163 500 child deaths yearly.^41^ A modelling study assessing the potential effect of reductions in USAID's disease-specific and health condition-specific programmes projected that, between 2025 and 2040, such cuts could result in an additional 15·2 million AIDS-related deaths, 2·2 million tuberculosis deaths, and 7·9 million child deaths.^18^

The results of our study—drawing on both retrospective (ex-post) and prospective (ex-ante) evaluations—align with previously cited assessments of deaths averted by USAID. However, our estimates tend to be higher, because they capture not only the direct effects of health-specific USAID interventions, as considered in earlier studies, but also the broader effect of USAID-supported programmes on the social determinants of health. These effects could have substantially contributed to the agency's overall influence on mortality reduction.

Following the announcement of the termination of more than 83% of USAID's programmes, humanitarian organisations have raised serious concerns about the absence of adequate notice or planning for a phased transition. Without time to implement adaptive responses, the most severe effects cannot be mitigated. The short-term, medium-term, and long-term consequences for public health, economic development, and societal stability could be profound.

However, the US administration's decisions compound cuts by other donors, pushing both humanitarian and development systems to the brink of collapse. As a matter of fact, other western donors have announced reductions in aid budgets,^41^ including the UK (40%), France (37%), the Netherlands (30%), and Belgium (25%), which represents a substantial funding crisis in the humanitarian and development aid sector. For 2025, the Organisation for Economic Co-operation and Development estimates official development assistance could drop between 9% and 17%.^42^ These decreases not only threatens to reverse three decades of unprecedented human progress, but also intensifies the extraordinary uncertainty and vulnerability already caused by the ongoing polycrisis.^43^ Although local governments have had a crucial role in the improvements observed over the past decade—often supported by development aid—they will now need to further strengthen their capacities to respond effectively in this new context.

In our study, the substantial reduction in under-five mortality observed in countries receiving the highest levels of USAID funding suggests that an important portion of the global decline in child deaths over the past two decades—primarily in LMICs—could be attributable to humanitarian and development aid. Between 2000 and 2023, the number of global under-five deaths decreased from 10·1 million to 4·8 million.^44^ Our projections indicate that disruptions to such aid could jeopardise this progress, making it increasingly difficult—if not impossible—for the poorest and most vulnerable countries to achieve Sustainable Development Goal target 3.2, reducing the under-five mortality rate to 25 or fewer deaths per 1000 live births by 2030.^45^

This study has several limitations. First, the causal interpretation of the statistical associations must be approached with caution. While the sensitivity and triangulation analyses, along with the application of the Bradford Hill criteria, support a high degree of confidence in a causal interpretation, the study design does not fully eliminate the possibility that the observed associations might not reflect a true causal relationship. Thus, the findings should be interpreted as providing strong support for a causal relationship, though they do not constitute definitive evidence. Another limitation pertains to the use of an aggregate-level analysis, which is subject to ecological fallacy—that is, the inferences drawn are valid at the country level, but not necessarily at the individual level. However, this design can also be considered a strength, as it allows for the inclusion of potential spillover effects—particularly relevant in the context of socioeconomic interventions, which might influence national mortality. Moreover, although the use of a comprehensive set of adjustment variables—based on the relevant literature—and the inclusion of two-way fixed effects (for country and year), along with the results of sensitivity analyses using alternative adjustment sets and the negative control analysis, all support the robustness of the findings and suggest an unbiased estimation of the effects of USAID funding, the potential for residual confounding due to omitted-variable bias cannot be entirely ruled out. Nevertheless, triangulation analyses confirm the presence of a statistically significant effect, and comparisons with other studies support the plausibility of the observed effect sizes.

An additional limitation is that the study cannot disentangle the specific interventions or causal mechanisms through which USAID per capita funding produces its effects. Although the extensive sensitivity and triangulation analyses—along with comparisons to existing literature—reinforce the plausibility of the findings and their magnitude, only alternative study designs (eg, programme-specific evaluations) could precisely evaluate the effectiveness of individual USAID-funded interventions in LMICs. Our study also focused exclusively on evaluating the effects of USAID funding, among all donors involved in humanitarian and development aid, and should be interpreted accordingly. The magnitude of the observed association for USAID funding does not preclude the possibility that other donors or local governments might have similar—or even greater—impacts. For example, the substantial reductions attributed to USAID, such as a 65% decline in HIV/AIDS mortality in countries with high levels of funding, do not imply that other actors cannot have achieved similar results. The magnitude of a rate ratio—reflecting the difference between exposed and unexposed groups for a specific intervention—should not be interpreted as limiting effectiveness of other concurrent or complementary interventions.

Another limitation is that the forecasted scenarios carry inherent uncertainties, driven by two main factors: the difficulty in accurately predicting which specific USAID programmes will be reduced or terminated in the short, medium, and long term; and the inability to account for exogenous shocks, given the current volatility of the global economic and political landscape. Nonetheless, the primary aim of our analysis was not to model in detail the full range of potential USAID defunding scenarios or their varied effects. Instead, it was to estimate the consequences of a worst-case scenario—defined by the most severe funding cuts—relative to a business-as-usual continuation of USAID operations. Importantly, our goal was not to forecast the exact all-age and child mortality by 2030, but to compare two parallel scenarios that differ only in the path of the main exposure variable, USAID funding. Consequently, although external factors such as economic changes, climate events, or conflict might influence future mortality and deviate from our projections, the relative comparisons—such as mortality rate ratios and differences in the number of deaths—are expected to remain valid and robust.

Despite these limitations, the main strength of our study lies in the extensive range of sensitivity analyses done, all of which consistently confirmed the robustness of our findings. Additionally, triangulation analyses using difference-in-differences combined with propensity score matching models provided further validation, strengthening confidence in the causal interpretation of the ex-post impact evaluation associations. Furthermore, the integration of these findings with validated microsimulation models enhanced the reliability of the forecasted scenarios (appendix pp 39–44).

In conclusion, our study demonstrates the crucial role that USAID funding has had in reducing mortality rates across LMICs over the past two decades, and the profound effect that the recent abrupt funding cuts could have on adult and child mortality in the coming years. Beyond causing millions of avoidable deaths—particularly among the most vulnerable—these cuts risk reversing decades of progress in health and socioeconomic development in LMICs, and could substantially undermine the achievement of the 2030 Sustainable Development Goals.

### Contributors

### Data sharing statement

The data used are public and available from the US Government's Foreign Assistance (https://foreignassistance.gov/), the Global Burden of Disease (https://vizhub.healthdata.org/gbd-results/), WHO (https://platform.who.int/mortality), and the World Bank (https://data.worldbank.org/region/world).

## Declaration of interests

We declare no competing interests.
