# Supplementary material for: Evaluating the impact of two decades of USAID interventions and projecting the effects of defunding on mortality up to 2030: a retrospective impact evaluation and forecasting analysis
Source: Lancet. 2025 Jul 19;406(10500):283–94. doi: 10.1016/S0140-6736(25)01186-9 (PMC12274115; doi:10.1016/S0140-6736(25)01186-9)
Supplement: Supplementary appendix [file mmc1.pdf]

THE LANCET

Supplementary appendix

This appendix formed part of the original submission and has been peer reviewed. We post it as supplied by the authors.

Supplement to: Cavalcanti DM, de Oliveira Ferreira de Sales L, Ferreira da Silva A, et al. Evaluating the impact of two decades of USAID interventions and projecting the effects of defunding on mortality up to 2030: a retrospective impact evaluation and forecasting analysis. *Lancet* 2025; published online June 30. https://doi.org/10.1016/S0140-6736(25)01186-9.

Supplementary Materials for:

Evaluating the Impact of Two Decades of USAID Interventions and Forecasting the Effects of Defunding on Mortality up to 2030

TABLE OF CONTENTS

PART I – SUMMARY OF USAID, DATA SOURCES AND GENERAL METHODOLOGY 4

1. THE IMPORTANCE OF USAID IN THE GLOBAL CONTEXT 4

2. JUSTIFICATION OF SCOPE OF STUDY RELATED TO THE USAID 7

2.1. A comprehensive analysis of all USAID investment areas beyond health 7

2.2. Choosing to work with the USAID disbursement per capita 8

2.3. The 133 Countries Selected 8

2.4. The Importance of Using Categorical Variables 8

3. DATASET 10

3.1. Data sources 10

3.1.1. Study Scope: Selected Periods for Retrospective and Forecasting Analyses 10

3.2. Interpolation and extrapolation method 11

PART II – RETROSPECTIVE ANALYSIS 13

4. EMPIRICAL METHODS 13

4.1. Poisson regression – Fixed Effects 13

5. RESULTS 13

5.1. Fit and sensitivity tests 16

5.2. Triangulation – Difference-in-difference with Propensity Score Matching 34

5.3. Deaths prevented by USAID during 2001-2021 37

5.3.1. Validation by Prevented Fraction for the Population method 37

PART III – FORECASTING ANALYSIS 39

6. DESCRIPTION OF THE FORECASTING METHODOLOGY 39

7. PURPOSE OF THE FORECASTING AND ITS APPLICATIONS 39

8. DATA SOURCE AND INPUTS 39

9. POISSON MULTIVARIABLE REGRESSION MODELS WITH ROBUST STANDARD ERRORS AND FIXED-EFFECTS 40

10. CALIBRATION, TIME TREND, AND MONTE CARLO SIMULATION 40

10.1. Calibration 40

10.2. Time trend 41

10.3. Monte Carlo Simulation 43

11. VALIDATION 43

12. MAIN LIMITATIONS 44

PART IV - ASSESSING THE PLAUSIBILITY OF EFFECT MAGNITUDE 45

13. EPIDEMIOLOGICAL PLAUSIBILITY 45

14. ROBUSTNESS OF METHODOLOGY 46

15. COMPARISON WITH EXISTING USAID IMPACT EVALUATIONS 46

REFERENCES 47

LIST OF WEB FIGURE

WEB FIGURE 1. USAID's share of total U.S. grant funding in 2021, by country. 7

WEB FIGURE 2. Framework.. 8

WEB FIGURE 3. USAID per capita disbursement by quartile between 2001-2021, by country. 9

WEB FIGURE 4. Interpolated and Extrapolated control variables boxplot for selected countries (N=133), from the period 2001-21. 11

WEB FIGURE 5. Non-Interpolated or Extrapolated variables boxplot for selected countries (N=133), from the period 2001-21. 12

WEB FIGURE 6. Common support between treatment and control group mortality rates for USAID per capita disbursement.. 34

WEB FIGURE 7. Parallel trends assumption between treatment and control group mortality rates for USAID per capita disbursement. 34

WEB FIGURE 8. Time trend of log mortality rate per 1,000 inhabitants (Excluding 2020-2021). 41

WEB FIGURE 9. Q-Q plot of the residuals from the linear regression model fitted to the log-transformed mortality rate per 1,000 inhabitants. 42

WEB FIGURE 10. Time trend of log under-five mortality rate per 1,000 live births (Excluding 2020-2021). 42

WEB FIGURE 11. Q-Q plot of the residuals from the linear regression model fitted to the log-transformed U5MR per 1,000 live births. 43

WEB FIGURE 12. Simulated Under-Five Mortality Rates with Uncertainty Intervals and Observed Values from UNICEF (2022–2023)... 44

LIST OF WEB TABLES

WEB TABLE 1. American management agencies.. 4

WEB TABLE 2. Key Areas Funded by USAID in 2021 5

WEB TABLE 3. Key Sub-Areas Funded by USAID in 2021... 5

WEB TABLE 4. Data sources and description of variables.. 10

WEB TABLE 5. Analytical strategy used according to Bradford Hill`s criteria. 14

WEB TABLE 6. Hausman test between fixed effect and random effect Poisson models.................... 17

WEB TABLE 7. Rate Ratios from the fixed effect Poisson models for the association between overall mortality rates and USAID per capita, for Omitted variable bias test, from the period 2001-21, in World..................................... 17

WEB TABLE 8. Rate Ratios from the fixed effect Poisson models for the association between overall mortality rates with USAID per capita categorization (different types), from 2001-21, in the World.. 18

WEB TABLE 9. Rate Ratios from the fixed effect Poisson models for the association between overall mortality rates with USAID per capita using continuous variables, from 2001-21 in selected countries/territories......................... 19

WEB TABLE 10. Rate Ratios from the fixed effect Poisson models for the association between overall mortality rates with USAID per capita using categorical variables, from 2001-21, in all countries/territories. 21

WEB TABLE 11. Rate Ratios from the fixed effect Poisson models for the association between overall mortality rates and USAID per capita using categorical variables, with different time shocks control, from 2001-21 in selected countries/territories........ 22

WEB TABLE 12. Rate Ratios for the association between overall mortality rates with USAID per capita, using Negative Binomial models; from 2001-21 in selected countries/territories........ 24

WEB TABLE 13. Rate Ratios from the fixed effect Poisson models for the association between overall mortality rates and USAID per capita using categorical variables, dividing the models by countries' income groups (heterogeneity analyses); from 2001-21 in selected countries/territories. 25

WEB TABLE 14. Rate Ratios from the fixed effect Poisson models for the association between overall mortality rates and USAID per capita per year, for groups of countries according to IHD, GDP and Gini levels........ 26

WEB TABLE 15. Rate Ratios from the fixed effect Poisson models for the association between overall mortality rates and USAID per capita using categorical variables, by different causes of deaths (negative control); from 2001-21, in selected countries/territories. 27

WEB TABLE 16. Rate Ratios from the fixed effect Poisson models for the association between mortality rates and USAID per capita using categorical variables, by different age groups; from 2001-21, in selected countries/territories. 28

WEB TABLE 17. Rate Ratios from the fixed effect Poisson models for the association between mortality rates and USAID per capita using categorical variables, by sex; from 2001-21, in selected countries/territories. 30

WEB TABLE 18. Rate Ratios from the fixed effect Poisson models for the association between mortality rates and USAID per capita using categorical variables, by female age groups; from 2001-21, in selected countries/territories. 31

WEB TABLE 19. Rate Ratios from the fixed effect Poisson models for the association between mortality rates and USAID per capita using categorical variables, by causes of death and USAID health funding; from 2001-21, in selected countries/territories........ 32

WEB TABLE 20. Rate Ratios from the fixed effect Poisson models for the association between mortality rates and USAID per capita using categorical variables, based on USAID's share of total global donor funding; from 2001-21, in selected countries/territories........ 33

WEB TABLE 21. Difference-in-Differences Estimates with Propensity Score Matching of the Association between Intermediate to High USAID Per Capita Disbursements and Mortality Rates (Overall and Child) in 2001 and 2019 among Low- and Lower-Middle-Income Countries/Territories 35

WEB TABLE 22. Rate Ratios from the difference-in-difference fixed effect Poisson models for the association between mortality rates (overall and child) with intermediate to high USAID disbursement, in 2004 and 2019, in Low Income and Lower-Middle Income countries/territories........ 35

WEB TABLE 23. Deaths prevented by USAID disbursement per capita during 2001-21. 37

WEB TABLE 24. Deaths prevented by USAID disbursement per capita during 2001-21 – PFP method. 38

WEB TABLE 25. Final Estimates After Model Refinement and Calibration Based on Minimum MSRE 40

WEB TABLE 26. Comparison between Simulated and Observed Under-Five Mortality Rates (U5MR) in 2022 and 2023 across the 133 Countries. 44

WEB TABLE 27. Distribution of Countries by USAID Funding Quartile, 2001–2021........ 45

PART I – SUMMARY OF USAID, DATA SOURCES AND GENERAL METHODOLOGY

1. THE IMPORTANCE OF USAID IN THE GLOBAL CONTEXT

The agencies and departments of the United States government — including the Department of State (STATE), the Department of Defense (DOD), the Department of Health and Human Services (HHS), among others — play a critical role in the allocation of U.S. foreign assistance.[1] The U.S. Agency for International Development, better known as **USAID**, is the **main external financing** agency of the United States, representing more than 60% of total resources.[1,2] Each entity operates within its specific mandate, supporting initiatives related to international development, global health, democratic governance, security, and humanitarian aid. Collectively, they are central to the execution of U.S. foreign policy objectives, contributing to international stability, economic development in strategic regions, and the promotion of democratic values. U.S. foreign assistance, channeled through these agencies, not only addresses global humanitarian needs but also serves broader national interests by fostering a more secure and prosperous international environment[3]. The Table below summarizes the resources of each of these agencies (values in constant dollars).

WEB TABLE 1. American management agencies

Managing agency acronym	Managing agency name	US$ (Constant price)	%
**USAID**	U.S. Agency for International Development	19,347,156,201	60.3%
**STATE**	Department of State	9,558,191,216	29.8%
**HHS**	Department of Health and Human Services	1,295,132,027	4.0%
**MCC**	Millennium Challenge Corporation	650,399,966	2.0%
**AGR**	Department of Agriculture	641,229,085	2.0%
**DOI**	Department of the Interior	199,891,640	0.6%
**PC**	Peace Corps	123,242,865	0.4%
**DOD**	Department of Defense	101,426,443	0.3%
**TDA**	Trade and Development Agency	32,985,855	0.1%
**ADF**	African Development Foundation	28,575,681	0.1%
**TREAS**	Department of the Treasury	28,270,884	0.1%
**DOL**	Department of Labor	28,165,639	0.1%
**IAF**	Inter-American Foundation	27,698,756	0.1%
**DOE**	Department of Energy	9,454,145	0.03%
**DOJ**	Department of Justice	1,543,774	0.00%
**DOT**	Department of Transportation	264,710	0.00%
**DHS**	Department of Homeland Security	4,475	0.00%

Source: Authors' elaboration based on 2021 USAID data.[1]

USAID has been a central actor in promoting global health, food security, education, and democratic governance, particularly in low- and middle-income countries (LMICs). Since its establishment in 1961, USAID has contributed substantially to addressing critical development challenges by fostering health improvements, economic growth, and institutional resilience.

The range of areas financed with USAID resources is extensive. In 2021, the main area of investment was '**Humanitarian Assistance**', with over US$ 8 billion allocated — USAID accounted for nearly 94% of the total funding from U.S. agencies. 'Peace and Security' ranked second, also receiving more than US$ 8 billion; however, USAID contributed less than 5% to this area. '**Health**' was the third most funded area, with approximately US$ 7 billion invested, of which USAID was responsible for more than 75%. Other key areas where USAID played a significant role include '**Democracy, Human Rights, and Governance**' (86%) and '**Education and Social Services**' (77%). The table below summarises the primary investment areas supported by U.S. agencies, highlighting USAID's substantial contributions.

WEB TABLE 2. Key Areas Funded by USAID in 2021

US Funding Category	USAID	Other US agencies	% USAID
Humanitarian Assistance	$ 7,965,583,317	$ 535,470,754	94%
Democracy, Human Rights, and Governance	$ 1,380,982,009	$ 221,161,965	86%
Education and Social Services	$ 754,393,491	$ 230,499,116	77%
Health	$ 4,955,687,740	$ 1,640,863,459	75%
Economic Development	$ 2,488,825,797	$ 945,142,208	72%
Environment	$ 37,780,700	$ 23,090,245	62%
Program Support	$ 1,356,202,745	$ 872,424,498	61%
Peace and Security	$ 407,700,402	$ 7,977,198,306	5%
Multi-sector	-	$ 280,626,610	0%
**TOTAL**	**$ 19,347,156,201**	**$ 12,726,477,161**	**60%**

Source: Authors' elaboration based on 2021 USAID data.[1]

In terms of sub-areas or specific sectors financed by U.S. agencies, notable highlights include '**Protection, Assistance, and Solutions**', with approximately US$ 8.3 billion invested — 94% of which was provided by USAID — and '**HIV/AIDS**', with over US$ 3 billion invested, of which 66% originated from USAID. Several areas are almost entirely funded by USAID, with contributions ranging from 99% to 100%, including 'Malaria', 'Tuberculosis', 'Maternal and Child Health', 'Family Planning and Reproductive Health', 'Other Public Health Threats', 'Basic Education', and 'Pandemic Influenza and Other Emerging Threats (PIOET)'. The table below presents the 20 most heavily financed sub-areas by U.S. agencies, with particular emphasis on USAID's contributions.[1]

WEB TABLE 3. Key Sub-Areas Funded by USAID in 2021

Rank	US funding sector	USAID	Other U.S. agencies	USAID (%)
01	Protection, Assistance and Solutions	$ 7,793,860,089	$ 499,282,360	94%
02	HIV/AIDS	$ 2,039,253,099	$ 1,070,265,166	66%
03	Direct Administrative Costs	$ 1,319,915,717	$ 859,323,959	61%
04	Macroeconomic Foundation for Growth	$ 953,554,813	$ 20,516,052	98%
05	Malaria	$ 813,719,169	-	100%
06	Good Governance	$ 762,894,572	$ 27,283,038	97%
07	Agriculture	$ 721,650,219	$ 474,081,054	60%
08	Basic Education	$ 572,227,045	$ 4,835,194	99%
09	Pandemic Influenza and Other Emerging Threats (PIOET)	$ 467,606,497	$ 5,136,608	99%
10	Maternal and Child Health	$ 436,214,208	-	100%
11	Conflict Mitigation and Reconciliation	$ 387,789,407	$ 8,432,021	98%
12	Family Planning and Reproductive Health	$ 374,101,765	$ 102,937	100%
13	Water Supply and Sanitation	$ 330,449,705	$ 73,711,862	82%
14	Private Sector Competitiveness	$ 329,805,579	$ 57,997,995	85%
15	Infrastructure	$ 279,602,029	$ 324,515,303	46%
16	Nutrition	$ 274,509,878	$ 230,248,544	54%
17	Civil Society	$ 230,247,646	$ 21,354,770	92%
18	Rule of Law and Human Rights	$ 214,911,040	$ 138,656,499	61%
19	Tuberculosis	$ 183,515,895	-	100%
20	Political Competition and Consensus-Building	$ 172,928,751	$ 331,362	100%

Source: Authors' elaboration based on 2021 USAID data.[1]

In the field of global health, USAID's initiatives have been particularly influential in combating infectious diseases such as **HIV/AIDS** and **malaria**. In this sense, USAID is also one of the seven agencies involved in the direct implementation of the **President's Emergency Plan for AIDS Relief** (**PEPFAR**),[4] launched in 2003, and investing an accumulated amount of over $100 billion in the global HIV/AIDS response.[3] In 2023, 60% of PEPFAR's bilateral HIV assistance was obligated and implemented by USAID. This percentage in 2021 was 66%, as shown in the Table above.

Malaria control constitutes another cornerstone of USAID's health agenda. Through the **President's Malaria Initiative (PMI),**[5,6] USAID has supported the widespread distribution of insecticide-treated mosquito nets, implementation of indoor residual spraying campaigns, promotion of rapid diagnostic testing, and provision of effective antimalarial treatments. These interventions have led to measurable declines in malaria incidence and mortality, especially among children under five years of age (USAID, 2022).

USAID has also prioritized improving **food security** and **nutrition** through programs such as Feed the Future. These efforts aim to reduce hunger and undernutrition by promoting agricultural innovation, strengthening food systems, and enhancing community resilience to shocks. By addressing the underlying determinants of food insecurity, USAID's initiatives contribute to improved health outcomes and poverty reduction (Feed the Future, 2022).

Maternal and child health is another critical focus area. USAID supports interventions designed to reduce preventable maternal, neonatal, and child deaths by improving access to quality health services during pregnancy, childbirth, and early childhood. Key strategies include strengthening primary health care systems, expanding immunization coverage, promoting early childhood nutrition, and supporting community-based health initiatives (USAID, 2021).

Beyond the health sector, USAID's programs advance broader development objectives. In **education**, the agency works to expand access to quality learning opportunities, with a particular emphasis on marginalized populations, including girls and children affected by conflict. Strengthening educational systems is recognized as essential for promoting economic growth, reducing inequalities, and supporting democratic governance (USAID Education Policy, 2018).

Furthermore, USAID engages in initiatives supporting **human rights**, disaster response, climate resilience, and democratic institution-building. These cross-sectoral efforts underscore the agency's commitment to promoting sustainable development and advancing the global Sustainable Development Goals (SDGs).

In summary, USAID's multifaceted interventions have contributed significantly to improving health and development outcomes in LMICs. By integrating health, education, economic, and governance strategies, USAID not only addresses immediate humanitarian needs but also builds the foundations for long-term social and economic resilience. Its ongoing role remains critical in the pursuit of global equity and sustainable development.

However, on January 20, 2025, the Trump administration released the '**Executive Order 14169, Reevaluating and Realigning United States Foreign Aid**', which suspended existing foreign aid programs, except for emergency food assistance and military aid.[7] On March 10, it was announced that **83% of the programs run by USAID would be cancelled**.[8] These cuts are already being challenged in court, and the outcome of the process is uncertain, at least for the current fiscal year. Assuming the cancellations stand, this could include a potential 88% cut in support to maternal and child health aid, 87% to epidemics and emerging diseases surveillance, and 94% cuts to programming for family planning and reproductive health.[2] Currently, the cessation of U.S. funding for Tuberculosis (TB) programs has forced 25% of organizations across 31 countries to shut down TB program operations, with an additional 46% stopping TB screening and outreach.[9] Suspended U.S. contracts for the U.S. President's Malaria Initiative have halted hundreds of millions of dollars annually to countries like Nigeria and Uganda, threatening an increase of nearly 15 million additional cases and 107,000 additional deaths globally in just one year of a disrupted malaria-control supply chain.[6] The UN World Food Programme has closed its **Southern Africa office**, placing 27 million people at risk of hunger amidst the country's worst drought in decades.[10] If these profound cuts continue, the vast majority of USAID-funded activities will be impacted, with the most likely to be terminated.

The quarterly report by the Lead Inspector General for Operation Inherent Resolve,[11] covering the period from January 1 to March 31, 2025, highlights the significant impacts of this executive order issued on January 20 by President Donald J. Trump, which imposed an immediate 90-day pause on U.S. foreign assistance. This measure led to the suspension of most USAID and State Department assistance programs in **Iraq and Syria**, including the halting of third-party monitoring activities. **The rapid implementation of the pause**, coupled with personnel reductions and inconsistent communication, caused confusion within USAID regarding which programs were subject to suspension.

In summary, the Trump administration's recent decision to slash over 80 percent of USAID's foreign aid contracts has had far-reaching and devastating impacts globally—hitting the **Global South** hardest, with especially severe repercussions across the **African continent**.[4,12] Countries such as **Uganda, Ethiopia, South Sudan**, and the Democratic Republic of the **Congo**—heavily reliant on foreign assistance for essential health and food security services—are now facing deepened humanitarian crises. Critical programs like PEPFAR, which has saved millions of lives in the fight against HIV/AIDS, were abruptly halted, leaving millions without access to essential healthcare and treatment.[4] Furthermore, the suspension of early warning systems for natural disasters and food crises has severely undermined emergency response capacity, increasing the risk of humanitarian catastrophes.[12] The map below illustrates the importance, in percentage terms, of USAID resources relative to the total resources from all U.S. agencies.

WEB FIGURE 1. USAID's share of total U.S. grant funding in 2021, by country.

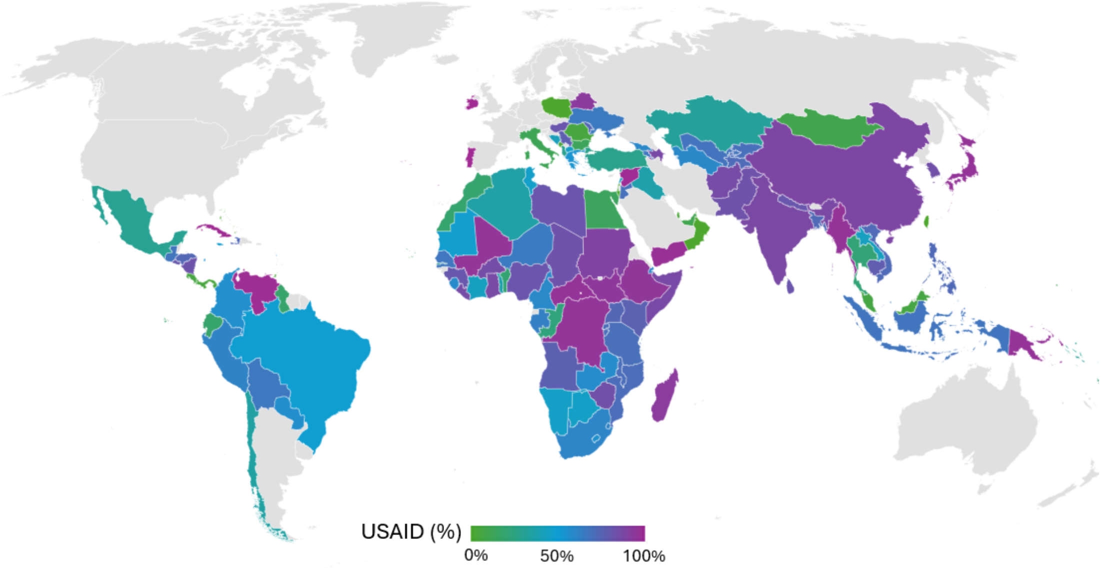

Source: Authors' elaboration based on 2021 USAID data.[1]

2. JUSTIFICATION OF SCOPE OF STUDY RELATED TO THE USAID

2.1. A comprehensive analysis of all USAID investment areas beyond health

Although issues such as education, human rights, and other social conditions may not appear directly related to health outcomes at first glance, they are integral components of the broader framework of social determinants of health. These factors shape the environments in which people are born, grow, live, work, and age, influencing access to resources, opportunities, and services essential for maintaining health. Inadequate education, violations of human rights, and social exclusion contribute to structural inequalities that increase vulnerability to poverty-related diseases. By limiting access to healthcare, reducing health literacy, and perpetuating cycles of deprivation, these conditions function as upstream risk factors that ultimately affect the incidence, distribution, and burden of disease, particularly among marginalised populations.

For this reason, the present study adopts a **comprehensive approach** by analyzing the **full scope of USAID investments**, rather than focusing solely on health-related expenditures as is common in much of the existing literature. Restricting the analysis to the health sector risks overlooking the broader set of interventions that shape health outcomes indirectly but significantly. Investments in areas such as education, gender equality, governance, and economic development can produce substantial health co-benefits by addressing root causes of vulnerability and enhancing population resilience. Therefore, examining the totality of USAID's engagement provides a more holistic understanding of how development aid contributes to health equity and the prevention of poverty-related diseases.

2.2. Choosing to work with the USAID disbursement per capita

The U.S. government budget cycle includes several key stages: the **President's Budget Request**, which outlines the administration's funding priorities; the **Appropriated and Planned** phase, when Congress approves specific budget allocations; **Obligations**, when funds are legally committed to projects or contracts; and finally, **Disbursements**, when money is actually spent.[1]

We chose to use **USAID Disbursements per capita** because it reflects the actual amount of funding **effectively delivered** to a country relative to the **size of its population**. This adjustment is crucial for meaningful comparisons across countries and over time, as it accounts for differences in population size and helps assess the potential impact of aid on individuals.

2.3. The 133 Countries Selected

From a total of 204 countries and territories worldwide, we selected 133 low-, lower-middle-, and upper-middle-income countries (LICs, LMICs, and UMICs) with consolidated data available through 2023.

The exclusion of the 71 high-income countries (HICs) is justified for two main reasons. First, these countries are not the primary focus of USAID donations, which are directed toward low- and middle-income countries. Second, HICs exhibit very different socioeconomic and health dynamics compared to the countries in focus, which could introduce bias and undermine the comparability of the results. Therefore, this exclusion is crucial for ensuring the internal validity of the study, maintaining a more consistent and relevant scope for the countries under analysis.

2.4. The Importance of Using Categorical Variables

In this study, we chose to use categorised variables for several reasons. *First*, they provide more understandable and actionable implementation thresholds, which are valuable for policy-making purposes. *Second*, they help mitigate the influence of over-dispersed values or outliers in the existing or extrapolated data for the independent variables, including the main exposures and covariates. *Third*, and most importantly, when the main exposure variable is a categorical dummy with multiple levels, it allows for a clearer evaluation of the **dose-response** relationship between the exposure and outcome variables, particularly when the relationship is non-linear. In this context, the dose-response analysis explores how varying levels of USAID per capita (the "dose") correspond to changes in age-standardized mortality rates (the "response"). This approach, commonly used in epidemiology and public health,[13–22] helps assess whether higher program coverage results in progressively stronger effects, providing further evidence for a causal interpretation of the statistical associations.

We chose to use a categorical exposure variable, while also verifying that similar effects were observed when using a continuous exposure variable as well as multiple alternative categorizations, based on the following considerations:

1) The functional form of the dose-response relationship was not known
2) The exposure variable contains outliers that could disproportionally affect the effect estimation in the continuous models.
3) The categories of the exposure variable correspond to levels of intensity of the USAID funding, making the corresponding Rate Ratios more easily interpretable, as it has been done in many studies to evaluate the impact of interventions with aggregate-level data.[16,19,21,22]

Additionally, the decision to use the quartile distribution of USAID disbursement per capita in low-income countries reflects the agency's strategic focus on these settings. This classification better captures the context in which USAID primarily operates, enabling a more accurate interpretation of funding patterns. Restricting the reference group to low-income countries also minimizes distortions that could arise from comparisons with middle- or high-income countries, which generally receive less external assistance and have greater fiscal capacity. The figure below presents a world map that visually summarizes the outcome of all the methodological choices made in this study.

WEB FIGURE 3. USAID per capita disbursement by quartile between 2001-2021, by country.

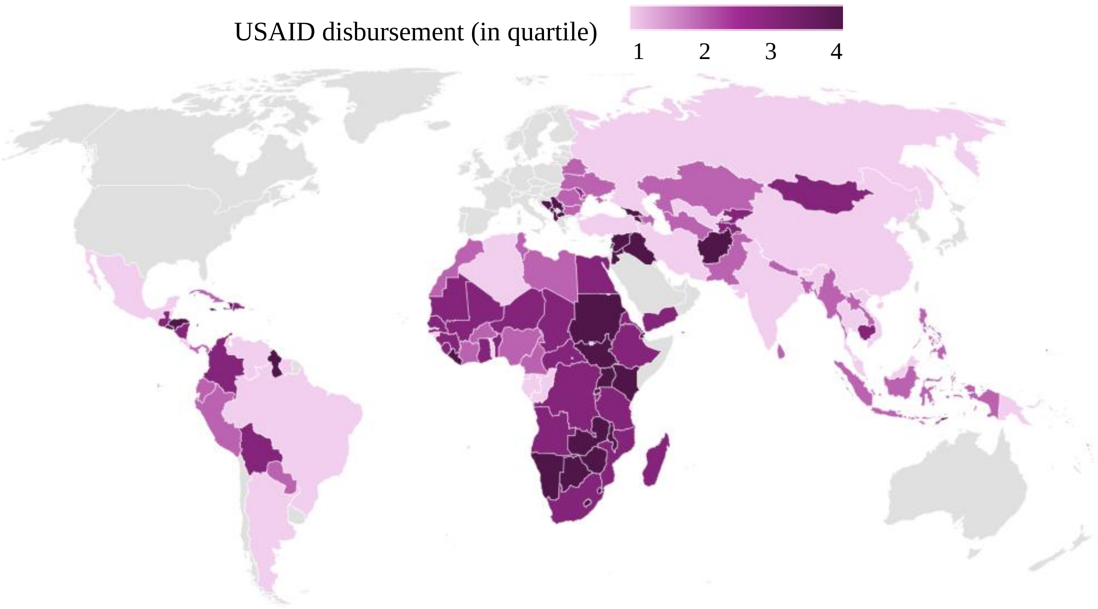

Source: Authors' elaboration based on 2020-2021 USAID data.[1]

3. DATASET

3.1. Data sources

The data used in this study were obtained from various organizations platforms detailed in Web Table 4. All the variables used in this study are aggregated to the country/ territory level. However, the data for some variables were not available for specific years and countries, therefore, we performed an exponential decay method for interpolation, as detailed in Section 3.2. of this supplementary document.

WEB TABLE 4. Data sources and description of variables

Variable	Years	Units of Analysis	Source	Link
Mortality	1980 to 2021	Country/Territory level	GBD	Link 01
USAID disbushment amount	2001 to 2024	Country/Territory level	USAID	Link 02
Population estimates	1980 to 2021	Country/Territory level	GBD	Link 03
GDP per capita PPP (US$)	1960 to 2023	Country/Territory level	World Bank	Link 04
Gini Index	1960 to 2023	Country/Territory level	World Bank	Link 05
Literacy rate (% population)	1976 to 2023	Country/Territory level	UNESCO	Link 06
Primary education (% population)	1970 to 2023	Country/Territory level	UNESCO	Link 07
Piped Water (% population)	2000 to 2022	Country/Territory level	WHO	Link 08
Adequate sanitation (% population)	2000 to 2022	Country/Territory level	WHO	Link 09
Hospital bed rate (per 1,000 population)	1970 to 2021	Country/Territory level	WHO	Link 10
Rate of physicians (per 1,000 population)	1990 to 2021	Country/Territory level	WHO	Link 11
Current health expenditure (% of GDP)	2000 to 2022	Country/Territory level	WHO	Link 12
Military expenditure (% of GDP)	1960 to 2023	Country/Territory level	SIPRI	Link 13
Education expenditure (% of GDP)	2000 to 2023	Country/Territory level	UNESCO	Link 14
Donor participation in official development assistance (ODA)	2015 to 2024	Country/Territory level	OECD	Link 15

Note: GDP = Gross Domestic Product. PPP = Purchasing Power Parity. USAID = U.S. Agency for International Development. GDB = Global Burden of Disease. UNESCO = United Nations Educational, Scientific and Cultural Organization. WHO = World Health Organization. SIPRI = Stockholm International Peace Research Institute. OECD = Organisation for Economic Co-operation and Development.

3.1.1. Study Scope: Selected Periods for Retrospective and Forecasting Analyses

The total analysis period spans from 2001 to 2030, divided as follows:

- **Retrospective Analysis:** This covers the period from **2001 to 2021**.
  - The starting year, 2001, was chosen as it marks the first year with available data on USAID disbursements by country. Although obligation data existed in earlier years, it does not guarantee that the USAID funds were actually spent or disbursed by the countries, as explained previously.
  - The final year, 2021, was selected as it corresponds to the most recent Global Burden of Disease (GBD) mortality data.

- **Forecast Analysis:** This refers to the period from **2022 to 2030**.
  - The starting year: While it is possible to download USAID data for 2024 and 2025, these datasets are not yet consolidated. Therefore, the analysis is based on data up to 2023. The 2024 data was extrapolated from recent trends, and the simulation of the cut-off began in fiscal year 2025, following 'Executive Order 14169: Reevaluating and Realigning United States Foreign Aid.' Further details are presented in ‘PART III - FORECAST ANALYSIS’ of this appendix.
  - The final year, 2030, was selected to align with the target date for achieving the Sustainable Development Goals (SDGs).

3.2. Interpolation and extrapolation method

Some of control variables are not available in total period of 2001-21. In these cases, we use the exponential decay method to extrapolate the variables available at least two points of time, and we drop countries with only one information. We emphasise that none of the outcome variables (mortality) or exposure variables (USAID amount) were interpolated. In the end, it was observed that the interpolated/extrapolated variables improved the control and precision of the retrospective and predictive models.

WEB FIGURE 4. Interpolated and Extrapolated control variables boxplot for selected countries (N=133), from the period 2001-21.

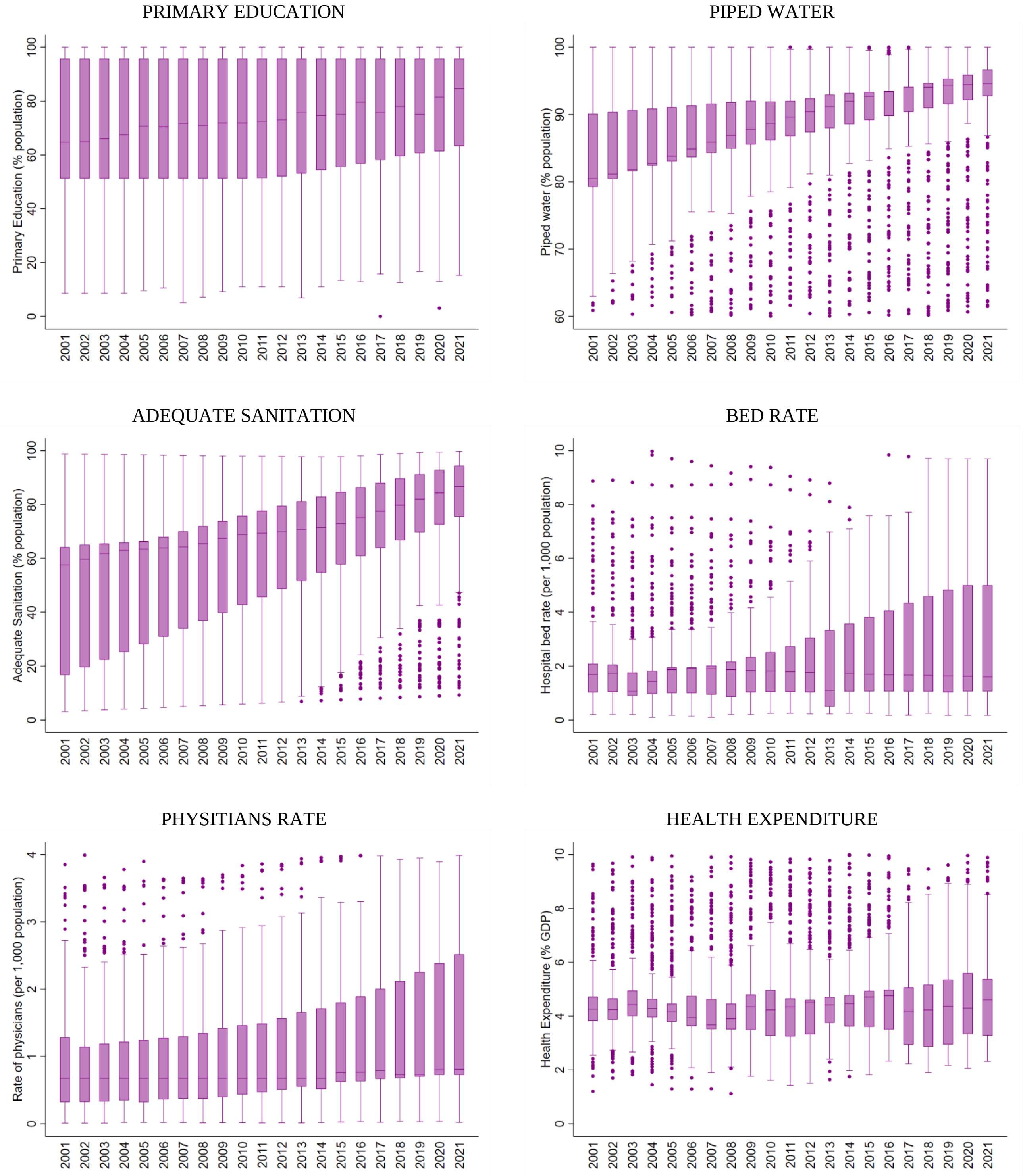

MILITARY EXPENDITURE

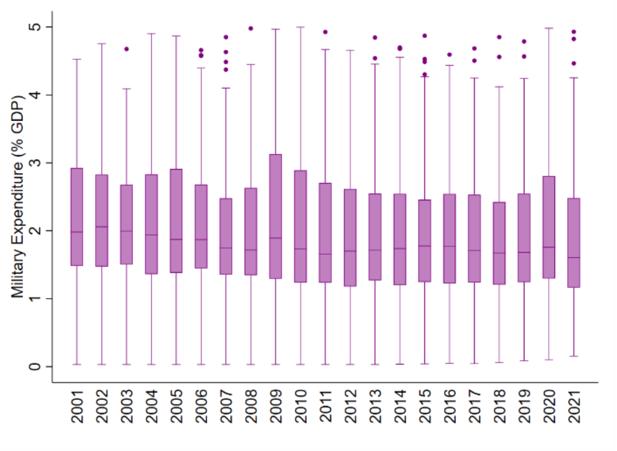

WEB FIGURE 5. Non-Interpolated or Extrapolated variables boxplot for selected countries (N=133), from the period 2001-21.

USAID disbursement per capita (US$)

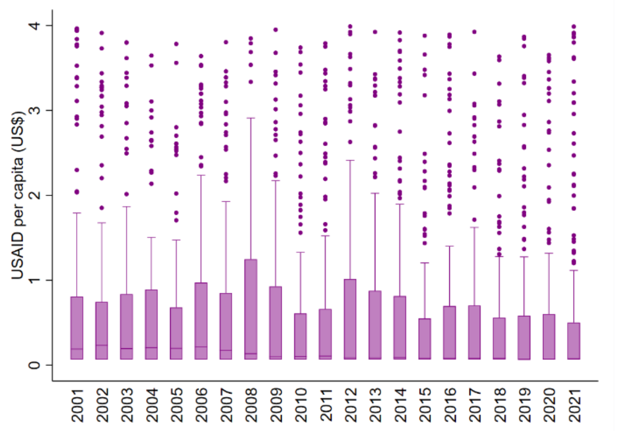

Age-standardized mortality (ASMR)

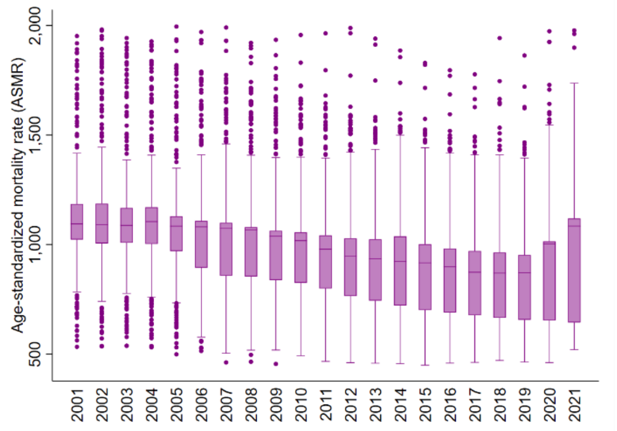

GDP per capita PPP (US$)

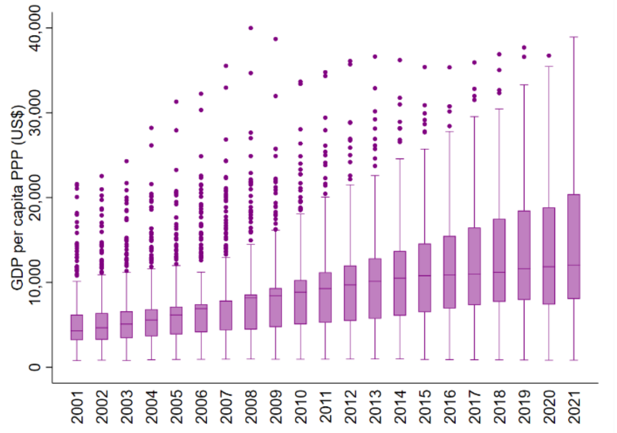

Literacy rate (%)

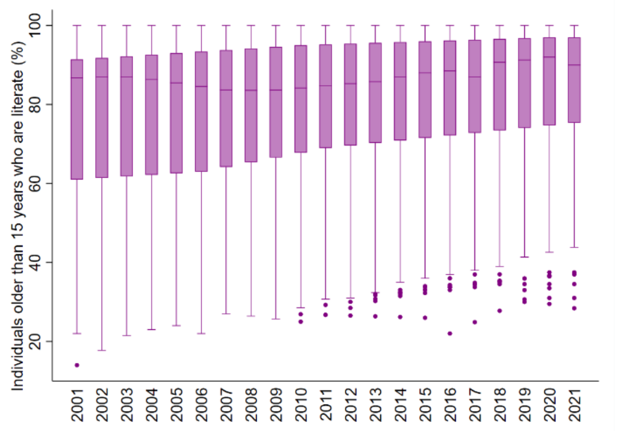

PART II – RETROSPECTIVE ANALYSIS

4. EMPIRICAL METHODS

4.1. Poisson regression – Fixed Effects

We estimate fixed-effect models using the Poisson method with robust standard errors to retrospectively evaluate and forecast the impact of the USAID amount on health outcomes. The equation which describes the linear relationship between the health outcomes (mortality rates) and covariates is given by:

$$log\ (\mu_{it})\ = \log(pop_{it}) + \sum_{q=0}^{3} \beta_q USAID_{qit} + \sum_{s=4}^{8} \beta_s T_y + \sum_{k=9}^{18} \beta_k X_{kit} + \alpha_i + u_{it}$$

where:

- t denotes the year, i represents an individual country, and q indexes the categories of per capita USAID amounts.
- μ_{it} represents the expected number of events of interest— death count—observed in country i in year t. The death numbers used in this study are:
  - Age-standardized (i.e., all ages and all causes) – main outcome,
  - by different **age groups**, including child mortality (children under five per 1,000 live births), adult mortality (up to 54 years), and older adult mortality (55 years and older).
  - by **causes of deaths** linked to USAID priorities and poverty-related conditions based on existing literature.[4–6,9,10,23–28] These were defined using the International Classification of Diseases, 10th revision (ICD-10), and included:
    - tuberculosis (A15–A19, B90),
    - HIV/AIDS (B20–B24),
    - maternal causes (O00–O99),
    - lower respiratory infections (J09–J22, P23, U04),
    - malnutrition (E00–E02, E40–E46, E50, D50–D53, D64.9, E51–E64),
    - diarrheal diseases (A00, A01, A03, A04, A06–A09), malaria (B50–B54, P37.3, P37.4), and
    - neglected tropical diseases (NTDs, ICD-10: A66, A67, A69.1, A71, A77, A78, A79, B55–B56, B57, B65, B66, B73–B74, B76–B77, B79, B83, B88.0, B88.1, B88).
- pop_{it} are the population of interest size in the time *t* for the observation *i.*
- $USAID_{qit}$ are the USAID per capita in categories (quartile, 4 groups) observed at the country i in year t, with associated coefficients β_q. The categories are defined as follows:
  - $q = 0$: Extreme low or no USAID per capita (baseline ≈ US$ 0.45 per capita),
  - $q = 1$: Low (25th percentile ≈ US$ 2.88 pc),
  - $q = 2$: Intermediate (50th percentile or median ≈ US$ 5.36 pc), and
  - $q = 3$: High (75th percentile ≈ US$ 20.45 pc).
- T_y are dummy variables representing previous crisis events with coefficients $\beta_4, \beta_5, .., \beta_8$ from the specific years y, with y= 2008, 2015, 2019, 2020 and 2021 respectively.
- X_{kit} represents different control covariates, each one with a coefficient of β_k (GDP per capita PPP, Gini Index, Primary Education, Education expenditure, doctor rates, hospital bed rate, Health expenditure, Military expenditure, Proportion of individuals living in households with inadequate sanitation, and Piped water).
- α_i is the fixed effect (time-invariant) term for each country/territory, and u_{it} was the error term.

5. RESULTS

This section outlines the more than ten sensitivity and triangulation[29] analyses conducted to ensure the robustness of our findings. It is important to note that the selection of these analyses was not arbitrary;

rather, it was guided by well-established principles of causal inference widely accepted in the health literature, including the **Bradford Hill criteria**.[30]

In epidemiology, causality is established according to several criteria, the oldest and most consolidated are Bradford Hill`s criteria, which include 1) Strength of the association, 2) Consistency, 3) Specificity, 4) Temporality, 5) Biological gradient, 6) Plausibility, 7) Coherence 8) Experiment 9) Analogy. Our analytical strategy was developed to strengthen, as much as possible, any causal inference from the statistical associations we found through each of these 9 criteria, as summarized in the table below.

WEB TABLE 5. Analytical strategy used according to Bradford Hill`s criteria.

Bradford Hill`s criteria		Analytical strategy used in the paper
**Criteria**	**Meaning**	
1. Strength of the association	The stronger the association between exposure and effect (e.g., high relative risk), the more likely it is to be causal.	The **strong effect** and statistical significance between high USAID per capita amounts and reductions in overall mortality ensure compliance with Bradford Hill's first criterion.
2. Consistency	If different studies, across various populations and settings, observe the same association, it strengthens the causal hypothesis.	To evaluate the external validity of our estimates, we refitted the models using data from all 204 countries and territories (Web Table 10). We also stratified the models by countries' income groups (Low-Income, Lower-Middle-Income, and Upper-Middle-Income; Web Table 13), and disaggregated the results across more detailed age groups (Web Table 15). These heterogeneity analyses confirmed that the association between USAID disbursement and overall mortality reduction is broadly consistent across settings and population segments.<br><br>Moreover, to examine the stability of the results under different model specifications (consistency), we fitted Negative Binomial regression models and compared their AIC and BIC values with those of the Poisson models (Web Table 12). The direction and significance of the results remained consistent across models, which strengthens the case for a robust association
3. Specificity	If a specific exposure leads to a single effect (or a limited number of effects), this supports causality.	We estimated the same models using as outcome age-standardized mortality due to External Causes (ICD10 codes V01-Y89), which is considered in epidemiology a **negative control**[31] because USAID are not expected to influence this group of causes and do not include actions for its prevention (see Web Table 14).<br><br>The absence of significant effects in this domain supports the specificity of our main findings and reinforces their coherence.
4. Temporality	The exposure must precede the effect in time (a fundamental principle for establishing causality).	The use of **longitudinal panel data** of **two decade** insure the evaluation of temporal relationships. Furthermore, we tested alternative specifications for the **time variables** to assess whether the results were driven by temporal confounding or artefacts of model specification (Web Table 11). The findings were stable across these variations, supporting temporal consistency.<br><br>These controls, combined with country **fixed-effects** models and other isolation factors, help refine the relationship between USAID per capita and changes in mortality over time.
5. Biological gradient	(Dose-Response Relationship) – If increased exposure levels correspond to a greater risk of the effect, this supports causality.	The choice of working with **categorical** variables enabled **dose-response** analysis between different levels of intervention (from lowest to highest) and mortality. The gradient (dose-response) seen in the effects of coverage

Bradford Hill`s criteria		Analytical strategy used in the paper
Criteria	Meaning	
		levels for each one of the interventions in almost all considered outcomes.<br><br>Additionally, we estimated models using the **continuous measure** of USAID per capita disbursement (Web Table 9). The consistency in direction and magnitude of the effects across both categorical and continuous specifications provides **strong support for a dose-response relationship**, in line with Bradford Hill's biological gradient criterion.
6. Plausibility	The proposed causal relationship should align with existing scientific knowledge and biological mechanisms.	The plausibility of USAID's effects on reducing mortality rates is illustrated in the **framework of Web Figure 2**, in Research in Context (page 25 of the manuscript), and is well established, having been demonstrated in previous studies.[2–6,23–28]<br><br>Furthermore, by testing different **model specifications** (e.g., omitting covariates or including them; see Web Table 7), and verifying that the relationship between USAID and mortality remains plausible and aligned with expected **theoretical mechanisms**, we address the criterion of plausibility. The analogy with similar types of interventions (external aid impacting health outcomes) further supports this.
7. Coherence	The relationship should be consistent with what is already known about the disease or effect being studied.	This criterion refers to the replication of the causal association in different studies, populations, periods and methods. Additionally, studies specifically analyzing USAID in relation to similar outcomes are extensively cited in the "Discussion" section (see page 10-13).<br><br>By performing **different methodological strategies** (e.g., **fixed-effects** panel models, **Negative Binomial** regressions, changed the exposure variable from a categorical specification to a **continuous measure**, **triangulation** with DiD with PSM, among others), we enhance the coherence and consistency of the evidence. That is, the convergence of results across methods with different assumptions and sources of potential bias strengthens the overall causal argument.
8. Experiment	If experiments (such as clinical trials or natural experiments) confirm the association, this strengthens the causal inference.	While this criterion establishes that randomized experiments generally offer stronger causal inference than other study designs, it also acknowledges that even randomized studies may yield weak causal conclusions depending on their design specifications. More importantly, it does not exclude the possibility of deriving causal inference from quasi-experimental or observational designs. Carefully conducted, robust observational studies that adhere to causality criteria and include comprehensive sensitivity and triangulation analyses can provide a sufficient level of causal inference.[32]<br><br>However, our combination of **DiD with PSM** (triangulation analysis, see Web Table 16 and 17) serves as a **quasi-experimental** approach, simulating conditions of a randomized controlled trial by reducing selection bias (through matching) and controlling for time-invariant unobserved confounding (through differencing). This strengthens the **internal validity** of the results and supports the causal interpretation of the association between USAID and mortality reduction.

Bradford Hill`s criteria		Analytical strategy used in the paper
Criteria	Meaning	
9. Analogy	If a similar association has been demonstrated between another factor and a comparable effect, it may suggest causality.	The effects of USAID programs in other countries have been documented in previous studies, as referenced in the "Research in Context" section of the manuscript (see page 3).<br><br>Moreover, our findings are also supported by the criterion of analogy, as similar international aid interventions have demonstrated positive effects on population health outcomes in low- and middle-income countries.[26] The consistent direction and magnitude of our results—observed through multiple robustness checks, including fixed-effects models, negative binomial regressions, and difference-in-differences with propensity score matching—mirror findings from other studies assessing the impact of targeted financial aid on health indicators. Moreover, the observed association between higher USAID disbursement and lower overall mortality is analogous to well-documented effects of other large-scale development programs, such as the Global Fund or **PEPFAR**,[4,26] further reinforcing the plausibility of our conclusions.

5.1. Fit and sensitivity tests

Concerning our study about the effect of USAID on overall mortality, we developed several sensitivity tests to guarantee the robustness of our results.

As a first test for choosing between a random and a fixed effect, we performed **the Hausmann test** for the overall mortality data. By following different goodness of fit criteria including AIC, BIC and the Log-likelihood, we conclude that the fixed effects model was more adequate to analyze the effects of USAID per capita on this kind of mortality. The Web Table 6 shows this test.

Second, we change **the model specification**, measure the pure regression (without covariates), and omit some variables (Web Tables 7).

Third, to evaluate **the influence of the categorization**, we estimated the models by changing the thresholds using **dummy, tercile** and **quintile** approach (Web Table 8). Shifting the USAID disbursement per capita coverage from 4 categories to dummy variables serves to test our models in two key ways: (1st) to determine whether having the exposure variable in four categories while the other covariates are in binary (dummy) form is inflating the results and the program's impact on health outcomes; and (2nd) to assess whether modifying the thresholds of the categorical exposure variable would significantly change the observed outcomes. We also estimated the models using **continuous variables** (Web Table 9), and the results remain in the same direction as the main model.

Fourth, to assess the **external validity** of our estimates, we refitted the models using data from **all 204 countries and territories**. We also estimated the models excluding highly populous countries, such as China and India (see Web Table 10). Fifth, to evaluate the relevance of the **time dummies**, we tested alternative specifications of time variables (see Web Table 11).

Sixth, to examine **the stability of the results** under different model specifications, we fitted **Negative Binomial regression** models and compared their Akaike Information Criterion (AIC) and Bayesian Information Criterion (BIC) values with those of the Poisson models (see Web Table 12). Although the Negative Binomial model is a generalization of the Poisson model and accounts for overdispersion, it also requires the estimation of an additional dispersion parameter, making it a less parsimonious alternative. In our case, the Poisson model yielded lower AIC and BIC values compared to the Negative Binomial model, indicating a better balance between model fit and complexity. Therefore, we retained the Poisson specification..

Seventh, we conducted a series of **heterogeneity analyses** by stratifying the models according to **countries' income groups** (i.e., Low-Income, Lower-Middle-Income, and Upper-Middle-Income countries; see Web Table 13) and by disaggregating the results across a **larger number of age groups** (see Web Table 15). Stratified analyses by gender and by age group among women were also conducted (see Web Table 17-18). We also analyzed the heterogeneity between groups of countries with low and high levels of Human Development Index (HDI), Gross Domestic Product (GDP), and Gini Index (see Web Table 14). The classification of countries into low and high groups was based on quartiles of their distribution, even though there are predefined thresholds in the literature for what constitutes high or low levels (particularly for HDI and GDP). We chose this approach because, for example, if we were to use the standard HDI classification, most countries in our sample—already limited to low-, lower-middle-, and upper-middle-income countries—would fall into the low and medium HDI categories, reducing the analytical usefulness of such a grouping.

Eighth, we performed **falsification/placebo tests (negative controls)** to assess potential unexpected effects on either the exposure or outcome variables. Specifically, we used child mortality due to external causes (ICD-10 codes V01–Y89) as the outcome, as USAID interventions are not expected to influence this category nor include actions targeting its prevention (see Web Table 15).

Finally, to examine **internal aspects of USAID and the United States' role in relation to other global donors** in greater detail, we performed two additional analyses. The first focuses exclusively on USAID health-related funding and its association with mortality from specific disease categories (HIV/AIDS, malaria, and maternal health). Since USAID's financial allocation to health is substantially smaller than its total budget, we recalculated the per capita health-specific USAID distribution by quartiles and applied these cutoffs to total USAID funding (which includes both health and non-health areas). The results show that health-targeted spending has significantly stronger effects in reducing disease burdens related to HIV/AIDS, malaria, and maternal health, supporting both the robustness and the relevance of focusing on health investments (see Web Table 19).

The final sensitivity analysis explores **the weight of USAID within the global donor landscape**. We used OECD data and filtered for donations directed to low- and middle-income countries, as well as upper-middle-income countries. Then, we calculated the proportion of USAID funding relative to total aid from all donors and included this share as a fixed effect by country group. The results indicate a positive and significant association between USAID funding and reductions in disease burden, consistent with the main findings presented in the manuscript (see Web Table 20).

In other words, given that our results withstood the vast number of sensitivity tests performed, we conclude that the results and conclusions drawn in this study are robust and stable.

WEB TABLE 6. Hausman test between fixed effect and random effect Poisson models

	Mortality	
	**Fixed Effect**	**Random effect**
Number of observations	2,793	2,793
Number of countries	133	133
Log likelihood	-4,506,573.8	-4,508,513.9
Akaike information criterion (AIC)	9,013,182	9,017,066
Bayesian information criterion (BIC)	9,013,282	9,017,178
**Hausman test**	**χ^2=92.14; p-value= 0.000**	

WEB TABLE 7. Rate Ratios from the fixed effect Poisson models for the association between overall mortality rates and USAID per capita, for Omitted variable bias test, from the period 2001-21, in World

VARIABLES	Pure regression	Whitou time control	Without selected variables	Without selected variables	All variables
**USAID per capita**					
**Baseline (US$ 0.45)**	1	1	1	1	1
(US$ 0.00 to US$ 1.96)	[1.000,1.000]	[1.000,1.000]	[1.000,1.000]	[1.000,1.000]	[1.000,1.000]
**Low (US$ 2.88)**	0.947**	0.946***	0.943**	0.946**	0.946**

(US$ 1.97 to US$ 3.96)	[0.907,0.988]	[0.907,0.986]	[0.896,0.992]	[0.906,0.987]	[0.904,0.991]
**Intermediate (US$ 5.36)**	0.902***	0.914***	0.914***	0.914***	0.915***
(US$ 3.97 to US$ 7.09)	[0.844,0.964]	[0.859,0.972]	[0.854,0.978]	[0.861,0.970]	[0.858,0.975]
**High (US$ 20.45)**	0.846***	0.866***	0.853***	0.864***	0.863***
(US$ 7.10 or more)	[0.772,0.928]	[0.799,0.939]	[0.773,0.940]	[0.802,0.930]	[0.795,0.937]
**Control Variables**					
Gini Index		1.065**		1.067**	1.058***
		[1.015,1.118]		[1.011,1.125]	[1.015,1.102]
Primary Education (% population)		0.988	0.958*		0.983
		[0.949,1.029]	[0.913,1.005]		[0.944,1.024]
Education expenditure (% GDP)		0.935*	0.927**	0.934*	0.936**
		[0.874,1.001]	[0.863,0.997]	[0.871,1.002]	[0.876,1.000]
Piped water (% population)		0.950***	0.931**		0.968***
		[0.929,0.971]	[0.876,0.989]		[0.949,0.988]
Adequate sanitation (% population)		0.944		0.924	0.918***
		[0.857,1.041]		[0.832,1.027]	[0.864,0.975]
Nurse rate (per 1,000 population)		0.885***	0.902***	0.859***	0.886***
		[0.849,0.923]	[0.865,0.941]	[0.809,0.913]	[0.854,0.919]
Hospital bed rate (per 1,000 population)		0.981	0.993		0.985
		[0.958,1.005]	[0.965,1.022]		[0.961,1.009]
Health expenditure (% GDP)		0.96	0.934*		0.951*
		[0.909,1.013]	[0.871,1.000]		[0.899,1.007]
Millitary expenditure (% GDP)		1.077**		1.077**	1.076**
		[1.010,1.148]		[1.011,1.147]	[1.011,1.146]
**Time trend control**					
y2008			1.001	1.013	1.012
			[0.958,1.046]	[0.993,1.034]	[0.992,1.032]
y2015			0.956**	0.949***	0.955***
			[0.924,0.990]	[0.924,0.976]	[0.924,0.986]
y2021			1.100***		1.112***
			[1.061,1.140]		[1.062,1.163]
y2019					0.904***
					[0.877,0.932]
y2020					1.024
					[0.992,1.058]
**Number of observations**	2793	2793	2793	2793	2793
**Number of countries**	133	133	133	133	133

Source: Author's data analysis for 2,793 observations – 133 countries/territories in the World, over 20 years (from 2001 to 2021).
Note: Data are in Rate Ratio (RR) coefficients (95% CI) unless otherwise specified. The confidence intervals are in parentheses. Time shocks are controls for specific years of economic and health crisis (2008, 2015, 2019, 2020 and 2021). The symbols '***' , '**' and '*' denote significance at 1%, 5%, and 10% respectively.

WEB TABLE 8. Rate Ratios from the fixed effect Poisson models for the association between overall mortality rates with USAID per capita categorization (different types), from 2001-21, in the World.

VARIABLES	USAID per capita categorization			
	Dummy	Tercile	Quartile	Quintile
**USAID per capita**				
**1st dummy** (baseline - below median)	1			
	[1.000,1.000]			
2nd dummy (median and above)	0.915***			
	[0.878,0.954]			
**1st tercile (baseline)**		1		
		[1.000,1.000]		
2nd tercile		0.946**		
		[0.904,0.989]		
3rd tercile		0.894***		
		[0.839,0.954]		
**1st quartile (baseline)**			1	
			[1.000,1.000]	

VARIABLES	USAID per capita categorization			
	Dummy	Tercile	Quartile	Quintile
2nd quartile			0.946**	
			[0.904,0.991]	
3rd quartile			0.915***	
			[0.858,0.975]	
4th quartile			0.863***	
			[0.795,0.937]	
**1st quintile (baseline)**				1
				[1.000,1.000]
2nd quintile				0.946**
				[0.904,0.991]
3rd quintile				0.915***
				[0.858,0.976]
4th quintile				0.870***
				[0.795,0.951]
5th quintile				0.856***
				[0.785,0.933]
**Control Variables**				
Gini Index	1.058***	1.058***	1.058***	1.058***
	[1.014,1.103]	[1.015,1.103]	[1.015,1.102]	[1.015,1.102]
Primary Education (% population)	0.977	0.98	0.983	0.983
	[0.935,1.020]	[0.940,1.023]	[0.944,1.024]	[0.945,1.024]
Education expenditure (% GDP)	0.931**	0.936*	0.936**	0.935**
	[0.873,0.994]	[0.876,1.001]	[0.876,1.000]	[0.875,0.999]
Piped water (% population)	0.968***	0.969***	0.968***	0.968***
	[0.949,0.987]	[0.949,0.990]	[0.949,0.988]	[0.949,0.988]
Adequate sanitation (% population)	0.920***	0.919***	0.918***	0.918***
	[0.864,0.978]	[0.864,0.978]	[0.864,0.975]	[0.864,0.975]
Nurse rate (per 1,000 population)	0.886***	0.886***	0.886***	0.886***
	[0.854,0.918]	[0.854,0.918]	[0.854,0.919]	[0.855,0.919]
Hospital bed rate (per 1,000 population)	0.983	0.984	0.985	0.985
	[0.961,1.005]	[0.962,1.007]	[0.961,1.009]	[0.961,1.009]
Health expenditure (% GDP)	0.950*	0.951*	0.951*	0.951*
	[0.899,1.003]	[0.900,1.005]	[0.899,1.007]	[0.899,1.006]
Millitary expenditure (% GDP)	1.082**	1.080**	1.076**	1.077**
	[1.007,1.162]	[1.010,1.155]	[1.011,1.146]	[1.011,1.147]
**Time trend control**				
y2007	1.013	1.012	1.012	1.012
	[0.992,1.035]	[0.992,1.032]	[0.992,1.032]	[0.992,1.032]
y2015	0.954***	0.955***	0.955***	0.955***
	[0.923,0.986]	[0.925,0.986]	[0.924,0.986]	[0.924,0.986]
y2021	0.901***	0.901***	0.904***	0.905***
	[0.872,0.930]	[0.873,0.930]	[0.877,0.932]	[0.878,0.932]
y2019	1.019	1.021	1.024	1.024
	[0.988,1.052]	[0.989,1.054]	[0.992,1.058]	[0.992,1.058]
y2020	1.109***	1.107***	1.112***	1.112***
	[1.063,1.157]	[1.060,1.157]	[1.062,1.163]	[1.062,1.164]
**Number of observations**	2,793	2,793	2,793	2,793
**Number of countries**	133	133	133	133

Source: Author's data analysis for 2,793 observations – 133 countries/territories in the World, over 20 years (from 2001 to 2021).
Note: Data are in Rate Ratio (RR) coefficients (95% CI) unless otherwise specified. The confidence intervals are in parentheses. Time shocks are controls for specific years of economic and health crisis (2008, 2015, 2019, 2020 and 2021). The symbols '***' , '**' and '*' denote significance at 1%, 5%, and 10% respectively.

WEB TABLE 9. Rate Ratios from the fixed effect Poisson models for the association between overall mortality rates with USAID per capita using continuous variables, from 2001-21 in selected countries/territories.

VARIABLES	Pure regression	All variables

**USAID per capita**	0.421***	0.776**
	[0.268,0.662]	[0.632,0.954]
**Control Variables**		
GDP per capita PPP	1.000***	1.000***
	[1.000,1.000]	[1.000,1.000]
Gini Index		1.357*
		[0.978,1.882]
Employers (%)		0.989**
		[0.980,0.998]
Literacy rate (%)		0.998
		[0.995,1.001]
Piped water (% population)		0.996***
		[0.993,0.998]
Adequate sanitation (% population)		0.995***
		[0.993,0.997]
Health expenditure (% GDP)		1.010*
		[1.000,1.019]
Universal Health coverage (%)		0.989***
		[0.987,0.991]
Years		1.005***
		[1.003,1.007]
**Number of observations**	2,793	2,793
**Number of countries**	133	133

Source: Author's data analysis for 2,793 observations – 133 countries/territories in the World, over 20 years (from 2001 to 2021).
Note: Data are in Rate Ratio (RR) coefficients (95% CI) unless otherwise specified. The confidence intervals are in parentheses. Time shocks are controls for specific years of economic and health crisis (2008, 2015, 2019, 2020 and 2021). The symbols '***' , '**' and '*' denote significance at 1%, 5%, and 10% respectively.

WEB TABLE 10. Rate Ratios from the fixed effect Poisson models for the association between overall mortality rates with USAID per capita using categorical variables, from 2001-21, in all countries/territories.

VARIABLES	Selected 133 countries		All countries and territories	
	Overall	without China and India	Pure regression	All variables
**USAID per capita**				
**Baseline (US$ 0.45)**	1	1	1	1
(US$ 0.00 to US$ 1.96)	[1.000,1.000]	[1.000,1.000]	[1.000,1.000]	[1.000,1.000]
**Low (US$ 2.88)**	0.942**	0.944**	0.948**	0.946**
(US$ 1.97 to US$ 3.96)	[0.894,0.992]	[0.896,0.994]	[0.908,0.990]	[0.907,0.987]
**Intermediate (US$ 5.36)**	0.911***	0.909***	0.905***	0.910***
(US$ 3.97 to US$ 7.09)	[0.850,0.975]	[0.854,0.968]	[0.847,0.966]	[0.860,0.963]
**High (US$ 20.45)**	0.851***	0.847***	0.850***	0.853***
(US$ 7.10 or more)	[0.780,0.930]	[0.782,0.919]	[0.776,0.932]	[0.790,0.921]
**Control Variables**				
Gini Index	1.059***	1.052*		0.967
	[1.015,1.105]	[0.996,1.110]		[0.924,1.013]
Primary Education (% population)	0.980	0.968*		1.02
	[0.941,1.021]	[0.937,1.001]		[0.971,1.071]
Education expenditure (% GDP)	0.930**	0.922**		0.942***
	[0.869,0.996]	[0.860,0.988]		[0.904,0.982]
Piped water (% population)	0.964***	1.014		0.994
	[0.944,0.983]	[0.965,1.065]		[0.948,1.043]
Adequate sanitation (% population)	0.917***	0.926*		0.878***
	[0.862,0.976]	[0.852,1.006]		[0.830,0.930]
Nurse rate (per 1,000 population)	0.881***	0.939***		1
	[0.850,0.912]	[0.901,0.980]		[0.962,1.039]
Hospital bed rate (per 1,000 population)	0.997	0.999		0.849***
	[0.973,1.021]	[0.933,1.069]		[0.809,0.891]
Health expenditure (% GDP)	0.942*	1.003		0.998
	[0.882,1.007]	[0.972,1.034]		[0.957,1.040]
Millitary expenditure (% GDP)	1.080**	1.080**		1.074***
	[1.011,1.153]	[1.012,1.153]		[1.023,1.129]
**Time trend control**				
y2008	1.012	1.034***		1.011
	[0.992,1.032]	[1.019,1.050]		[0.974,1.048]
y2015	0.955***	0.938***		0.927***
	[0.924,0.986]	[0.918,0.959]		[0.898,0.958]
y2021	0.904***	1.026*		0.893***
	[0.877,0.932]	[0.998,1.054]		[0.864,0.923]
y2019	1.024	1.136***		0.988
	[0.992,1.058]	[1.100,1.174]		[0.967,1.008]
y2020	1.112***	1.034***		1.071***
	[1.062,1.163]	[1.019,1.050]		[1.037,1.105]
**Number of observations**	2,793	2,751	3,990	3,990
**Number of countries**	133	131	190	190

Source: Author's data analysis for 3,990 observations – 190 countries/territories in the World, over 21 years (from 2001 to 2021).
Note: Data are in Rate Ratio (RR) coefficients (95% CI) unless otherwise specified. The confidence intervals are in parentheses. Time shocks are controls for specific years of economic and health crisis (2008, 2015, 2019, 2020 and 2021). The symbols '***' , '**' and '*' denote significance at 1%, 5%, and 10% respectively

WEB TABLE 11. Rate Ratios from the fixed effect Poisson models for the association between overall mortality rates and USAID per capita using categorical variables, with different time shocks control, from 2001-21 in selected countries/territories.

VARIABLES	OVERALL MORTALITY RATE BY DIFFERENT TIME SHOCK CONTROL					
	**No time Control**	**First 5 years** 2001 to 2005	**Second 5 years** 2006 to 2010	**Thrid 5 years** 2011 to 2015	**Last 5 years** 2016 to 2020	**All Period** 2002 to 2021
**USAID per capita**						
**Baseline (US$ 0.45)**	1	1	1	1	1	1
(US$ 0.00 to US$ 1.96)	[1.000,1.000]	[1.000,1.000]	[1.000,1.000]	[1.000,1.000]	[1.000,1.000]	[1.000,1.000]
**Low (US$ 2.88)**	0.946***	0.969*	0.946***	0.947**	0.960***	0.984*
(US$ 1.97 to US$ 3.96)	[0.907,0.986]	[0.936,1.004]	[0.907,0.986]	[0.905,0.991]	[0.933,0.988]	[0.963,0.999]
**Intermediate (US$ 5.36)**	0.914***	0.941**	0.914***	0.917***	0.927***	0.962**
(US$ 3.97 to US$ 7.09)	[0.859,0.972]	[0.898,0.987]	[0.859,0.972]	[0.864,0.974]	[0.883,0.974]	[0.927,0.999]
**High (US$ 20.45)**	0.866***	0.906***	0.867***	0.866***	0.897***	0.938**
(US$ 7.10 or more)	[0.799,0.939]	[0.857,0.957]	[0.801,0.939]	[0.800,0.938]	[0.842,0.956]	[0.890,0.988]
**Control Variables**						
Gini Index	1.065**	1.067**	1.066**	1.061**	1.049**	1.04
	[1.015,1.118]	[1.007,1.131]	[1.013,1.122]	[1.010,1.115]	[1.001,1.099]	[0.990,1.092]
Primary Education (% population)	0.988	1.017	0.989	0.992	0.999	1.037
	[0.949,1.029]	[0.975,1.061]	[0.950,1.029]	[0.952,1.033]	[0.960,1.039]	[0.987,1.089]
Education expenditure (% GDP)	0.935*	0.959	0.935*	0.941*	0.954	0.992
	[0.874,1.001]	[0.907,1.014]	[0.874,1.001]	[0.882,1.003]	[0.896,1.016]	[0.949,1.037]
Piped water (% population)	0.950***	0.977	0.952***	0.964**	0.977	1.044***
	[0.929,0.971]	[0.947,1.008]	[0.927,0.977]	[0.934,0.994]	[0.950,1.005]	[1.011,1.078]
Adequate sanitation (% population)	0.944	0.982	0.945	0.939	0.931*	0.941***
	[0.857,1.041]	[0.920,1.047]	[0.855,1.045]	[0.856,1.030]	[0.864,1.003]	[0.906,0.978]
Nurse rate (per 1,000 population)	0.885***	0.908***	0.883***	0.891***	0.913***	0.954**
	[0.849,0.923]	[0.862,0.956]	[0.839,0.931]	[0.857,0.926]	[0.868,0.961]	[0.912,0.998]
Hospital bed rate (per 1,000 population)	0.981	1.004	0.982	0.989	0.964***	0.996
	[0.958,1.005]	[0.984,1.024]	[0.961,1.004]	[0.963,1.015]	[0.940,0.990]	[0.973,1.020]
Health expenditure (% GDP)	0.96	0.961	0.96	0.952*	0.971	0.954*
	[0.909,1.013]	[0.908,1.018]	[0.908,1.015]	[0.898,1.008]	[0.933,1.010]	[0.905,1.006]
Millitary expenditure (% GDP)	1.077**	1.050*	1.077**	1.073**	1.062**	1.03
	[1.010,1.148]	[0.995,1.108]	[1.011,1.148]	[1.009,1.141]	[1.002,1.126]	[0.988,1.075]
**Time trend control**						
y2001		1.148***				
		[1.115,1.183]				
y2002		1.137***				0.990***
		[1.101,1.175]				[0.983,0.998]
y2003		1.139***				0.987**
		[1.105,1.175]				[0.978,0.997]
y2004		1.125***				0.975***
		[1.093,1.157]				[0.962,0.988]
y2005		1.098***				0.952***
		[1.067,1.129]				[0.944,0.961]
y2006			1.013			0.923***
			[0.963,1.066]			[0.899,0.948]
y2007			1.008			0.919***
			[0.972,1.045]			[0.898,0.941]
y2008			0.996			0.909***
			[0.963,1.031]			[0.887,0.931]
y2009			0.987			0.879***
			[0.956,1.019]			[0.858,0.902]
y2010			0.993			0.872***
			[0.972,1.015]			[0.848,0.896]
y2011				0.982		0.855***
				[0.943,1.023]		[0.829,0.883]
y2012				0.969		0.829***
				[0.932,1.008]		[0.801,0.858]
y2013				0.965*		0.821***
				[0.930,1.001]		[0.788,0.854]
y2014				0.960*		0.817***
				[0.917,1.006]		[0.782,0.853]
y2015				0.943***		0.802***
				[0.907,0.981]		[0.769,0.836]
y2016					0.915***	0.789***
					[0.892,0.939]	[0.758,0.821]

VARIABLES	OVERALL MORTALITY RATE BY DIFFERENT TIME SHOCK CONTROL					
	**No time Control**	**First 5 years** 2001 to 2005	**Second 5 years** 2006 to 2010	**Thrid 5 years** 2011 to 2015	**Last 5 years** 2016 to 2020	**All Period** 2002 to 2021
y2017					0.898***	0.775***
					[0.876,0.919]	[0.744,0.807]
y2018					0.878***	0.766***
					[0.851,0.906]	[0.735,0.799]
y2019					0.866***	0.755***
					[0.840,0.894]	[0.724,0.788]
y2020					0.977**	0.850***
					[0.957,0.998]	[0.806,0.896]
y2021						0.920**
						[0.862,0.982]
**Number of observations**	2793	2793	2793	2793	2793	2793
**Number of countries**	133	133	133	133	133	133

Source: Author's data analysis for 2,793 observations – 133 countries/territories in the World, over 20 years (from 2001 to 2021).
Note: Data are in Rate Ratio (RR) coefficients (95% CI) unless otherwise specified. The confidence intervals are in parentheses. The symbols '***' , '**' and '*' denote significance at 1%, 5%, and 10% respectively

WEB TABLE 12. Rate Ratios for the association between overall mortality rates with USAID per capita, using Negative Binomial models; from 2001-21 in selected countries/territories.

VARIABLES	Negative Binomial		Poisson	
	**Pure regression**	**All variables**	**Pure regression**	**All variables**
**USAID per capita**				
**Baseline (US$ 0.45)**	1	1	1	1
(US$ 0.00 to US$ 1.96)	[1.000,1.000]	[1.000,1.000]	[1.000,1.000]	[1.000,1.000]
**Low (US$ 2.88)**	0.979*	0.951***	0.947**	0.942**
(US$ 1.97 to US$ 3.96)	[0.958,1.001]	[0.936,0.966]	[0.907,0.988]	[0.894,0.992]
**Intermediate (US$ 5.36)**	0.958***	0.929***	0.902***	0.911***
(US$ 3.97 to US$ 7.09)	[0.932,0.985]	[0.911,0.947]	[0.844,0.964]	[0.850,0.975]
**High (US$ 20.45)**	0.933***	0.890***	0.846***	0.851***
(US$ 7.10 or more)	[0.904,0.962]	[0.870,0.910]	[0.772,0.928]	[0.780,0.930]
**Control Variables**				
GDP per capita PPP		1		1
		[1.000,1.000]		[1.000,1.000]
Gini Index		1.067***		1.059***
		[1.054,1.080]		[1.015,1.105]
Primary Education (% population)		0.994		0.980
		[0.975,1.013]		[0.941,1.021]
Education expenditure (% GDP)		0.951***		0.930**
		[0.934,0.969]		[0.869,0.996]
Piped water (% population)		0.955***		0.964***
		[0.942,0.969]		[0.944,0.983]
Adequate sanitation (% population)		0.926***		0.917***
		[0.914,0.939]		[0.862,0.976]
Nurse rate (per 1,000 population)		0.887***		0.881***
		[0.875,0.898]		[0.850,0.912]
Hospital bed rate (per 1,000 population)		0.989*		0.997
		[0.977,1.001]		[0.973,1.021]
Health expenditure (% GDP)		0.949***		0.942*
		[0.937,0.960]		[0.882,1.007]
Millitary expenditure (% GDP)		1.055***		1.080**
		[1.038,1.073]		[1.011,1.153]
**Time trend control**				
y2007		1.007		1.012
		[0.992,1.022]		[0.992,1.032]
y2015		0.959***		0.955***
		[0.945,0.973]		[0.924,0.986]
y2021		0.911***		0.904***
		[0.897,0.925]		[0.877,0.932]
y2019		1.021***		1.024
		[1.006,1.037]		[0.992,1.058]
y2020		1.099***		1.112***
		[1.083,1.115]		[1.062,1.163]
**AIC**	60705.14	58915.94	1.62e+07	9,520,461
**BIC**	60728.88	59022.77	1.62e+07	9,520,556
**Number of observations**	2793	2793	2793	2793
**Number of countries**	133	133	133	133

Source: Author's data analysis for 2,793 observations – 133 countries/territories in the World, over 20 years (from 2001 to 2021).
Note: Data are in Rate Ratio (RR) coefficients (95% CI) unless otherwise specified. The confidence intervals are in parentheses. Time shocks are controls for specific years of economic and health crisis (2008, 2015, 2019, 2020 and 2021). The symbols '***' , '**' and '*' denote significance at 1%, 5%, and 10% respectively

WEB TABLE 13. Rate Ratios from the fixed effect Poisson models for the association between overall mortality rates and USAID per capita using categorical variables, dividing the models by countries' income groups (heterogeneity analyses); from 2001-21 in selected countries/territories.

VARIABLES	LIC Low-Income	LMIC Lower-Middle income	UMIC Upper-Middle income	WORLD
**USAID per capita**				
**Baseline (US$ 0.45)**	1	1	1	1
(US$ 0.00 to US$ 1.96)	[1.000,1.000]	[1.000,1.000]	[1.000,1.000]	[1.000,1.000]
**Low (US$ 2.88)**	0.942***	0.929***	0.946***	0.946***
(US$ 1.97 to US$ 3.96)	[0.941,0.943]	[0.929,0.930]	[0.946,0.947]	[0.946,0.947]
**Intermediate (US$ 5.36)**	0.895***	0.921***	0.921***	0.915***
(US$ 3.97 to US$ 7.09)	[0.895,0.896]	[0.920,0.921]	[0.921,0.921]	[0.915,0.915]
**High (US$ 20.45)**	0.834***	0.864***	0.892***	0.863***
(US$ 7.10 or more)	[0.834,0.835]	[0.863,0.864]	[0.892,0.893]	[0.863,0.863]
**Control Variables**				
Gini Index	1.072***	1.002***	1.059***	1.058***
	[1.071,1.073]	[1.002,1.003]	[1.058,1.059]	[1.058,1.058]
Primary Education (% population)	0.941***	0.965***	0.979***	0.983***
	[0.940,0.942]	[0.964,0.966]	[0.978,0.979]	[0.983,0.983]
Education expenditure (% GDP)	0.929***	0.958***	0.932***	0.936***
	[0.928,0.930]	[0.958,0.959]	[0.932,0.933]	[0.935,0.936]
Piped water (% population)		0.975***	0.966***	0.968***
		[0.974,0.975]	[0.966,0.966]	[0.968,0.968]
Adequate sanitation (% population)	0.896***	0.986***	0.920***	0.918***
	[0.893,0.899]	[0.986,0.986]	[0.919,0.920]	[0.918,0.918]
Nurse rate (per 1,000 population)	0.828***	0.942***	0.887***	0.886***
	[0.827,0.830]	[0.942,0.943]	[0.887,0.887]	[0.886,0.886]
Hospital bed rate (per 1,000 population)	0.969***	0.996***	0.987***	0.985***
	[0.969,0.970]	[0.996,0.997]	[0.987,0.987]	[0.985,0.985]
Health expenditure (% GDP)	0.951***	1.042***	0.953***	0.951***
	[0.950,0.951]	[1.042,1.043]	[0.953,0.953]	[0.951,0.951]
Millitary expenditure (% GDP)	1.151***	1.037***	1.039***	1.076***
	[1.150,1.152]	[1.036,1.037]	[1.038,1.039]	[1.076,1.077]
**Time trend control**				
y2007	1.014***	1.027***	1.010***	1.012***
	[1.013,1.015]	[1.026,1.027]	[1.009,1.010]	[1.011,1.012]
y2015	0.950***	0.954***	0.957***	0.955***
	[0.949,0.951]	[0.954,0.955]	[0.957,0.957]	[0.954,0.955]
y2021	0.875***	0.898***	0.907***	0.904***
	[0.874,0.876]	[0.897,0.898]	[0.907,0.908]	[0.904,0.905]
y2019	1.028***	1.001***	1.023***	1.024***
	[1.027,1.029]	[1.001,1.002]	[1.023,1.024]	[1.024,1.025]
y2020	1.154***	1.094***	1.105***	1.112***
	[1.154,1.155]	[1.093,1.094]	[1.104,1.105]	[1.111,1.112]
**Number of observations**	504	1176	1,113	2793
**Number of countries**	24	56	53	133

Source: Author's data analysis for 2,793 observations – 133 countries/territories in the World, over 20 years (from 2001 to 2021).
Note: Data are in Rate Ratio (RR) coefficients (95% CI) unless otherwise specified. The confidence intervals are in parentheses. Time shocks are controls for specific years of economic and health crisis (2008, 2015, 2019, 2020 and 2021). The symbols '***' , '**' and '*' denote significance at 1%, 5%, and 10% respectively

WEB TABLE 14. Rate Ratios from the fixed effect Poisson models for the association between overall mortality rates and USAID per capita per year, for groups of countries according to IHD, GDP and Gini levels

VARIABLES	HDI		GDP pc PPP		Gini index	
	Low	High	Low	High	High	Low
**USAID per capita**						
**Baseline (US$ 0.45)**	1	1	1	1	1	1
(US$ 0.00 to US$ 1.96)	[1.000,1.000]	[1.000,1.000]	[1.000,1.000]	[1.000,1.000]	[1.000,1.000]	[1.000,1.000]
**Low (US$ 2.88)**	0.888***	0.986	0.893***	0.95	0.929**	0.979
(US$ 1.97 to US$ 3.96)	[0.843,0.936]	[0.946,1.029]	[0.861,0.926]	[0.891,1.013]	[0.872,0.991]	[0.948,1.011]
**Intermediate (US$ 5.36)**	0.875***	0.931*	0.836***	0.945*	0.859***	0.951
(US$ 3.97 to US$ 7.09)	[0.820,0.934]	[0.866,1.001]	[0.789,0.886]	[0.885,1.010]	[0.801,0.922]	[0.886,1.020]
**High (US$ 20.45)**	0.796***	0.843***	0.724***	0.961	0.742***	0.911*
(US$ 7.10 or more)	[0.734,0.864]	[0.790,0.899]	[0.680,0.771]	[0.890,1.039]	[0.693,0.795]	[0.820,1.011]
**Control Variables**						
Gini Index	1.056**	1.015	1.028	1.076***		
	[1.005,1.109]	[0.993,1.037]	[0.952,1.110]	[1.029,1.125]		
Primary Education (% population)	0.960***	0.933**	0.958***	0.971	1	0.988
	[0.933,0.989]	[0.881,0.988]	[0.928,0.989]	[0.924,1.022]	[0.924,1.083]	[0.943,1.036]
Education expenditure (% GDP)	0.931**	1.012	0.939*	0.936	0.984	0.925**
	[0.878,0.988]	[0.971,1.056]	[0.874,1.008]	[0.861,1.017]	[0.948,1.022]	[0.858,0.998]
Piped water (% population)		0.969***	1.073**	0.955***	0.969***	0.890***
		[0.951,0.987]	[1.012,1.138]	[0.939,0.972]	[0.952,0.987]	[0.839,0.944]
Adequate sanitation (% population)	0.857***	0.971**	0.851***	0.925**	0.872***	1.011
	[0.828,0.888]	[0.948,0.994]	[0.816,0.888]	[0.872,0.982]	[0.858,0.886]	[0.949,1.077]
Nurse rate (per 1,000 population)	0.881***	0.963***	0.938	0.879***	0.941***	1.022
	[0.849,0.914]	[0.951,0.975]	[0.865,1.016]	[0.848,0.912]	[0.926,0.956]	[0.971,1.076]
Hospital bed rate (per 1,000 population)	0.969	1.066***	0.942**	0.996	0.910**	1.025***
	[0.921,1.019]	[1.024,1.110]	[0.889,1.000]	[0.978,1.015]	[0.836,0.989]	[1.019,1.032]
Health expenditure (% GDP)	0.972	1.01	0.988	0.927**	1	1.007
	[0.916,1.032]	[0.971,1.051]	[0.940,1.039]	[0.863,0.996]	[0.947,1.055]	[0.963,1.053]
Millitary expenditure (% GDP)	1.144**	1.027	1.117**	1.045**	0.996	1.185*
	[1.028,1.273]	[0.990,1.065]	[1.024,1.218]	[1.004,1.088]	[0.912,1.087]	[0.999,1.404]
Time trend control	Yes	Yes	Yes	Yes	Yes	Yes
**Number of observations**	629	599	669	2,120	654	652
**Number of countries**	34	37	40	110	66	52

Source: Author's data analysis for 2,793 observations – 133 countries/territories in the World, over 20 years (from 2001 to 2021).
Note: HDI = Human Development Index, GDP pc PPP = Gross Domestic Product (GDP) per capita (pc), adjusted for Purchasing Power Parity (PPP). Data are in Rate Ratio (RR) coefficients (95% CI) unless otherwise specified. The confidence intervals are in parentheses. Time shocks are controls for specific years of economic and health crisis (2008, 2015, 2019, 2020 and 2021). The symbols '***' , '**' and '*' denote significance at 1%, 5%, and 10% respectively

WEB TABLE 15. Rate Ratios from the fixed effect Poisson models for the association between overall mortality rates and USAID per capita using categorical variables, by different causes of deaths (negative control); from 2001-21, in selected countries/territories.

VARIABLES	Tuberculosis	HIV-AIDS	Maternal mortality	Lower respiratory infections	Nutritional Deficiencies	Diarrhoeal diseases	Malaria	Neglected tropical diseases	Injuries
**USAID per capita**									
**Baseline (US$ 0.45)**	1	1	1	1	1	1	1	1	1
(US$ 0.00 to US$ 1.96)	[1.000,1.000]	[1.000,1.000]	[1.000,1.000]	[1.000,1.000]	[1.000,1.000]	[1.000,1.000]	[1.000,1.000]	[1.000,1.000]	[1.000,1.000]
**Low (US$ 2.88)**	0.856**	0.821**	0.926**	0.852**	0.825**	0.732***	0.761***	0.765***	0.988
(US$ 1.97 to US$ 3.96)	[0.757,0.969]	[0.705,0.956]	[0.862,0.994]	[0.742,0.977]	[0.697,0.978]	[0.585,0.916]	[0.693,0.835]	[0.697,0.840]	[0.901,1.083]
**Intermediate (US$ 5.36)**	0.838**	0.568***	0.899**	0.808***	0.736**	0.701***	0.710***	0.711***	1.016
(US$ 3.97 to US$ 7.09)	[0.723,0.972]	[0.486,0.663]	[0.818,0.987]	[0.699,0.933]	[0.582,0.930]	[0.544,0.902]	[0.580,0.870]	[0.579,0.873]	[0.882,1.171]
**High (US$ 20.45)**	0.710***	0.348***	0.762***	0.689***	0.570***	0.545***	0.490***	0.497***	1.082
(US$ 7.10 or more)	[0.599,0.841]	[0.290,0.419]	[0.677,0.857]	[0.573,0.828]	[0.441,0.736]	[0.417,0.713]	[0.394,0.610]	[0.400,0.618]	[0.844,1.388]
**Control variables**	Yes	Yes	Yes	Yes	Yes	Yes	Yes	Yes	Yes
Years binaries	Yes	Yes	Yes	Yes	Yes	Yes	Yes	Yes	Yes
**Number of observations**	2,793	2,793	2,793	2,793	2,793	2,793	2,079	2,793	2,793
**Number of countries**	133	133	133	133	133	133	133	133	133

Source: Author's data analysis for 2,793 observations – 133 countries/territories in the World, over 20 years (from 2001 to 2021).
Note: Data are in Rate Ratio (RR) coefficients (95% CI) unless otherwise specified. The confidence intervals are in parentheses. Time shocks are controls for specific years of economic and health crisis (2008, 2015, 2019, 2020 and 2021). The symbols '***' , '**' and '*' denote significance at 1%, 5%, and 10% respectively

WEB TABLE 16. Rate Ratios from the fixed effect Poisson models for the association between mortality rates and USAID per capita using categorical variables, by different age groups; from 2001-21, in selected countries/territories.

VARIABLES	Neonatal	Infancy	Preschool	Child	School-age	Adult	Elderly	Overall
	0 to 28 days	0 to 1 year	2 to 4 years	Under 5 years	5 to 9 years	10 to 54 years	55+ years	
**USAID per capita**								
**Baseline (US$ 0.45)**	1	1	1	1	1	1	1	1
(US$ 0.00 to US$ 1.96)	[1.000,1.000]	[1.000,1.000]	[1.000,1.000]	[1.000,1.000]	[1.000,1.000]	[1.000,1.000]	[1.000,1.000]	[1.000,1.000]
**Low (US$ 2.88)**	0.945**	0.919**	0.816***	0.882***	0.922**	0.946	0.968*	0.946**
(US$ 1.97 to US$ 3.96)	[0.901,0.990]	[0.860,0.982]	[0.731,0.912]	[0.811,0.960]	[0.865,0.983]	[0.879,1.018]	[0.934,1.003]	[0.904,0.991]
**Intermediate (US$ 5.36)**	0.916*	0.854**	0.747***	0.815***	0.900**	0.854***	0.958*	0.915***
(US$ 3.97 to US$ 7.09)	[0.833,1.007]	[0.751,0.972]	[0.626,0.892]	[0.707,0.940]	[0.814,0.997]	[0.766,0.952]	[0.915,1.002]	[0.858,0.975]
**High (US$ 20.45)**	0.879*	0.775***	0.600***	0.713***	0.842**	0.745***	0.949*	0.863***
(US$ 7.10 or more)	[0.761,1.015]	[0.661,0.907]	[0.491,0.732]	[0.604,0.841]	[0.734,0.966]	[0.640,0.868]	[0.892,1.009]	[0.795,0.937]
**Control Variables**								
Gini Index	1.097**	1.085*	1.029	1.080*	0.945	1.036	1.060***	1.058***
	[1.008,1.195]	[0.998,1.181]	[0.945,1.122]	[0.998,1.169]	[0.878,1.017]	[0.961,1.116]	[1.022,1.099]	[1.015,1.102]
Primary Education (% population)	0.972	0.952	0.945	0.953	0.98	0.971	1.011	0.983
	[0.913,1.036]	[0.884,1.024]	[0.852,1.049]	[0.884,1.027]	[0.901,1.065]	[0.890,1.059]	[0.965,1.059]	[0.944,1.024]
Education expenditure (% GDP)	0.943***	0.927***	0.863***	0.905***	0.877***	0.879*	0.973	0.936**
	[0.906,0.982]	[0.884,0.973]	[0.799,0.932]	[0.856,0.957]	[0.812,0.947]	[0.761,1.014]	[0.934,1.013]	[0.876,1.000]
Piped water (% population)	0.861***	0.842***	0.686***	0.817***	0.798***	0.947	0.981***	0.968***
	[0.815,0.908]	[0.801,0.886]	[0.634,0.743]	[0.784,0.851]	[0.728,0.874]	[0.877,1.022]	[0.968,0.994]	[0.949,0.988]
Adequate sanitation (% population)	0.888	0.842**	0.806**	0.841**	0.882**	0.953	0.938***	0.918***
	[0.767,1.029]	[0.721,0.983]	[0.677,0.960]	[0.718,0.985]	[0.796,0.977]	[0.851,1.067]	[0.904,0.972]	[0.864,0.975]
Nurse rate (per 1,000 population)	0.860***	0.848***	0.744***	0.821***	0.784***	0.887***	0.926***	0.886***
	[0.783,0.946]	[0.775,0.927]	[0.685,0.809]	[0.756,0.892]	[0.743,0.827]	[0.844,0.933]	[0.905,0.947]	[0.854,0.919]
Hospital bed rate (per 1,000 population)	0.987	0.983	0.959	0.978	1.062**	1.011	0.969***	0.985
	[0.935,1.042]	[0.929,1.041]	[0.903,1.019]	[0.924,1.034]	[1.004,1.124]	[0.972,1.051]	[0.953,0.985]	[0.961,1.009]
Health expenditure (% GDP)	0.969	0.968	0.942	0.96	0.952*	1.002	0.942*	0.951*
	[0.918,1.022]	[0.912,1.028]	[0.870,1.018]	[0.901,1.022]	[0.901,1.006]	[0.956,1.049]	[0.885,1.002]	[0.899,1.007]
Millitary expenditure (% GDP)	1.106***	1.137***	1.191**	1.147***	1.148***	1.086*	1.026	1.076**
	[1.027,1.192]	[1.055,1.227]	[1.027,1.381]	[1.048,1.254]	[1.037,1.271]	[0.997,1.182]	[0.987,1.067]	[1.011,1.146]
**Time trend control**								
y2007	1.030***	1.029**	1.011	1.027*	0.996	1.019	0.995	1.012
	[1.008,1.051]	[1.004,1.055]	[0.972,1.051]	[0.999,1.056]	[0.939,1.057]	[0.979,1.060]	[0.967,1.023]	[0.992,1.032]
y2015	0.932***	0.905***	0.822***	0.882***	0.880***	0.938***	0.976***	0.955***
	[0.885,0.981]	[0.855,0.958]	[0.780,0.867]	[0.833,0.934]	[0.843,0.919]	[0.909,0.968]	[0.959,0.992]	[0.924,0.986]
y2021	0.806***	0.769***	0.681***	0.744***	0.725***	0.879***	0.963**	0.904***
	[0.769,0.845]	[0.730,0.809]	[0.623,0.745]	[0.705,0.785]	[0.678,0.776]	[0.843,0.915]	[0.933,0.994]	[0.877,0.932]

VARIABLES	Neonatal<br>0 to 28 days	Infancy<br>0 to 1 year	Preschool<br>2 to 4 years	Child<br>Under 5 years	School-age<br>5 to 9 years	Adult<br>10 to 54 years	Elderly<br>55+ years	Overall
y2019	0.798***	0.749***	0.673***	0.724***	0.697***	0.969	1.079***	1.024
	[0.741,0.861]	[0.693,0.809]	[0.612,0.741]	[0.673,0.778]	[0.651,0.745]	[0.931,1.008]	[1.036,1.124]	[0.992,1.058]
y2020	0.770***	0.718***	0.637***	0.692***	0.672***	1.052*	1.165***	1.112***
	[0.715,0.829]	[0.666,0.774]	[0.585,0.695]	[0.645,0.743]	[0.625,0.722]	[0.997,1.111]	[1.080,1.257]	[1.062,1.163]
**Number of observations**	2793	2793	2793	2793	2793	2793	2793	2793
**Number of countries**	133	133	133	133	133	133	133	133

Source: Author's data analysis for 2,793 observations – 133 countries/territories in the World, over 21 years (from 2001 to 2021).
Note: Data are in Rate Ratio (RR) coefficients (95% CI) unless otherwise specified. The confidence intervals are in parentheses. Time shocks are controls for specific years of economic and health crisis (2008, 2015, 2019, 2020 and 2021). The symbols '***' , '**' and '*' denote significance at 1%, 5%, and 10% respectively

WEB TABLE 17. Rate Ratios from the fixed effect Poisson models for the association between mortality rates and USAID per capita using categorical variables, by sex; from 2001-21, in selected countries/territories.

VARIABLES	Female		Male	
	Pure regression	All variables	Pure regression	All variables
**USAID per capita**				
**Baseline (US$ 0.45)**	1	1	1	1
(US$ 0.00 to US$ 1.96)	[1.000,1.000]	[1.000,1.000]	[1.000,1.000]	[1.000,1.000]
**Low (US$ 2.88)**	0.935*	0.921**	0.951	0.931**
(US$ 1.97 to US$ 3.96)	[0.872,1.002]	[0.856,0.992]	[0.894,1.012]	[0.867,0.999]
**Intermediate (US$ 5.36)**	0.859***	0.853***	0.896***	0.883***
(US$ 3.97 to US$ 7.09)	[0.781,0.945]	[0.779,0.934]	[0.826,0.972]	[0.813,0.960]
**High (US$ 20.45)**	0.755***	0.741***	0.825***	0.801***
(US$ 7.10 or more)	[0.659,0.865]	[0.651,0.844]	[0.736,0.924]	[0.713,0.899]
**Control Variables**				
Gini Index		0.989		0.989
		[0.943,1.037]		[0.929,1.053]
Primary Education (% population)		1.026		1.026
		[0.947,1.112]		[0.949,1.109]
Education expenditure (% GDP)		0.956**		0.933**
		[0.915,0.998]		[0.876,0.994]
Piped water (% population)		1		0.986
		[0.979,1.022]		[0.945,1.029]
Adequate sanitation (% population)		1.006		1.044*
		[0.966,1.047]		[0.997,1.092]
Nurse rate (per 1,000 population)		0.992		1.008
		[0.953,1.032]		[0.966,1.052]
Hospital bed rate (per 1,000 population)		1.013		1.013
		[0.976,1.051]		[0.983,1.044]
Health expenditure (% GDP)		1.044**		1.014
		[1.009,1.082]		[0.992,1.036]
Millitary expenditure (% GDP)		1.058		1.047
		[0.964,1.161]		[0.965,1.136]
**Time trend control**				
y2007		1.011		1.001
		[0.993,1.029]		[0.974,1.030]
y2015		0.951***		0.958***
		[0.923,0.980]		[0.932,0.984]
y2020		1.057		1.084**
		[0.986,1.132]		[1.005,1.170]
y2021		1.087***		1.136***
		[1.024,1.154]		[1.065,1.213]
**Number of observations**	2376	2376	2376	2376
**Number of countries**	133	133	133	133

Source: Author's data analysis for 2,793 observations – 133 countries/territories in the World, over 21 years (from 2001 to 2021).
Note: Data are in Rate Ratio (RR) coefficients (95% CI) unless otherwise specified. The confidence intervals are in parentheses. Time shocks are controls for specific years of economic and health crisis (2008, 2015, 2019, 2020 and 2021). The symbols '***' , '**' and '*' denote significance at 1%, 5%, and 10% respectively

WEB TABLE 18. Rate Ratios from the fixed effect Poisson models for the association between mortality rates and USAID per capita using categorical variables, by female age groups; from 2001-21, in selected countries/territories.

VARIABLES	Children and adolescents	Youth and young adults	Young to middle-aged adults	Middle-aged adults	Elderly	Overall
	0 to 14 years	15 to 29 years	30 to 44 years	45 to 59 years	60+ years	(Female)
**USAID per capita**						
**Baseline (US$ 0.45)**	1	1	1	1	1	1
(US$ 0.00 to US$ 1.96)	[1.000,1.000]	[1.000,1.000]	[1.000,1.000]	[1.000,1.000]	[1.000,1.000]	[1.000,1.000]
**Low (US$ 2.88)**	0.900**	0.909**	0.902**	0.957	0.976	0.921**
(US$ 1.97 to US$ 3.96)	[0.817,0.993]	[0.837,0.986]	[0.823,0.989]	[0.903,1.015]	[0.943,1.011]	[0.856,0.992]
**Intermediate (US$ 5.36)**	0.832**	0.796***	0.738***	0.892**	0.981	0.853***
(US$ 3.97 to US$ 7.09)	[0.722,0.960]	[0.715,0.886]	[0.642,0.849]	[0.806,0.987]	[0.940,1.024]	[0.779,0.934]
**High (US$ 20.45)**	0.722***	0.640***	0.541***	0.784***	0.988	0.741***
(US$ 7.10 or more)	[0.601,0.867]	[0.552,0.742]	[0.452,0.647]	[0.679,0.906]	[0.928,1.052]	[0.651,0.844]
**Control Variables**						
Gini Index	1.05	0.951	1.039	1.143***	1.086***	0.989
	[0.963,1.145]	[0.870,1.040]	[0.947,1.139]	[1.051,1.244]	[1.036,1.139]	[0.943,1.037]
Primary Education (% population)	0.918**	0.945	0.936	0.969	1.0000	1.026
	[0.845,0.997]	[0.863,1.035]	[0.846,1.035]	[0.912,1.030]	[0.966,1.035]	[0.947,1.112]
Education expenditure (% GDP)	0.884***	0.949	0.959	0.963	0.959**	0.956**
	[0.809,0.967]	[0.870,1.036]	[0.879,1.046]	[0.900,1.031]	[0.921,0.998]	[0.915,0.998]
Piped water (% population)	0.789***	0.816***	0.95	0.978	0.994	1
	[0.757,0.822]	[0.705,0.943]	[0.856,1.053]	[0.903,1.060]	[0.968,1.021]	[0.979,1.022]
Adequate sanitation (% population)	0.891**	0.881	0.868*	0.865***	0.939***	1.006
	[0.802,0.991]	[0.732,1.060]	[0.742,1.017]	[0.814,0.919]	[0.924,0.954]	[0.966,1.047]
Nurse rate (per 1,000 population)	0.772***	0.816***	0.799***	0.830***	0.893***	0.992
	[0.705,0.845]	[0.758,0.879]	[0.750,0.852]	[0.781,0.881]	[0.859,0.929]	[0.953,1.032]
Hospital bed rate (per 1,000 population)	1.005	1.011	0.998	0.963*	0.988	1.013
	[0.951,1.062]	[0.958,1.066]	[0.945,1.055]	[0.926,1.002]	[0.970,1.006]	[0.976,1.051]
Health expenditure (% GDP)	0.958	0.996	1.02	0.942*	0.949***	1.044**
	[0.881,1.043]	[0.920,1.077]	[0.906,1.148]	[0.881,1.008]	[0.922,0.976]	[1.009,1.082]
Millitary expenditure (% GDP)	1.173**	1.139**	1.135**	1.086**	1.028	1.058
	[1.032,1.334]	[1.008,1.287]	[1.004,1.284]	[1.004,1.175]	[0.990,1.067]	[0.964,1.161]
Time trend control	Yes	Yes	Yes	Yes	Yes	Yes
**Number of observations**	2,368	2,376	2,376	2,376	2,376	2,376
**Number of countries**	132	132	132	132	132	132

Source: Author's data analysis for 2,793 observations – 133 countries/territories in the World, over 21 years (from 2001 to 2021).
Note: Data are in Rate Ratio (RR) coefficients (95% CI) unless otherwise specified. The confidence intervals are in parentheses. Time shocks are controls for specific years of economic and health crisis (2008, 2015, 2019, 2020 and 2021). The symbols '***' , '**' and '*' denote significance at 1%, 5%, and 10% respectively

WEB TABLE 19. Rate Ratios from the fixed effect Poisson models for the association between mortality rates and USAID per capita using categorical variables, by causes of death and USAID health funding; from 2001-21, in selected countries/territories.

VARIABLES	HIV/AIDS		MALARIA		MATERNAL	
	USAID (total)	USAID (Health)	USAID (total)	USAID (Health)	USAID (total)	USAID (Health)
**USAID per capita**						
**Baseline (US$ 0.00)**	1	1	1	1	1	1
	[1.000,1.000]	[1.000,1.000]	[1.000,1.000]	[1.000,1.000]	[1.000,1.000]	[1.000,1.000]
**Low (US$ 0.71)**	0.849*	0.830**	0.900**	0.935*	0.984*	0.910*
	[0.628,1.147]	[0.712,0.969]	[0.819,0.990]	[0.872,1.002]	[0.923,1.049]	[0.817,1.014]
**Intermediate (US$ 1.37)**	0.834*	0.806**	0.933**	0.776***	0.893**	0.832**
	[0.589,0.940]	[0.651,0.996]	[0.876,0.994]	[0.696,0.866]	[0.806,0.989]	[0.721,0.960]
**High (US$ 5.76)**	0.528***	0.499***	0.879***	0.584***	0.782***	0.668***
	[0.369,0.754]	[0.386,0.645]	[0.800,0.967]	[0.486,0.703]	[0.697,0.878]	[0.569,0.784]
**Control Variables**						
Gini Index	0.858	0.915	0.983	0.977	0.951	0.930
	[0.618,1.189]	[0.690,1.214]	[0.839,1.151]	[0.838,1.138]	[0.877,1.031]	[0.839,1.030]
Primary Education (% population)	0.655***	0.813	0.830**	0.837**	0.940	0.954
	[0.501,0.856]	[0.627,1.054]	[0.695,0.992]	[0.710,0.987]	[0.843,1.049]	[0.847,1.074]
Education expenditure (% GDP)	0.889	1.085	0.831***	0.900**	0.871***	0.894***
	[0.648,1.219]	[0.898,1.311]	[0.735,0.940]	[0.819,0.990]	[0.800,0.948]	[0.835,0.956]
Piped water (% population)	1.036	1.149	0.661***	0.569***	0.614***	0.591***
	[0.779,1.378]	[0.809,1.631]	[0.557,0.784]	[0.487,0.664]	[0.571,0.661]	[0.547,0.637]
Adequate sanitation (% population)	0.778**	0.734***	0.802***	0.824***	0.738***	0.736***
	[0.614,0.987]	[0.586,0.918]	[0.706,0.911]	[0.722,0.940]	[0.648,0.841]	[0.645,0.840]
Nurse rate (per 1,000 population)	1.186*	1.127**	1.178	1.182	0.972	0.978
	[0.973,1.447]	[1.005,1.263]	[0.935,1.484]	[0.955,1.463]	[0.936,1.009]	[0.942,1.015]
Hospital bed rate (per 1,000 population)	1.098	1.121	0.915	0.933	0.998	1.008
	[0.851,1.417]	[0.899,1.397]	[0.805,1.040]	[0.835,1.042]	[0.938,1.063]	[0.937,1.085]
Health expenditure (% GDP)	1.362**	1.244*	1.233*	1.182*	1.243*	1.189*
	[1.067,1.738]	[0.999,1.550]	[0.985,1.543]	[0.979,1.427]	[0.975,1.586]	[0.979,1.445]
Millitary expenditure (% GDP)	1.179**	1.214***	1.102**	1.075**	0.997	1.010
	[1.017,1.368]	[1.052,1.400]	[1.005,1.207]	[1.008,1.147]	[0.903,1.100]	[0.928,1.100]
Time trend control	Yes	Yes	Yes	Yes	Yes	Yes
**Number of observations**	2793	2793	2079	2079	2793	2793
**Number of countries**	133	133	99	99	133	133

Source: Author's data analysis for 2,793 observations – 133 countries/territories in the World, over 21 years (from 2001 to 2021).
Note: Data are in Rate Ratio (RR) coefficients (95% CI) unless otherwise specified. The confidence intervals are in parentheses. Time shocks are controls for specific years of economic and health crisis (2008, 2015, 2019, 2020 and 2021). The symbols '***' , '**' and '*' denote significance at 1%, 5%, and 10% respectively

WEB TABLE 20. Rate Ratios from the fixed effect Poisson models for the association between mortality rates and USAID per capita using categorical variables, based on USAID's share of total global donor funding; from 2001-21, in selected countries/territories.

VARIABLES	USAID's share among official donors		
	All Aid	Health Aid	Humanitary Aid
**USAID per capita**			
**Baseline (US$ 0.45)**	1	1	1
(US$ 0.00 to US$ 1.96)	[1.000,1.000]	[1.000,1.000]	[1.000,1.000]
**Low (US$ 2.88)**	0.947**	0.951**	0.947**
(US$ 1.97 to US$ 3.96)	[0.904,0.992]	[0.909,0.994]	[0.901,0.994]
**Intermediate (US$ 5.36)**	0.913***	0.916***	0.911***
(US$ 3.97 to US$ 7.09)	[0.857,0.973]	[0.864,0.971]	[0.853,0.973]
**High (US$ 20.45)**	0.859***	0.866***	0.856***
(US$ 7.10 or more)	[0.791,0.933]	[0.800,0.936]	[0.786,0.931]
**USAID's share among official donors**			
All type (%)	0.958***		
	[0.934,0.982]		
Health Aid (%)		0.941***	
		[0.913,0.970]	
Humanitary Aid (%)			0.963***
			[0.946,0.980]
**Control Variables**			
Gini Index	1.052**	1.053**	1.053**
	[1.008,1.098]	[1.011,1.097]	[1.009,1.098]
Primary Education (% population)	0.983	0.986	0.983
	[0.942,1.026]	[0.945,1.029]	[0.943,1.026]
Education expenditure (% GDP)	0.939**	0.943*	0.936**
	[0.882,1.000]	[0.885,1.004]	[0.878,0.999]
Piped water (% population)	0.977*	0.994	0.974**
	[0.951,1.003]	[0.967,1.022]	[0.951,0.996]
Adequate sanitation (% population)	0.911***	0.916***	0.910***
	[0.858,0.967]	[0.865,0.969]	[0.854,0.970]
Nurse rate (per 1,000 population)	0.893***	0.884***	0.890***
	[0.858,0.929]	[0.854,0.915]	[0.857,0.923]
Hospital bed rate (per 1,000 population)	0.994	0.995	0.995
	[0.970,1.017]	[0.974,1.016]	[0.970,1.020]
Health expenditure (% GDP)	0.943*	0.946*	0.943*
	[0.886,1.004]	[0.888,1.007]	[0.885,1.004]
Millitary expenditure (% GDP)	1.076**	1.071**	1.076**
	[1.011,1.145]	[1.009,1.136]	[1.010,1.147]
Time trend control	Yes	Yes	Yes
**Number of observations**	2793	2793	2793
**Number of countries**	133	133	133

Source: Author's data analysis for 2,793 observations – 133 countries/territories in the World, over 21 years (from 2001 to 2021). **Note:** Data are in Rate Ratio (RR) coefficients (95% CI) unless otherwise specified. The confidence intervals are in parentheses. Time shocks are controls for specific years of economic and health crisis (2008, 2015, 2019, 2020 and 2021). The symbols '***' , '**' and '*' denote significance at 1%, 5%, and 10% respectively.

5.2. Triangulation – Difference-in-difference with Propensity Score Matching

We examined the effect of USAID per capita disbursements on mortality rates using a Difference-in-Differences (DiD) approach, complemented by Propensity Score Matching (PSM) as a triangulation method.[29] To ensure comparability in baseline mortality levels, we restricted the sample to low-income (LIC) and lower-middle-income countries (LMIC). A total of 192 countries/territories were included in the analysis, divided into two groups: those with no or low USAID disbursements (n = 44, control group), and those with intermediate to high USAID disbursements (n = 148, treatment group). Mortality rates were assessed for the years 2001 and 2019.

The year 2001 was selected as the baseline because it marks the beginning of available USAID disbursement data, while 2019 was chosen to avoid the confounding effects of mortality changes associated with the COVID-19 pandemic. The categorization of USAID per capita levels follows the same structure applied in the main regression analyses: countries in the first quartile (none or low disbursements) were assigned to the control group (value = 0), whereas those in the second to fourth quartiles (intermediate to high and consolidated disbursements) were assigned to the treatment group (value = 1).

Web Figures 6 and 7 provide empirical support for the key assumptions underlying the PSM and DiD methodologies—common support and parallel trends, respectively—thereby strengthening the validity of the estimated effects.

WEB FIGURE 6. Common support between treatment and control group mortality rates for USAID per capita disbursement.

Age-standardized mortality rate (ASMR) **Under-five mortality rate (U5MR)**

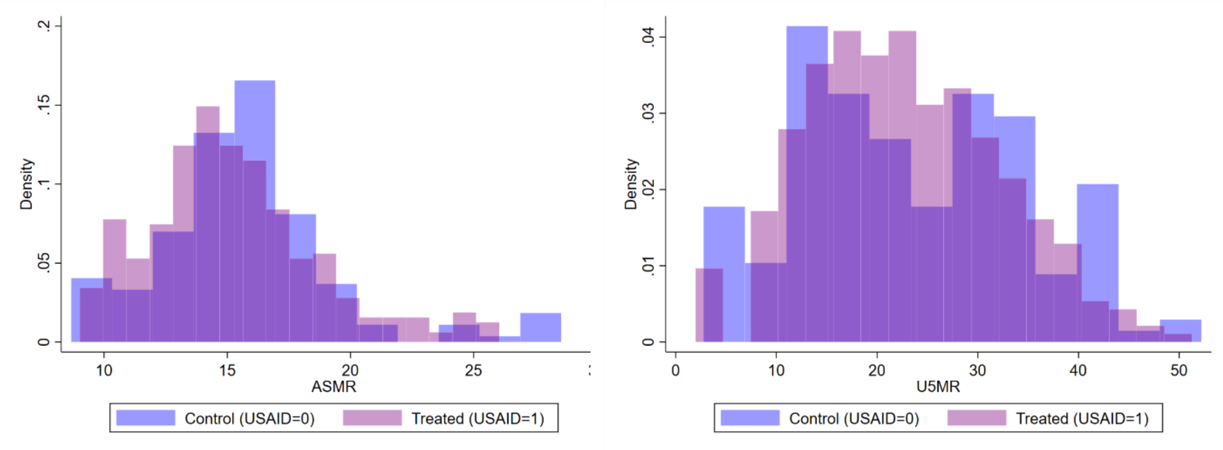

WEB FIGURE 7. Parallel trends assumption between treatment and control group mortality rates for USAID per capita disbursement.

Age-standardized mortality rate (ASMR) **Under-five mortality rate (U5MR)**

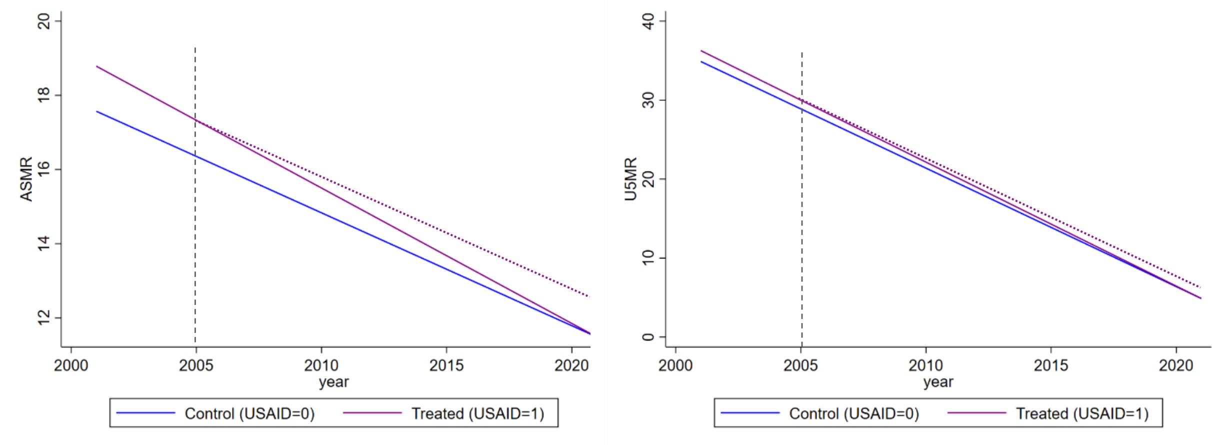

Note: In the absence of pre-treatment data on USAID disbursements, we designated 2005 as the starting point of the historical series and compared mortality trends before and after this reference year. It is worth noting that the parallel trends assumption holds regardless of the specific year chosen within the early part of the series.

We employed two complementary approaches to estimate the Difference-in-Differences (DiD) with Propensity Score Matching (PSM). First, we used a conventional method via the STATA *diff* command. Second, we applied the step-by-step procedure outlined in the World Bank's handbook,[33] which estimates DiD using fixed-effects Poisson panel models and reports coefficients as rate ratios (RR).

Web Table 21 presents the results from the DiD analysis combined with PSM. Even after adjusting for observable characteristics of countries/territories through kernel matching, the first difference (2001) shows that areas receiving higher per capita USAID disbursements had higher overall (ASMR) and child (Under-5) mortality rates compared to those with low or no disbursements. By 2019 (the second difference), both groups experienced reductions in mortality; however, the decline was significantly greater in countries/territories with higher USAID support, yielding a statistically significant DiD estimate.

Web Table 22 presents findings from the fixed-effects Poisson panel models. These models confirm that higher per capita USAID disbursements were associated with greater reductions in overall and infant mortality compared to areas with little or no support, with a rate ratio (RR) of 0.992 (95% CI: 0.988–0.996).

Together, these results suggest that USAID contributions played a meaningful role in reducing both overall and under-5 mortality rates. The DiD consistency between PSM-based and Poisson panel model estimates reinforces the findings reported in the main manuscript, enhancing the robustness of our conclusions through methodological triangulation.

WEB TABLE 21. Difference-in-Differences Estimates with Propensity Score Matching of the Association between Intermediate to High USAID Per Capita Disbursements and Mortality Rates (Overall and Child) in 2001 and 2019 among Low- and Lower-Middle-Income Countries/Territories

	**Overall** ASMR	**Child** Under 5 years
Before (2001)		
Control	8.196	43.378
Treated	11.489	45.234
1st Difference (T-C)	3.293***	1.857***
	(0.008)	(0.001)
After (2019)		
Control	6.619	29.500
Treated	5.222	26.956
2nd Difference (T-C)	-1.397***	-2.544***
	(0.007)	(0.001)
**Diff-in-Diff**	**-4.690***<br>(0.008)**	**-4.400***<br>(0.001)**

WEB TABLE 22. Rate Ratios from the difference-in-difference fixed effect Poisson models for the association between mortality rates (overall and child) with intermediate to high USAID disbursement, in 2004 and 2019, in Low Income and Lower-Middle Income countries/territories.

**VARIABLES**	**ASMR**	**U5MR**
**USAID per capita (dumies)**		
USAID_dd	10760659.3***	4.26964e+37***
	[1289.718,8.978e+10]	[6.398e+30,2.849e+44]
USAID Intermediate to high	0.992***	0.958***
	[0.988,0.996]	[0.950,0.965]
**Control Variables**		
Gini Index	0.956	0.948
	[0.901,1.015]	[0.761,1.181]
Primary Education (% population)	0.932	0.984
	[0.847,1.026]	[0.636,1.523]
Education expenditure (% GDP)	1.127***	0.945
	[1.030,1.233]	[0.774,1.152]
Piped water (% population)	1.07	0.831
	[0.962,1.190]	[0.500,1.381]
Adequate sanitation (% population)	0.913*	0.645**

VARIABLES	ASMR	U5MR
	[0.832,1.002]	[0.448,0.928]
Nurse rate (per 1,000 population)	1.023	0.995
	[0.931,1.124]	[0.645,1.535]
Hospital bed rate (per 1,000 population)	0.998	1.118
	[0.936,1.064]	[0.812,1.538]
Health expenditure (% GDP)	1.021	0.874
	[0.945,1.103]	[0.724,1.056]
Millitary expenditure (% GDP)	0.994	1.232***
	[0.899,1.101]	[1.070,1.419]
**Number of observations**	160	160
**Number of countries**	80	80

Source: Author's data analysis for 160 observations – 80 countries/territories in the World, over 2 years (from 2001 and 2021).
Note: Data are in Rate Ratio (RR) coefficients (95% CI) unless otherwise specified. The confidence intervals are in parentheses. The symbols '***' , '**' and '*' denote significance at 1%, 5%, and 10% respectively

5.3. Deaths prevented by USAID during 2001-2021

To simulate deaths prevented due to USAID programs in 2001-21 period, we predicted coefficient $E(Y_{it} \mid X)$, here X represents the set of covariates including the interventions, and Y_{it} are the mortality rate at country *i*, in year *t*. Thus, the Monte Carlo methodology was used to get more accurate results. It can be summarized in the following steps:

1. Predict the intervention values for the retrospective period (2001-2021) initially using the same coefficients as the main model (Table 2 in the manuscript);
2. Simulate a new Y_{it} from the Poisson distribution using the estimated parameters from the retrospective study, changing only the USAID coefficient to rescue the baseline (0 funding) and comparing with real deaths, making the difference between them and adding them up over the different years;
3. Get the predictions $E(Y_{it} \mid X)$ using the new simulated variable Y_{it}, here X represents the set of covariates including the interventions.
4. Get back to step 1.

For each outcome, 100,000 simulations were performed. Web-Table 18 shows this simulation, in which USAID disbursement per capita **avoided 91.8 million deaths** (i.e. deaths of all ages and from all causes, 95% IC: 85.7 million – 98.3 million) and **30.4 under-five deaths** (95% IC: 26 million – 35.5 million) between 2001-21 in the hypothetical case where USAID did not exist.

WEB TABLE 23. Deaths prevented by USAID disbursement per capita during 2001-21.

Averted deaths	Estimate	95% CI		% of total deaths
		LI	LS	
**Overall (ASMR)**	91,839,663	85,690,135	98,291,626	7.0%
**By age group**				
Infancy (0 to 1 year)	13,286,197	11,536,769	15,287,795	11.5%
Preschool (2 to 4 years)	8,665,606	7,203,360	10,415,507	27.4%
Child (Under 5 years)	30,391,980	26,023,132	35,482,636	17.6%
Preschool (5 to 9 years)	1,047,777	936,488	1,173,273	9.0%
**By causes of deaths**				
Tuberculosis	4,728,983	4,182,056	5,275,910	14.1%
HIV/AIDS	25,547,337	21,937,725	29,154,963	51.8%
Maternal mortality	657,363	611,930	702,797	11.4%
Lower respiratory infections	8,903,053	7,753,598	10,052,508	16.4%
Nutritional Deficiencies	2,093,120	1,768,369	2,417,871	26.0%
Diarrhoeal diseases	11,225,295	8,971,035	13,479,555	24.3%
Malaria	8,019,681	7,303,074	8,736,289	32.3%
Neglected tropical diseases	8,873,267	8,084,532	9,662,002	31.5%

5.3.1. Validation by Prevented Fraction for the Population method

To validate the number of deaths averted between 2001 and 2021 attributable to USAID per capita disbursements, we employed the Prevented Fraction for the Population (PFP) method.[34]

The PFP is an epidemiological measure used to estimate the proportion of adverse events (such as deaths, infections, or hospitalizations) that were prevented in a population due to a specific intervention or exposure. It reflects the effectiveness of an intervention at the population level by quantifying the reduction in the incidence of an outcome compared to what would have been expected without the intervention. Mathematically, PFP is calculated according to the formula below.

$$PFP = \frac{P_d(1 - RR)}{[1 - (1 - RR)(1 - P_d)]}$$

where Pd is the prevalence of exposure among disease cases, and RR is the relative risk.

This method is particularly useful in public health evaluations, where it is important to attribute changes in population health outcomes to large-scale programs or policies. By applying PFP, it can estimate the overall impact of interventions such as vaccination programs, disease control initiatives, or, in this case, development assistance efforts supported by USAID. The table below shows the results of applying the PFP method to the survey data.

WEB TABLE 24. Deaths prevented by USAID disbursement per capita during 2001-21 – PFP method.

Averted deaths	Estimate	95% CI		% of total deaths
		LI	LS	
**Overall (ASMR)**	93,553,484	86,892,607	100,922,629	7.7%
**Under-5**	28,686,651	25,416,373	32,559,349	20.3%

The PFP calculation estimated that 93.5 million deaths were averted across all age groups and 28.7 million among children under five years of age. These results are consistent with the estimates of 91.8 million (overall) and 30.4 million (under-five) obtained through the proposed method, and fall within its confidence intervals, thereby demonstrating the robustness and consistency of the findings.

PART III – FORECASTING ANALYSIS

6. DESCRIPTION OF THE FORECASTING METHODOLOGY

The following section provides details of the forecasting process in accordance with standard international modelling reporting guidelines (ISPOR-SMDM). The modelling approach adopted for this study was developed based on two stages.

This section outlines the forecasting methodology in accordance with international modeling reporting standards (ISPOR-SMDM guidelines).[35–37]

The forecasting strategy employed in this study follows a two-step approach.

In the first step, we constructed a synthetic cohort for the period 2022–2030, encompassing 133 countries from the low-income, lower-middle-income, and upper-middle-income economic blocks. This cohort extends the longitudinal dataset used in the retrospective analysis, which covered the years 2001–2021. The projections within the synthetic cohort aim to replicate plausible future scenarios under potential changes in USAID policies. The exposure variable will be the annual per capita monetary value of USAID benefits to the countries along the study period (our observation unit) categorized in four levels (more details in Section 3).

Furthermore, two main policy scenarios were evaluated.

- **Baseline scenario**: Assumes that USAID investment and funding levels remain constant from 2023 (last real value with fiscal year closed and reported by entities) through 2030.
- **1st Alternative scenario**: Simulates a significant funding reduction, with an 83% cut in USAID support in 2025. From 2026 to 2030, all countries in the cohort are assumed to transition to the lowest funding tier, simulating a progressive dismantling of the project.

The second step involves the forecasting procedure itself. This was carried out using a Monte Carlo simulation framework, applying the same fixed-effects regression models described in Section 4. The goal was to predict the general age-standardized number of deaths (ASMR) per 1,000 inhabitants—calculated according to WHO methodology— and the under five child mortality (U5MR) per 1,000 live births, for each year within the synthetic cohort.
were predicted as the responses of the same multivariate fixed effects regressions described in section

7. PURPOSE OF THE FORECASTING AND ITS APPLICATIONS

The overall goal is to simulate the dismantling policies that are being implemented in USAID funding around the world. Specifically, the overall impact on the number of deaths that the cuts and defunding, widely reported in the global media, can cause.

8. DATA SOURCE AND INPUTS

The panel data used in this study combined aggregated demographic, socioeconomic, health and USAID info from several sources (all sources are publicly available and listed in the Web-Table 4, in the paper appendix.)

In the absence of reference values from the literature, we categorised all USAID information using the quartiles from the lower-income countries. The levels adopted in this study are, baseline (0 to \$1.96 per capita), low (25th percentile, \$1.97 to \$3.96), intermediate (50th percentile or median, \$3.97 to \$7.09), and high (75th percentile, \$7.10 and above). This classification based on the lower-income countries better captures the context in which USAID primarily operates, enabling a more accurate interpretation of funding patterns.

As input in to the modelling and forecasting phases, all relevant time-variant demographic, socioeconomic, and healthcare- adjusting variables - were included in the models: gross domestic

product (GDP) per capita at purchasing power parity (GDP pc PPP); public expenditures on education, health, and the military (each on as a percentage of GDP); literacy rate; Gini index; the percentage of households with inadequate sanitation and with access to piped water; the number of doctors per 1,000 population; and the number of hospital beds per 1,000 population.

9. POISSON MULTIVARIABLE REGRESSION MODELS WITH ROBUST STANDARD ERRORS AND FIXED-EFFECTS

To better capture the effects of interest in our study, we employed a **Generalized Linear Model (GLM) with a Poisson distribution,** incorporating **robust standard errors** to account for potential misspecification of the variance structure due to the panel data design.[16,17,19,21,22]

Then, let Y_{it} be the observed count of deaths (general or under-five) and assuming $Y_{it} \sim Poisson(\mu_{it})$, the linear predictor of the model, considering the population offset and country fixed effect, is

$$\log(\mu_{it}) = \log(pop_{it}) + \beta_0 + \boldsymbol{X_{it}\beta} + \alpha_i, \quad (1)$$

where μ_{it} is the expected number of the event of interest, pop_{it} and $\boldsymbol{X_{it}}$ are the population of interest size and the vector of covariates in the time t for the observation i, respectively, β_0 the intercept, $\boldsymbol{\beta}$ is the corresponding vector of regression coefficients and α_i denotes the unit-specific fixed effect (assumed time-invariant).

10. CALIBRATION, TIME TREND, AND MONTE CARLO SIMULATION

10.1. Calibration

In the forecasting analysis, all parameter estimates and their respective standard errors were derived from the retrospective dataset and the model detailed in Section 4. However, to minimize errors in the projection outputs, we decided to calibrate the intercept β_0 of the regression model. The calibration process was based on data from 2017 to 2019, and a Monte Carlo (see Section 5.3 for complete detail) procedure was applied to simulate different scenarios by varying the value of β_0 until a satisfactory Mean Squared Error (MSE) was achieved for either the ASMR or U5MR.

We initiated the calibration using the estimated value of β_0 obtained from the retrospective model, and subsequently explored numerical sequences with incremental adjustments—typically varying by 0.5, 0.3, 0.1, or 0.01 in steps of five candidate values—to progressively refine the selection and minimize the MSRE.

The Web Table 25 presents the final results obtained after the refinement process, including the calibrated values that yielded the lowest MSRE.

WEB TABLE 25. Final Estimates After Model Refinement and Calibration Based on Minimum MSRE

ASMR		U5MR	
Estimate of β_0	MSRE	Estimate of β_0	MSRE
-4.12	0.006	-3.98	0.094
-4.11	0.023	-3.97	0.024
-4.10	0.061	-3.96	0.014
-4.09	0.114	-3.95	0.001
-4.08	0.184	-3.94	0.012

In this way, the calibrated values of β_0 for the ASMR and U5MR models were determined to be -4.12 and -3.95, respectively.

10.2. Time trend

To estimate the underlying time trend in mortality rates, we fitted a linear regression model using the logarithm of the mortality rate per 1,000 inhabitants as the dependent variable and the calendar year as the independent Variable.

First, we excluded the years 2020 and 2021 due to the direct impact of the COVID-19 pandemic on death counts, which could distort the long-term trend. After this, we applied a logarithmic transformation to the mortality rate in order to stabilize the variance, better capture proportional (multiplicative) changes, and improve the model's fit within a standard Gaussian linear regression framework (see Web Figure 8).

In this framework, the estimated coefficient associated with the year variable can be interpreted as the average annual rate of change in the log mortality rate.

WEB FIGURE 8. Time trend of log mortality rate per 1,000 inhabitants (Excluding 2020-2021).

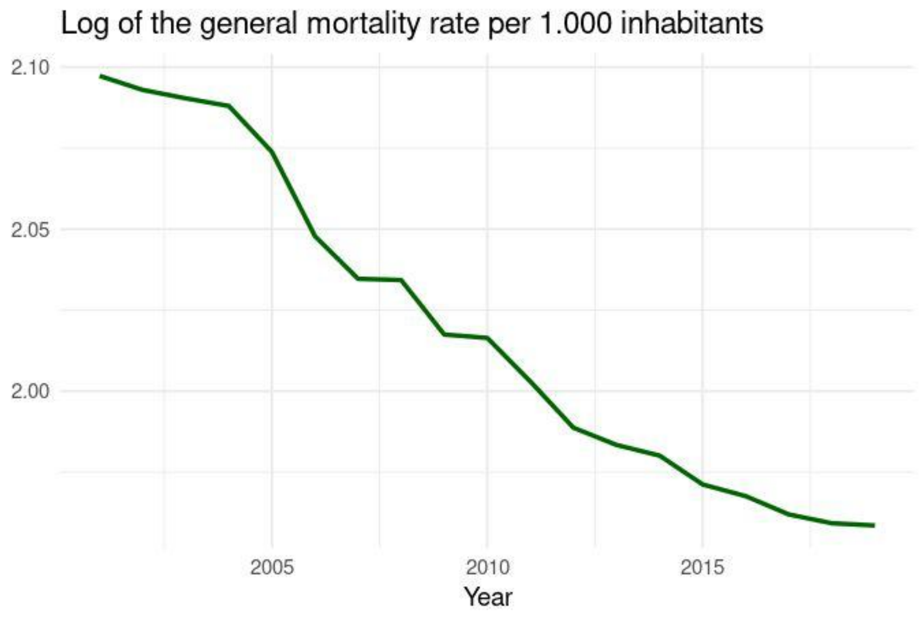

The estimated coefficient associated with the year variable is -0.009, with a standard error of 0.0004, indicating a statistically significant decreasing trend in the log mortality rate over time. A residual analysis was conducted to assess the adequacy of the linear regression assumptions. The results suggest that the normality assumption is reasonably satisfied, as indicated by the Shapiro-Wilk test (p-value = 0.2812). In addition, the residuals appear to be symmetrically distributed around zero, supporting the appropriateness of the model.

WEB FIGURE 9. Q-Q plot of the residuals from the linear regression model fitted to the log-transformed mortality rate per 1,000 inhabitants.

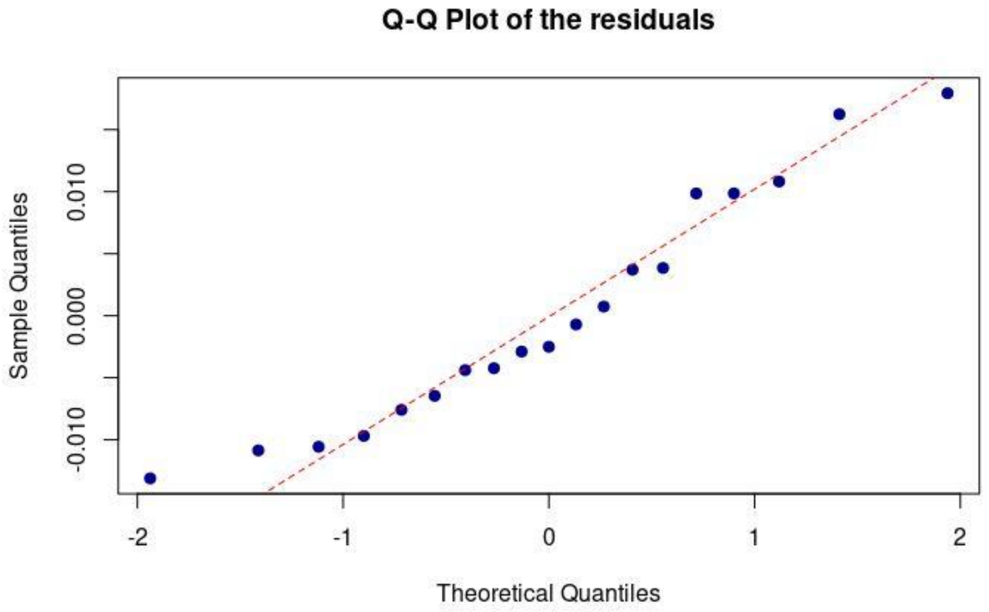

The same procedure was applied to the U5MR, resulting in an estimated coefficient of -0.034 with a standard error of 0.0004. As in the previous model, the regression assumptions were assessed and validated. The Shapiro-Wilk test for normality yielded a p-value of 0.89, and the residuals were reasonably distributed (see Web Figure 11).

WEB FIGURE 10. Time trend of log under-five mortality rate per 1,000 live births (Excluding 2020-2021).

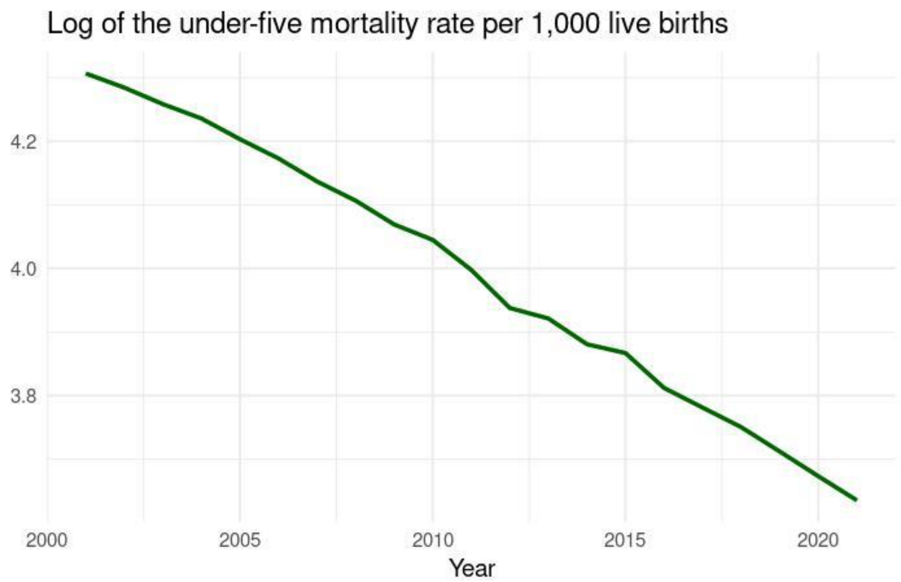

WEB FIGURE 11. Q-Q plot of the residuals from the linear regression model fitted to the log-transformed U5MR per 1,000 live births.

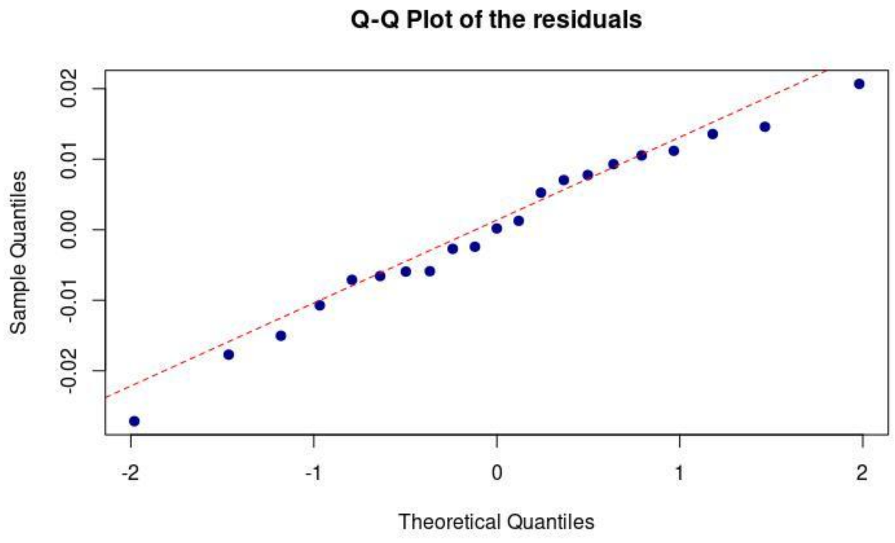

10.3. Monte Carlo Simulation

The study employed 1,000 Monte Carlo (MC) replications. This number was selected after confirming the stability of simulation outputs, which consistently converged between 300 and 500 replications, in accordance with the recommendations of Briggs et al. (2012). All computational procedures were carried out using the free statistical software **R (version 4.2.1)** in a **Linux environment**.

For each MC replication and policy scenario, the following prediction procedure was applied:

1. Based on the estimates obtained from the retrospective analysis—using the multivariable fixed-effects Poisson model described in Section 4—along with the time trend and their respective confidence intervals, we randomly selected a value for each parameter based on the normal distribution with mean and standard error being the point estimate and stardard error from the regression analysis.
2. Using the estimates from Step 1 and the structure of the synthetic cohort, we calculated the expected mean number of deaths for each observation, defined at the country-year level.
3. A count outcome was then generated by drawing from a Poisson distribution, with the previously estimated mean used as the distribution's parameter.
4. Steps 1 to 3 were repeated until the desired number of MC replications was reached.

It is important to highlight that Step 1 of the algorithm plays a key role in introducing randomness and uncertainty into the study, which is a crucial aspect in micro-simulation approaches.

The final predictions and corresponding uncertainty intervals for each outcome variable were derived from the simulation results: the mean of the 1,000 replications was used as the point estimate, while the 2.5th and 97.5th percentiles were used to construct 95% confidence intervals.

11. VALIDATION

To validate our approach, we compared the under-five mortality rates (U5MR) simulated by our model with the official U5MR estimates available from UNICEF (source https://data.unicef.org/topic/child-survival/under-five-mortality/#data) for the 133 countries included in the study. The comparison focused on the years 2022 and 2023, during which no changes were observed in variables such as USAID per capita or other intervention-related indicators.

As shown in Web Figure 12, the observed values from UNICEF fall within the uncertainty intervals of our model, indicating strong agreement between the simulated and empirical data. Table 1 also presents the real values, point estimates and the uncertainty intervals.

WEB FIGURE 12. Simulated Under-Five Mortality Rates with Uncertainty Intervals and Observed Values from UNICEF (2022–2023).

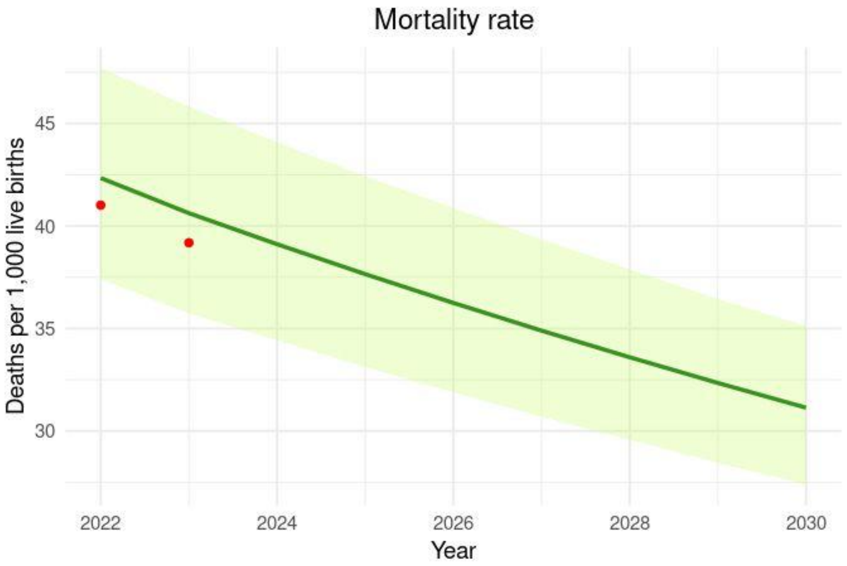

WEB TABLE 26. Comparison between Simulated and Observed Under-Five Mortality Rates (U5MR) in 2022 and 2023 across the 133 Countries.

Year	Real Value	Predicted Value	Uncertainty Interval
2022	41.02	42.31	[37.4 - 47.7]
2023	39.18	40.60	[35.7 - 45.8]

12. MAIN LIMITATIONS

Although microsimulation was conducted in alignment with best practices, some limitations must be acknowledged. For instance, the impact on certain key country-level covariates could change drastically in response to the dismantling of USAID funding. Additionally, the lack of transparency regarding the precise structure and geographic allocation of the funding cuts limits our ability to accurately predict where and to what extent the effects will materialize.

These key factors may lead to an underestimation of the total number of deaths in our study, as the broader structural impacts on countries could extend beyond the direct monetary effects. In other words, the disruption caused by the potential dismantling of USAID support might affect health systems and outcomes in ways that are not fully captured by financial variables alone.

PART IV - ASSESSING THE PLAUSIBILITY OF EFFECT MAGNITUDE

13. EPIDEMIOLOGICAL PLAUSIBILITY

It is important to note that high levels of USAID funding—those associated with a 15% reduction in overall mortality—were only reached by some countries and only in specific years, as shown in Web Table 27. Although the threshold used to define "high USAID funding" is set at above US$7.10 per capita, it is important to note that this corresponds to large donations at the country level, with a minimum total annual donation in our dataset of approximately US$15,756,130.00 (e.g. Kyrgyz Republic). Furthermore, the average per capita funding within this high-funding group is US$20.50, reaching a maximum of US$315.53 per capita In absolute terms, this corresponds to an average annual donation of US$257,063,876.21 per country (e.g., Somalia), reaching as high as US$3,204,988,672.00 in some cases (e.g., Afghanistan).

Additionally, while these numbers represent the average USAID funding in the entire nation, these funds are for interventions focused on the most vulnerable populations, which often represent a minority of the population (so these funds are concentrated in smaller vulnerable populations).[38]

WEB TABLE 27. Distribution of Countries by USAID Funding Quartile, 2001–2021.

Quartile	2001	2005	2010	2015	2020	2021
1	81	75	68	65	61	54
2	23	15	16	11	20	20
3	15	18	22	26	15	16
4	14	25	27	31	37	43
**Total**	**133**	**133**	**133**	**133**	**133**	**133**

The baseline levels of extreme poverty, socioeconomic vulnerability, and mortality rates among countries receiving these high levels of funding further contextualize these figures. As shown in Web Figure 3, these countries are among the poorest and most vulnerable in the world.

While it is challenging to precisely estimate the impact of each USAID-funded intervention, they all share a common goal: targeting extremely vulnerable individuals, both socioeconomically and in terms of health. Many of these individuals live on less than US$2.15 per day, so such aid can represent a significant relative increase in income and household assets.

Empirical studies show that even modest investments can yield substantial health gains. The Preston Curve,[39] for example, illustrates that in low-income countries, life expectancy rises sharply with small increases in GDP per capita. Similar patterns are confirmed by DHS data analyses,[40,41] where slight improvements in income or wealth result in notable health benefits for children and adults.

In Brazil, our group's big data studies using quasi-experimental methods showed that modest cash transfers to the most vulnerable populations led to:

- a 60% reduction in tuberculosis incidence and mortality,[42]
- a 50% reduction in HIV/AIDS incidence and mortality,[42] and
- a 70% reduction in child mortality.[42]

While these findings highlight socioeconomic interventions, health-focused strategies—such as USAID's support for vaccination programs—can be even more cost-effective. For example, measles, yellow fever, and maternal tetanus campaigns cost less than US$2 per person,[43] and vaccination programs in LMICs are estimated to have prevented 37 million deaths between 2000 and 2019, mostly among children under five.

USAID also plays a major role in humanitarian crises, providing over 40% of global humanitarian aid in 2023. In emergencies—due to conflict, disasters, or displacement—mortality often spikes to 2–10 times the baseline, as vulnerable populations face worsening nutrition, immunity, and mental health. In these contexts, USAID-supported interventions can have immediate and life-saving effects.[44,45]

14. ROBUSTNESS OF METHODOLOGY

To assess the robustness and credibility of our findings, we employed the most appropriate and recommended statistical methodologies for our dataset and conducted a comprehensive set of sensitivity and triangulation analyses, detailed in Section 5. These included variations in model specifications (e.g., using continuous vs. categorical exposure variables, alternative thresholds, and different time trend structures), analyses incorporating the full global dataset (204 countries), and model comparisons (e.g., Poisson vs. Negative Binomial). To enhance causal inference, we performed Difference-in-Differences analyses combined with Propensity Score Matching. Importantly, we used injury-related mortality as a negative control outcome, which confirmed the specificity of the observed associations. Collectively, these analyses reinforce the stability, specificity, and external validity of our findings.

15. COMPARISON WITH EXISTING USAID IMPACT EVALUATIONS

As detailed in the main manuscript in the Discussion section, pior studies evaluating the IMPACT of USAID interventions in the past decades, conducted with different datasets, sources, and methodologies, have produced results broadly consistent with ours.

Despite methodological differences—including variations in study periods, exposure definitions, and outcome metrics—the central estimates of deaths averted, particularly those linked to direct health interventions, are broadly consistent with our findings. For instance, one prior assessment[27] estimated that USAID-funded health programs prevent approximately 3.3 million all-cause, all-age deaths annually. When extrapolated over 21 years, this yields a total of 69.3 million deaths averted (95% UI: 48.3 - 117.6)—reasonably close to our estimate of 91.8 million deaths averted (95% UI: 85.7–98.3 million) between 2001 and 2021.

Our higher estimate likely reflects the broader scope of our analysis, which captures not only the effects of health-sector interventions but also the impact of USAID investments on social determinants of health. This more comprehensive approach complements earlier evaluations focused exclusively on disease-specific or clinical interventions and helps explain the alignment of our central estimate with the upper range of previous projections.

REFERENCES

1 U.S. Foreign Assistance Dashboard. U.S. Agency for International Development (USAID). 2025; published online April 11. https://foreignassistance.gov/ (accessed April 29, 2025).

2 Charles Kenny, Justin Sandefur. New Estimates of the USAID Cuts. CGD. 2025; published online March 20. https://www.cgdev.org/blog/new-estimates-usaid-cuts (accessed April 23, 2025).

3 Jennifer Kates, Anna Rouw, Stephanie Oum, Adam Wexler. How Much Global Health Funding Goes Through USAID? KFF. 2025; published online Feb 7. https://www.kff.org/policy-watch/how-much-global-health-funding-goes-through-usaid/ (accessed April 23, 2025).

4 Jennifer Kates, Kellie Moss. The Outlook for PEPFAR in 2025 and Beyond. KFF. 2025; published online Feb 20. https://www.kff.org/policy-watch/the-outlook-for-pepfar-in-2025-and-beyond/ (accessed April 23, 2025).

5 USAID. The President's Malaria Initiative - 18th Annual Report to Congress. 2024 https://reliefweb.int/report/democratic-republic-congo/presidents-malaria-initiative-18th-annual-report-congress-april-2024 (accessed April 23, 2025).

6 Symons TL, Lubinda J, McPhail M, *et al.* Estimating the potential malaria morbidity and mortality avertable by the President's Malaria Initiative in 2025: a geospatial modelling analysis. 2025; published online March 4. DOI:10.1101/2025.02.28.25323072.

7 Reevaluating And Realigning United States Foreign Aid. White House. The White House. 2025; published online Jan 20. https://www.whitehouse.gov/presidential-actions/2025/01/reevaluating-and-realigning-united-states-foreign-aid/ (accessed April 23, 2025).

8 Jennifer Hansler, Kit Maher. Rubio says Trump administration canceling 83% of programs at USAID and intends to move remaining ones to State Department. CNN. 2025; published online March 10. https://edition.cnn.com/2025/03/10/politics/rubio-usaid-contracts-state-department/index.html (accessed April 23, 2025).

9 TB Specific Impact Survey Initial Summary Report. The TB Community Coordination Hub. 2025. https://drive.google.com/file/d/1gqrTdToSyoJjCxWNfRGP9BlY_dmenDq8/view (accessed April 23, 2025).

10 Ryan Huang. UN World Food Program closes southern Africa office amid US aid cuts. JURISTnews. 2025; published online March 4. https://www.jurist.org/news/2025/03/un-world-food-program-closes-southern-africa-office-amid-us-aid-cuts/ (accessed April 23, 2025).

11 Operation Inherent Resolve Lead Inspector General Quarterly Report to Congress, January 1, 2025 – March 31, 2025. 2025 https://oig.usaid.gov/node/7640 (accessed April 29, 2025).

12 Justine Calma. Warning systems for floods, hurricanes, and famine are suffering from Donald Trump's data purge. The Verge. 2025; published online April 30. https://www.theverge.com/tech/656714/trump-disaster-hurricane-flood-famine-data-purge-usaid?utm_source=chatgpt.com (accessed April 30, 2025).

13 Rasella D, Aquino R, Barreto ML. Impact of the Family Health Program on the quality of vital information and reduction of child unattended deaths in Brazil: an ecological longitudinal study. 2010 http://www.biomedcentral.com/1471-2458/10/380.

14 Hone T, Rasella D, Barreto ML, Majeed A, Millett C. Association between expansion of primary healthcare and racial inequalities in mortality amenable to primary care in Brazil: A national longitudinal analysis. *PLoS Med* 2017; **14**: e1002306.

15 Rasella D, Harhay MO, Pamponet ML, Aquino R, Barreto ML. Impact of primary health care on mortality from heart and cerebrovascular diseases in Brazil: a nationwide analysis of longitudinal data. *BMJ* 2014; **349**: g4014–g4014.

16 Rasella D, Aquino R, Santos CAT, Paes-Sousa R, Barreto ML. Effect of a conditional cash transfer programme on childhood mortality: A nationwide analysis of Brazilian municipalities. *The Lancet* 2013; **382**: 57–64.

17 Aransiola TJ, Ordoñez JA, Cavalcanti DM, Alves de Sampaio Morais G, de Oliveira Ramos D, Rasella D. The combined effect of social pensions and cash transfers on child mortality: evaluating the last two decades in Brazil and projecting their mitigating effect during the global economic crisis. *The Lancet Regional Health - Americas* 2023; **27**: 100618.

18 Rasella D, Alves FJO, Rebouças P, *et al.* Long-term impact of a conditional cash transfer programme on maternal mortality: a nationwide analysis of Brazilian longitudinal data. *BMC Med* 2021; **19**. DOI:10.1186/s12916-021-01994-7.

19 Moncayo AL, Cavalcanti DM, Ordoñez JA, *et al.* Can primary health care mitigate the effects of economic crises on child health in Latin America? An integrated multicountry evaluation and forecasting analysis. *Lancet Glob Health* 2024; **12**: e938–46.

20 de Sampaio Morais GA, Magno L, Silva AF, *et al.* Effect of a conditional cash transfer programme on AIDS incidence, hospitalisations, and mortality in Brazil: a longitudinal ecological study. *Lancet HIV* 2022; **9**: e690–9.

21 Cavalcanti DM, Ordoñez JA, Aransiola T, *et al.* Evaluation and Forecasting Analysis of the Association of Conditional Cash Transfer With Child Mortality in Latin America, 2000-2030. *JAMA Netw Open* 2023; **6**: e2323489.

22 Aransiola TJ, Cavalcanti D, Ordoñez JA, *et al.* Current and Projected Mortality and Hospitalization Rates Associated With Conditional Cash Transfer, Social Pension, and Primary Health Care Programs in Brazil, 2000-2030. *JAMA Netw Open* 2024; **7**: e247519.

23 Stover J, Sonnevelft E, Tam Y, *et al.* The Effects of Reductions in United States Foreign Assistance on Global Health. 2025. DOI:10.2139/ssrn.5199076.

24 Brooke Nichols, Eric Moakley. Impact Dashboard - Impact Counter. Impact Count. Impact Counter. 2025. https://www.impactcounter.com/dashboard?view=table&sort=title&order=asc (accessed April 23, 2025).

25 Ahsan KZ, Angeles G, Choudhury A, *et al.* Accounting for aid: estimating the impact of United States' global health investments on mortality among women of reproductive age using synthetic control and Bayesian methods. *J Glob Health* 2025; **15**: 04067.

26 Brink D ten, Martin-Hughes R, Bowring AL, *et al.* Impact of an international HIV funding crisis on HIV infections and mortality in low-income and middle-income countries: a modelling study. *Lancet HIV* 2025; **12**: e346–54.

27 Charles Kenny, Justin Sandefur. How Many Lives Does US Foreign Aid Save? CGD. 2025; published online March 15. https://www.cgdev.org/blog/how-many-lives-does-us-foreign-aid-save (accessed April 23, 2025).

28 Weiss W, Piya B, Andrus A, Ahsan KZ, Cohen R. Estimating the impact of donor programs on child mortality in low- and middle-income countries: a synthetic control analysis of child health programs funded by the United States Agency for International Development. *Popul Health Metr* 2022; **20**: 2.

29 Lawlor DA, Tilling K, Davey Smith G. Triangulation in aetiological epidemiology. *Int J Epidemiol* 2017; : dyw314.

30 Höfler M. The Bradford Hill considerations on causality: a counterfactual perspective. *Emerg Themes Epidemiol* 2005; **2**: 11.

31 Lipsitch M, Tchetgen Tchetgen E, Cohen T. Negative Controls. *Epidemiology* 2010; **21**: 383–8.

32 Raymaekers K, Luyckx K, Moons P. A guide to improve your causal inferences from observational data. *European Journal of Cardiovascular Nursing* 2020; **19**: 757–62.

33 Khandker S, B. Koolwal G, Samad H. Handbook on Impact Evaluation. The World Bank, 2009 DOI:10.1596/978-0-8213-8028-4.

34 Strain T, Brage S, Sharp SJ, *et al.* Use of the prevented fraction for the population to determine deaths averted by existing prevalence of physical activity: a descriptive study. *Lancet Glob Health* 2020; **8**: e920–30.

35 Caro JJ, Briggs AH, Siebert U, Kuntz KM. Modeling Good Research Practices—Overview: A Report of the ISPOR-SMDM Modeling Good Research Practices Task Force-1. *Value in Health* 2012; **15**: 796–803.

36 Eddy DM, Hollingworth W, Caro JJ, Tsevat J, McDonald KM, Wong JB. Model Transparency and Validation: A Report of the ISPOR-SMDM Modeling Good Research Practices Task Force-7. *Value in Health* 2012; **15**: 843–50.

37 Briggs AH, Weinstein MC, Fenwick EAL, Karnon J, Sculpher MJ, Paltiel AD. Model Parameter Estimation and Uncertainty Analysis. *Medical Decision Making* 2012; **32**: 722–32.

38 Poverty, Prosperity, and Planet Report 2024: Pathways Out of the Polycrisis. Washington, DC: World Bank, 2024 DOI:10.1596/978-1-4648-2123-3.

39 Wolfson L. Estimating teh costs of achieving the WHO-UNICEF Global Immunization Vision and Strategy, 2006-2015. *Bull World Health Organ* 2008; **86**: 27–39.

40 Barros AJD, Wehrmeister FC, Ferreira LZ, Vidaletti LP, Hosseinpoor AR, Victora CG. Are the poorest poor being left behind? Estimating global inequalities in reproductive, maternal, newborn and child health. *BMJ Glob Health* 2020; **5**: e002229.

41 Fink G, Victora CG, Harttgen K, Vollmer S, Vidaletti LP, Barros AJD. Measuring Socioeconomic Inequalities With Predicted Absolute Incomes Rather Than Wealth Quintiles: A Comparative Assessment Using Child Stunting Data From National Surveys. *Am J Public Health* 2017; **107**: 550–5.

42 Silva AF, Dourado I, Lua I, *et al.* Income determines the impact of cash transfers on HIV/AIDS: cohort study of 22.7 million Brazilians. *Nat Commun* 2024; **15**: 1307.

43 Shattock AJ, Johnson HC, Sim SY, *et al.* Contribution of vaccination to improved survival and health: modelling 50 years of the Expanded Programme on Immunization. *The Lancet* 2024; **403**: 2307–16.

44 Checchi F, Roberts L. Documenting Mortality in Crises: What Keeps Us from Doing Better? *PLoS Med* 2008; **5**: e146.

45 Emergency Handbook: Mortality surveillance threshold. United Nations High Commissioner for Refugees (UNHCR), 2024 https://emergency.unhcr.org/emergency-assistance/health-and-nutrition/mortality-surveillance-threshold (accessed May 28, 2025).
